# Hsa-miRNA-765 as a Key Mediator for Inhibiting Growth, Migration and Invasion in Fulvestrant-Treated Prostate Cancer

**DOI:** 10.1371/journal.pone.0098037

**Published:** 2014-05-16

**Authors:** Yuet-Kin Leung, Queeny Kwan-Yi Chan, Chi-Fai Ng, Fanny Man-Ting Ma, Ho-Man Tse, Ka-Fai To, Jodi Maranchie, Shuk-Mei Ho, Kin-Mang Lau

**Affiliations:** 1 Department of Environmental Health, Center for Environmental Genetics, and Cancer Institute, University of Cincinnati Medical Center, Cincinnati, Ohio, United States of America; 2 Department of Anatomical and Cellular Pathology, The Chinese University of Hong Kong, Hong Kong Special Administrative Region, China; 3 Department of Surgery, The Chinese University of Hong Kong, Hong Kong Special Administrative Region, China; 4 State Key Laboratory in Southern China in Oncology, The Chinese University of Hong Kong, Hong Kong Special Administrative Region, China; 5 Department of Urology, University of Pittsburgh, Pittsburgh, Pennsylvania, United States of America; 6 Cincinnati Veteran Affairs Medical Center, Cincinnati, Ohio, United States of America; Northwestern University, United States of America

## Abstract

Fulvestrant (ICI-182,780) has recently been shown to effectively suppress prostate cancer cell growth *in vitro* and *in vivo*. But it is unclear whether microRNAs play a role in regulating oncogene expression in fulvestrant-treated prostate cancer. Here, this study reports *hsa-miR-765* as the first fulvestrant-driven, ERβ-regulated miRNA exhibiting significant tumor suppressor activities like fulvestrant, against prostate cancer cell growth via blockage of cell-cycle progression at the G2/M transition, and cell migration and invasion possibly via reduction of filopodia/intense stress-fiber formation. Fulvestrant was shown to upregulate *hsa-miR-765* expression through recruitment of ERβ to the 5′-regulatory-region of *hsa-miR-765*. HMGA1, an oncogenic protein in prostate cancer, was identified as a downstream target of *hsa-miR-765* and fulvestrant in cell-based experiments and a clinical study. Both the antiestrogen and the *hsa-miR-765* mimic suppressed HMGA1 protein expression. In a neo-adjuvant study, levels of *hsa-miR-765* were increased and HMGA1 expression was almost completely lost in prostate cancer specimens from patients treated with a single dose (250 mg) of fulvestrant 28 days before prostatectomy. These findings reveal a novel fulvestrant signaling cascade involving ERβ-mediated transcriptional upregulation of *hsa-miR-765* that suppresses HMGA1 protein expression as part of the mechanism underlying the tumor suppressor action of fulvestrant in prostate cancer.

## Introduction

The normal development and malignant growth of the prostate are regulated not only by androgens but also by estrogen [1]. The estrogen receptor (ER)β is the principal receptor expressed in the prostatic epithelium and in several stages of prostate cancer (PCa), including bone metastases [2], [3]. The synthetic estrogen diethylstilbestrol (DES), through its androgen-deprivation action, was once the frontline treatment for metastatic PCa [1], [4]. DES eventually lost favor because of its high cardiovascular toxicity and thromboembolic risk [5], [6], with parenteral estradiol-17β (E2) gaining recent popularity as a therapy for metastatic, castration-resistant PCa (CRPC) [7], [8] because of its low cardiovascular toxicity profile and protective action against osteoporosis [9]. Other selective ER modulators (e.g., tamoxifen, toremifene, and reloxifene) have been investigated in clinical trials but found to have limited efficacy as compared with DES [10]–[13]. With the approval in 2005 of fulvestrant (ICI 182,780), a pure estrogen receptor antagonist with no known agonistic action, for treatment of receptor-positive metastatic breast cancer, interest in its use for CRPC has emerged.

In preclinical models, fulvestrant has demonstrated features of a promising therapy for PCa. In an estrogen-induced PCa model [14]–[17], fulvestrant prevented the evolution of precancerous lesions, reversed the E2-induced transcriptome [16], [17], and induced its own gene signature [16]. In DU145, a human PCa cell line that expresses ERβ and no ERα, fulvestrant suppressed cell growth via the receptor [18] and regulated a unique set of genes, possibly through cross-talk between ERβ and NFκB [19]. Furthermore, fulvestrant suppressed the growth of DU145 and PC-3 xenografts through an ERβ-mediated KLF5 signaling pathway [20] and also inhibited LNCaP cell growth by downregulating the androgen receptor [21].

In the only phase II study of fulvestrant so far conducted [22], 20 CRPC patients received a loading-dose regimen (500 mg on day 0 and then 250 mg on day 14, day 28, and monthly thereafter). After six months of treatment, fulvestrant was well tolerated, although no favorable clinical or PSA response was observed [22]. However, by increasing the loading dose in the first month (500 mg every 14 days), the PSA level was effectively reduced by 40–99% within 0.27–2.67 months in six of the seven highly pretreated CRPC patients without any obvious toxicity [23]. This latter finding lends support to conducting more research in dose optimization and in-depth mechanistic studies of fulvestrant as a therapy for PCa.

MicroRNAs (miRNAs) are small (17–25 nucleotides) non-coding RNAs that regulate post-transcriptional gene expression. Each miRNA can bind to one or more target sequences in the 3′-untranslated-region of its target transcripts and elicit degradation of mRNA or suppression of protein translation, depending on the degree of complementary base pairing [24]. A single miRNA normally regulates expression of a large number of transcripts [25]–[27]. Aberrant expression of specific miRNAs confers a growth advantage to cancer cells over normal cells by disrupting multiple oncogenic/tumor-suppressor pathways. PCa-specific miRNAs have been identified [28]–[30], and some are androgen-related [31]. To date, however, no single miRNA has been linked to estrogens or antiestrogens in PCa.

Here, we examined the role of miRNA in mediating the action of fulvestrant in PCa. Global profiling of miRNA expression in DU145 cells identified *hsa-miR-765* as a fulvestrant-regulated miRNA. Promoter analyses defined a minimal sequence in the 5′-regulatory region of *hsa-miR-765* that recruits ERβ and that is critical for fulvestrant regulation. The effects of the miRNA on PCa cell growth, migration, and invasion were compared with those of fulvestrant. The dependency of fulvestrant actions on ERβ was demonstrated by knockdown experiments. The change in expression of *hsa-miR-765* and its downstream oncogenic protein, high-mobility group AT-hook 1 (HMGA1), was assessed in prostatectomy specimens obtained from patients after they had been treated with fulvestrant for one month.

## Materials and Methods

### Fulvestrant treatment

DU145 and PC3 cell lines were purchased from ATCC (Manassas, VA). DU145 cells (ATCC) were maintained as previously described [18]. PC3 (ATCC) cells were cultured in RPMI 1640 with 10% heat-inactivated FBS (hiFBS). The identity of each cell line has recently been authenticated by ATCC using short tandem repeat profiling method. All cells were maintained in 5% charcoal-stripped hiFBS medium for 24 h before drug treatment. The cells were treated with either 10^−6^ M fulvestrant in 0.1% or 0.1% ethanol. Control cultures were treated with vehicle only.

### MiRNA and gene expression

For miRNA profiling, total RNAs were extracted and labeled directly using the NCode Rapid Labeling System (Invitrogen, Grand Island, NY) and arrayed on the NCode Human miRNA Microarray V3 (Invitrogen).

Tissue expression of miRNA was studied by extracting total RNAs from cryosections (5–10 µm) using RNAzol RT (Molecular Research Center, Cincinnati, OH), poly(A)-tailed and reverse transcribed with universal RT primer using the NCode microRNA first-strand cDNA kit (Invitrogen). Real-time PCR was conducted with SYBRGreen PCR Master-Mix (Invitrogen) using either the *hsa-miR-765* specific or spliceosomal U6 small nuclear RNA (RNU6)-specific qRT forward primer (Table S2) and a universal reverse qPCR primer (Invitrogen).

Total RNAs were prepared with random hexamer (Invitrogen). Ribosomal protein 3 (RPS3, Table S2) was used as the housekeeping control. Relative gene expression was determined by the ΔΔC_T_ method [32].

### Clinical specimens

Patients with histologically confirmed, clinically localized PCa were given a single intramuscular injection of 250 mg of fulvestrant, 28 days before a scheduled radical retropubic prostatectomy. Patients were excluded if they had a white blood cell count <3,000/µl, a platelet count <100,000/µl, hemoglobin <11 g/dl, INR>1.6, or bilirubin AST or creatinine levels >1.5 times the upper limit of normal. Patients also were excluded if they required corticosteroids for the treatment of other systemic diseases or had a history of congestive heart failure, active angina, infection, or active second malignancy. All subjects had a final Gleason sum of 6 or 7 at prostatectomy. Specimens from untreated patients with a similar Gleason sum and all specimens from fulvestrant-treated patients were obtained from University of Massachusetts Medical School (UMMS) under a protocol (PI. Dr. Maranchie) approved by the Committee for the Protection of Human Subjects in Research and Institutional Review Board at UMMS. All subjects provided written informed consent to participate in this study and they were de-identified.

### Knockdown of ERβ

SiRNAs for ERβ or scramble siRNA (Invitrogen) was transfected into DU145 cells (2×10^5^) using X-tremeGENE (Roche, Indianapolis, IN). The siRNA-treated cells were treated with either fulvestrant or ethanol for another 2–4 days and then subjected to real-time RT-PCR, promoter activity analysis, cell growth assay, and F-actin staining.

### 5′-regulatory region analyses

The 5′ upstream genomic regions of *hsa-miR-765* precursor (Chr1:156,906,923–156,906,036 Accession no. NC_000001.10) from −3208 to +100 was amplified and cloned into pGL3-basic (Promega, Madison, WI) as *pGL3-hsa-miR-765*. Serial deletions from the 5′ end of the cloned sequence in the vector were conducted to generate pGL3-miR-765-Δ1192 bp (DNA sequence from −2016 to +100), pGL3-miR-765-Δ1766 bp (DNA sequence from −1442 to +100), pGL3-miR-765-Δ2414 bp (DNA sequence from −792 to +100), pGL3-miR-765-Δ2618 bp (DNA sequence from −590 to +100), pGL3-miR-765-Δ2972 bp (DNA sequence from −236 to +100), and pGL3-miR-765-Δ3113 bp (DNA sequence from −95 to +100). Reporter activities of other truncated or mutated vectors in DU145 cells were determined with or without fulvestrant and/or ERβ siRNA knockdown using the dual luciferase reporter assay system (Promega).

### MiRNA targeting reporter assay

DU145 cells were transfected with luciferase reporter vector pMIR-miR-765 that was cloned with complementary sequence of hsa-miR-765 as perfect miR-765 target and then treated with either hsa-miR-765 mimic or negative-control mimic. The lysates were subjected to the dual luciferase reporter assay. The 3′-translational region of *high- mobility group AT-hook 1* (*HMGA1*) gene (+8026–+9332) was generated by PCR (primers in Table S2) and cloned into pMIR-REPORT (Invitrogen) as *pMIR-HMGA1-3UTR*. The *hsa-miR-765* mimic (sequence in Table S2) was cloned into pMIR as *pMIR-miR-765*. The effect of the *hsa-miR-765* mimic on luciferase expression was determined by luciferase assay (Promega), with *pMIR-empty* included as a control.

### Chromatin immunoprecipitation assay

Chromatin immunoprecipitation (ChIP) assays were performed according to published methods [19]. In brief, DU145 cells were treated with fulvestrant or control for 45 min. DNA-protein complexes were crosslinked with 1% formaldehyde. Nuclear complexes were sonicated (250–500 bp). Five micrograms of mouse IgG (Millipore, Billerica, MA), anti-RNA polymerase II (Millipore) or anti-ERβ1 (Serotec, Raleigh, NC) antibodies were applied for overnight immunoprecipitation. The DNA-protein complexes were washed and eluted. Immunoprecipitated DNAs were cleaned up, reverse-crosslinked, and purified. PCR and realtime PCR revealed the recruitment of ERβ to a sequence in the 5′-regulatory region of *hsa-miR765* (primers listed in Table S2). The *0N* promoter of ERβ [33] was used as a non-ERβ binding control for this experiment.

### Cell-growth assay

Effects of fulvestrant or *hsa-miR-765*–mimic treatment on DU145 or PC3 cell growth were determined by the CellTiter 96 Non-radioactive Cell Proliferation Assay (Promega). For HMGA1 ectopic expression experiment, full length of HMGA1 (pCMV6-AC-HMGA1, OriGene, Rockville, MD) or a negative-control (pCMV6-AC) were transfected into DU145 cells. The transfected cells were enriched in G418-supplemented medium for a week. Relative growth of DU145 cells co-treated with fulvestrant for 4 days and either HMGA1 expression or empty vector relative to the control cells treated with ethanol and empty vector were compared.

### Flow cytometry analyses

DU145 cells were treated with fulvestrant/ethanol or *hsa-miR-765* mimic/negative-control mimic for 2 days. The treated cells were analyzed according to published protocols [32].

### Western blot analyses

Five micrograms of protein from cell lysate were electrophoresed on 10–12.5% SDS-PAGE and subjected to Western blot analysis. Primary antibodies listed in Table S3 were used to detect protein levels.

### Migration and invasion assays

DU145 or PC3 cells were treated with fulvestrant or *hsa-miR-765* mimic and their respective controls for 2 days. Wound-healing assays were performed as previously reported [34]. DU145 cells were pretreated with fulvestrant or ethanol for 5 h before migration and invasion assays were performed [34].

### Filamentous-actin (F-actin) staining

F-actin in fulvestrant- or ethanol-treated DU145 cultures subjected to RNAi-mediated knockdown of ERβ, *hsa-miR-765*-mimic transfection, or control treatment were visualized as previously described [34]. In brief, the fulvestrant- and ethanol-treated control DU145 cells with either ERβ siRNA or negative-control siRNA were stained with TRITC-conjugated phalloidin, and the fluorescence images were captured.

### Computational prediction of miRNA targets

MiRanda/mirSVR [35] (http://www.microrna.org), TargetScan r5.2 [36] (http://www.targetscan.org), RNAhybrid [37], and EIMMo2 [38] were used to predict the putative targets of *hsa-miR-765*. Genomic alignments, BLAT (http://www.ensembl.org), were used for additional validation of predicted sites.

### Immunostaining of HMGA1 and AR

Frozen sections (5 µm) of PCa specimens from patients treated or not treated with fulvestrant prior to prostatectomy were fixed in 3% formaldehyde at room temperature and then with methanol at −20°C. HMGA1 and AR was immunodetected with 1∶100 anti-HMGA1 antibody (sc 8982, Santa Cruz Biotechnology, Santa Cruz, CA) and anti-AR respectively (sc 816, Santa Cruz Biotechnology, Santa Cruz, CA) according to published protocols [39]. Immunopositivity was determined by the percentage of positive signal (nuclear or cytoplasmic) in Gleason grade 3/4 foci.

## Results

### A. Fulvestrant inhibits cell growth, cell-cycle progression, migration, and invasion in an ERβ-dependent manner

**Figure 1:**
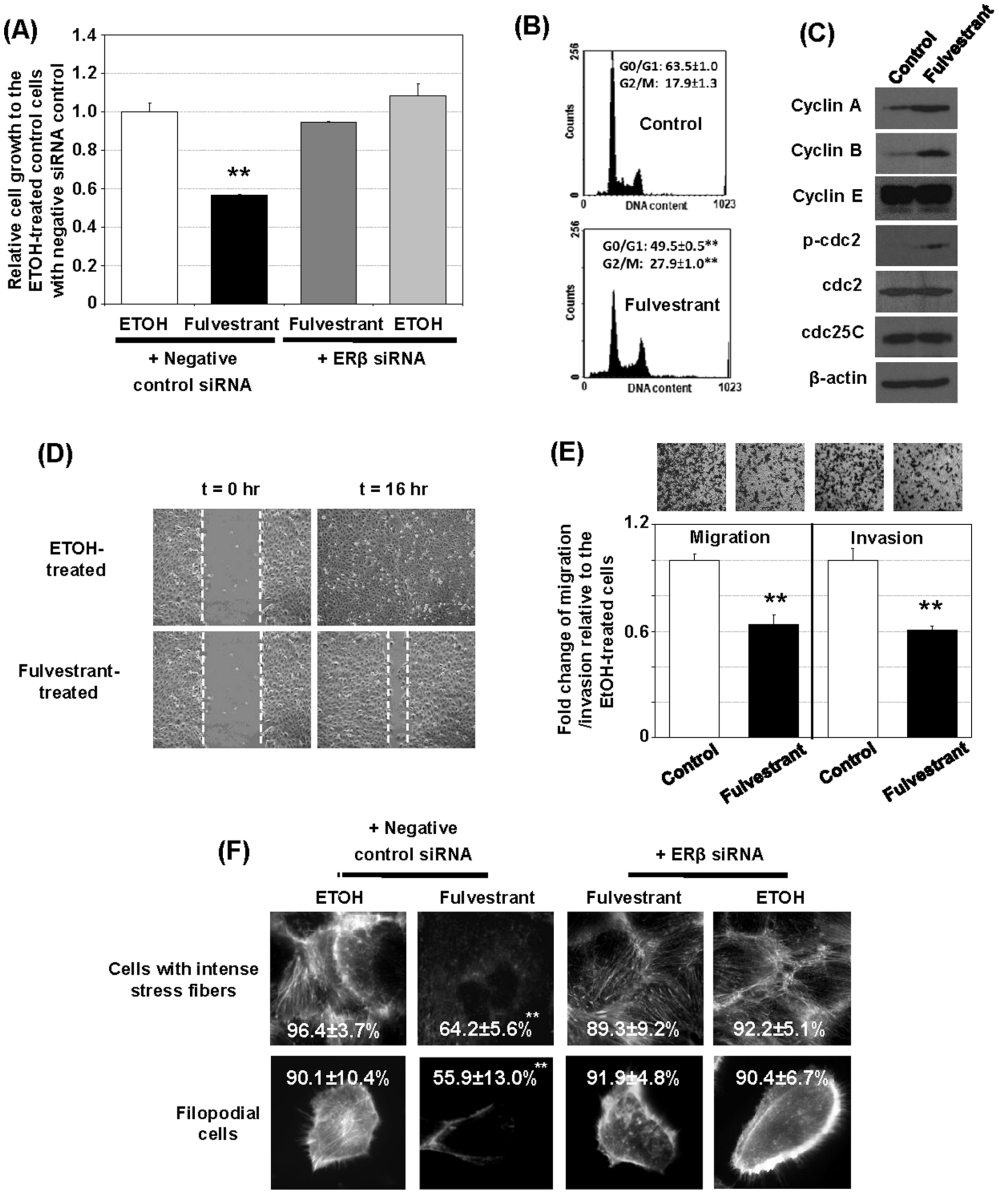
Fulvestrant inhibits DU145 cell growth, migration, and invasion. (A) Fulvestrant induces growth inhibition of DU145 cells via an ERβ-dependent mechanism. Growth of the fulvestrant-treated DU145 cells with or without ERβ siRNA knockdown for 4 days relative to the ethanol-treated control cells with negative-control siRNA are presented and compared (n = 8). ERβ expression was also knocked down by another siRNA (siRNA#2) and the similar results were obtained (Figure S5). (B) Fulvestrant induces DU145 cell-cycle arrest at G2/M phase. Representative DNA histograms of 48 hrs fulvestrant -or ethanol- (control) treated cells and percentage distributions of the cells at G0/G1 and G2/M phases (n = 3) are presented and compared. (C) Fulvestrant induces expression of G2/M markers. DU145 cells were treated with fulvestrant or ethanol for 2 days (control) and cell cycle markers were determined by Western blot analysis. Two independent experiments were performed and one representative set of data was presented. (D) Fulvestrant suppresses cell migration. A wound-healing assay was performed on the fulvestrant- and ethanol (EtOH)-treated DU145 cells (n = 3). Representative micrographs of the fulvestrant- and ethanol-treated cell cultures with scratches at 0 h and after 16 h are shown. The wound is marked by dotted lines. (E) Fulvestrant inhibits transwell migration (left panel) and invasion (right panel) in DU145 cells (n = 3) after 5 hrs of fulvestrant treatment. (F) Reductions of filopodial cells and cells with intense stress fibers by fulvestrant (treated with 48 hrs) via an ERβ-dependent mechanism. Representative micrographs and the percentages of the cells with intense stress fibers and the filopodial cells (n = 3) are presented. Student t-test was performed to determine significance with a cutoff p value of 0.05. ** p<0.01; bars = S.D.

Fulvestrant inhibited the growth of DU145 cells by 40% and PC-3 cells by 30% through an ERβ-dependent pathway (Figure 1A, Figure S1A) and arrested cell division at the G2/M phase, as indicated by a significant decrease in the G0/G1 cell population and an accumulation of G2/M cells (Figure 1B). The disruption in cell-cycle progression was accompanied by enhanced expression of the G2/M markers cyclin A (G2), cyclin B (M), and phosphorylated cdc2 (G2) but not of the S-phase markers cyclin E and cdc25C (Figure 1C).

Fulvestrant inhibited cell migration in the wound-healing assay (Figure 1D), and transwell migration (Figure 1E, left panel) and cell invasiveness in the transwell invasion assay (Figure 1E, right panel) by ∼40% (*p*<0.01, n = 3) in DU145 cells (Figure 1E) as well as in PC-3 cells (Figure S2). Treatment with fulvestrant significantly reduced the percentage of filopodial cells from 90.1% to 55.9% (*p*<0.005, n = 5) and of cells with intense stress fiber from 96.4% to 64.2% (*p*<0.001, n = 5) (Figure 1F). RNAi-mediated knockdown of ERβ effectively reversed the fulvestrant-induced inhibition (Figure 1F).

### B. Fulvestrant upregulates *hsa-miR-765* expression in PCa cells

**Figure 2:**
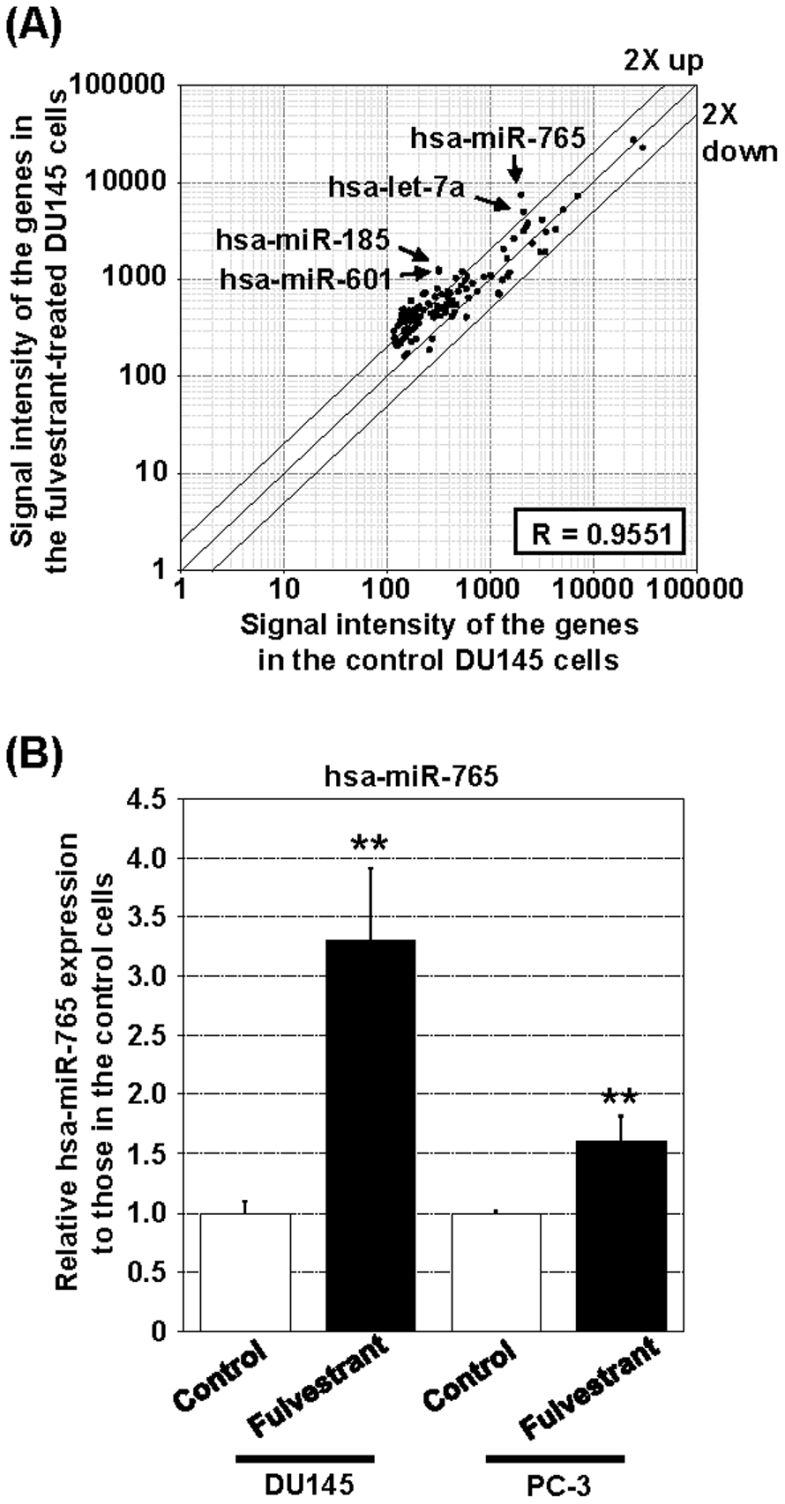
Fulvestrant upregulates *hsa-miR-765* expression in DU145 cells. (A) *Hsa-miR-765* is highly expressed in fulvestrant-treated DU145 cells. Total RNAs of treated cells were labeled directly and arrayed on the NCode Human miRNA Microarray. The median- and linear regression-normalized data are presented in a scatterplot. (B) *Hsa-miR-765* is induced by fulvestrant in two prostate cancer cell lines. The hsa-miR-765 in the fulvestrant- and ethanol-treated control DU145 and PC-3 cells was quantified by miRNA qRT-PCR analysis. Relative fold changes between the expression of *hsa-miR-765* in the fulvestrant-treated and control cells are presented. Student t-test was performed to determine their significance using a cutoff p value of 0.05 (n = 3). **p<0.01; bars = S.D.

Among the 211 detectable miRNAs, 6 highly abundant miRNAs including hsa-miR-185, hsa-let-7b, hsa-miR-765, hsa-let-7a, hsa-miR-601, and hsa-miR-768-5p (>300 relative normalized signal intensity) were upregulated after fulvestrant-treatment (>2-fold; *p*<0.005) (Figure 2A). *Hsa-miR-765* was one of the miRs that exhibited the greatest relative increase (3.8-fold) and greatest absolute level of expression in fulvestrant-treated DU145 cells (Table S1). Fulvestrant also induced a significant increase in the expression of this miR in PC3 cells in an ERβ-dependent manner (Figure 2B, Figure S1B).

### C. *Hsa-miR-765* inhibits cell growth, cell-cycle progression, migration and invasion

**Figure 3:**
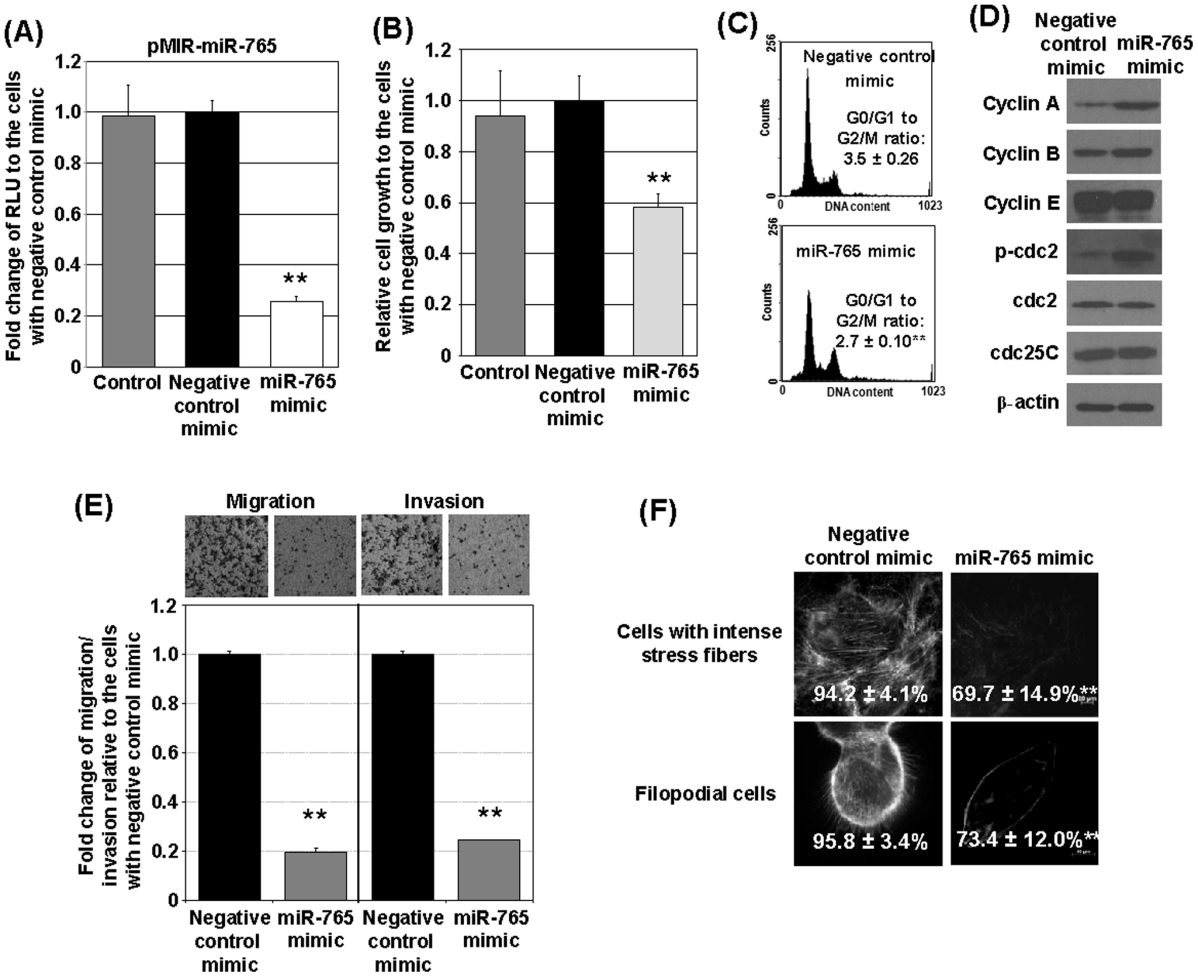
*Hsa-miR-765* suppresses DU145 cell growth, migration, and invasion. (A) *Hsa-miR-765* mimic effectively recognizes reporter with complementary sequence of *hsa-miR-765* in DU145 cells. Fold changes of luciferase activities of the *hsa-miR-765* mimic treated cells relative to the cells treated with the negative-control mimic are presented (n = 3). Transfection reagents were used as control. (B) *Hsa-miR-765* mimic reduces DU145 cell growth. MTS assay was performed on the cells treated with *hsa-miR-765* mimic or negative-control mimic or transfection control for 4 days (n = 8). (C) *Hsa-miR-765* mimic significant reduces G0/G1 to G2/M ratio in DU145. Representative DNA histograms (n = 3) are presented. (D) *Hsa-miR-765* mimic treatment causes up-regulation of cyclin A, cyclin B, and phosphorylated-cdc2 expression in DU145 cells. Protein expression levels of cell cycle regulator proteins were determined by Western blot analyses. Two independent experiments were performed and one representative set of data was presented. (E) *Hsa-miR-765* mimic suppresses DU145 cell migration and invasion as shown in transwell migration assay (top left) and invasion assay (top right), respectively. Representative micrographs of the cells after transwell migration (top left) or invasion assay (top right) are presented. Fold changes of migration (bottom left) and invasion (bottom right) of DU145 cells with either *hsa-miR-765* mimic or negative-control mimic relative to the control cells with negative-control mimic are presented (n = 3). (F) *Hsa-miR-765* mimic significantly reduces stress fibers and filopodia formations in DU145 cells. Representative micrographs and the percentages of the cells with intense stress fibers and the filopodial cells (n = 3) are presented. Student's t-test was used for comparisons with a cutoff p value of 0.05. ** p<0.01; bar = S.D.

A *hsa-miR-765* mimic or a negative-control was expressed in DU145 cells carrying the luciferase reporter vector *pMIR-miR-765*. Ectopic expression of the *hsa-miR-765* mimic, but not the negative-control, effectively suppressed luciferase activity by >70% in DU145 cells (Figure 3A). Overexpression of the *hsa-miR-765* mimic in DU145 cells induced inhibition of cell growth (40%; Figure 3B) and cell-cycle arrest at G2/M (G0/G1 to G2/M ratio decreased from 3.5±0.26 to 2.7±0.10, *p* = 0.0074) (Figure 3C) and upregulated the expression of cyclin A, cyclin B, and phosphorylated-cdc2 but not cyclin E or cdc25C (Figure 3D). Finally, it decreased cell migration and invasiveness (∼80%; Figure 3E), and the formation of filopodia/intense stress fiber (Figure 3F) at levels greater than or comparable to those induced by fulvestrant (see Figures 1C & 1D). Overall, the actions of *hsa-miR-765* in DU145 cells are highly similar to those of fulvestrant. In addition to DU145 cells, the inhibitory effects of has-miR-765 mimic were also observed in PC-3 cells (Figure S3).

### D. Fulvestrant induces upregulation of *hsa-miR-765* expression via recruitment of ERβ to a putative regulatory element

**Figure 4:**
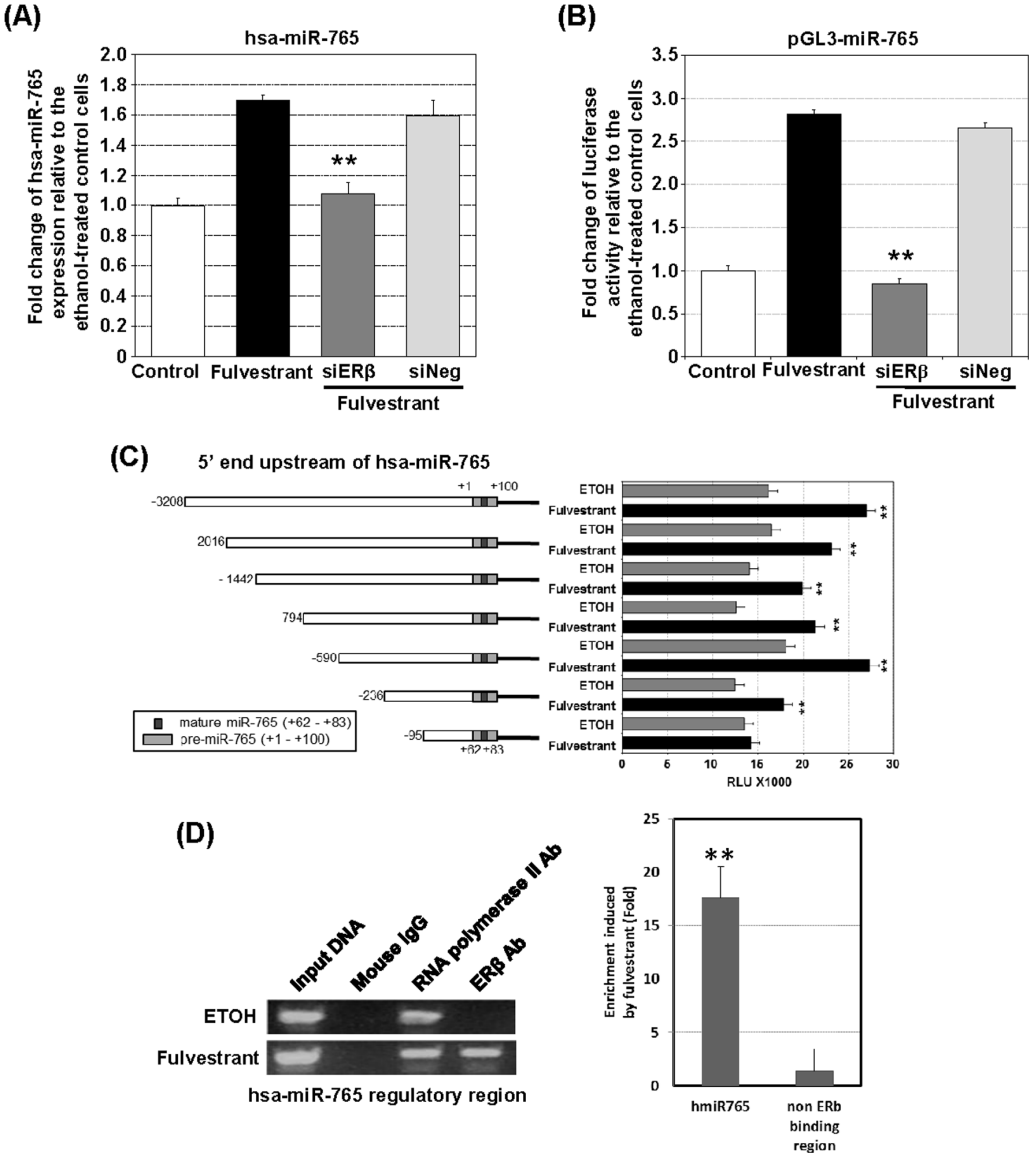
ERβ is involved in fulvestrant-induced upregulation of *hsa-miR-765* expression. (A) ERβ siRNA knockdown blocks fulvestrant-induced upregulation of *hsa-miR-765* expression in DU145 cells. Expression levels of *hsa-miR-765* determined by qRT-PCR analysis of the fulvestrant-treated cells with ERβ-siRNA (siERβ) or scramble negative-control (siNeg) were compared (n = 3). (B) SiRNA knockdown of ERβ blocks fulvestrant-induced transactivation of the 5′ upstream regulatory region of *hsa-miR-765* in DU145 cells. 5′ upstream regulatory region of *hsa-miR-765* was cloned into a luciferase vector. The reporter activities with ERβ-knockdown (siERβ) or scramble negative-control (siNeg) in the presence of fulvestrant were compared (n = 3). (C) Deletion mapping analysis defines a fulvestrant-responsive segment in *hsa-miR-765* regulatory region in DU145 cells. The 5′ upstream DNA sequence of *hsa-miR-765* from nt. −3208 to +100 was analyzed using luciferase reporter system. Serial deletions from the 5′ end of the cloned sequence in the vector were conducted. Reporter activities were compared between the fulvestrant-treated (Fulvestrant) and control (ETOH) cells for each reporter vector (n = 3). (D) Fulvestrant-induces recruitment of ERβ onto the putative *hsa-miR-765* regulatory region. Chromatin-immunoprecipitation revealed the recruitment of ERβ to a sequence in the 5′-regulatory region of *hsa-miR765*. Mouse IgG and RNA polymerase II serve as negative and positive control, respectively. Fulvestrant induced 17 fold increase in ERβ recruitment when compared with non-ERβ binding region (the 0N promoter of ERβ [33], [41]). Student's t-test was performed to determine significance of between groups using a cutoff p value of 0.05. ** p<0.01; bar = S.D.

DU145 cells were subjected to siRNAs-mediated knockdown of ERβ prior fulvestrant treatment. SiRNA effectively knocked down ERβ expression (Figure S4). This knockdown completely blocked fulvestrant-induced enhancement of *hsa-miR-765* expression (Figure 4A) and abolished the transactivation activities of fulvestrant on a 5′-regulatory sequence of *hsa-miR-765* (between −3208 and +100; 3.3 kb) (Figure 4B). Serial deletion analysis identified a 141 bp sequence (between −236 and −95) within the 3.3-kb 5′-regulatory sequence as essential in mediating the stimulatory effect of fulvestrant on *hsa-miR-765* transcription. ChIP assays further demonstrated recruitment of ERβ to this short sequence following fulvestrant stimulation (Figure 4D). These data support a role of ERβ in mediating fulvestrant-induced *hsa-miR-765* upregulation.

### E. HMGA1 is a target of *hsa-miR-765*

**Figure 5:**
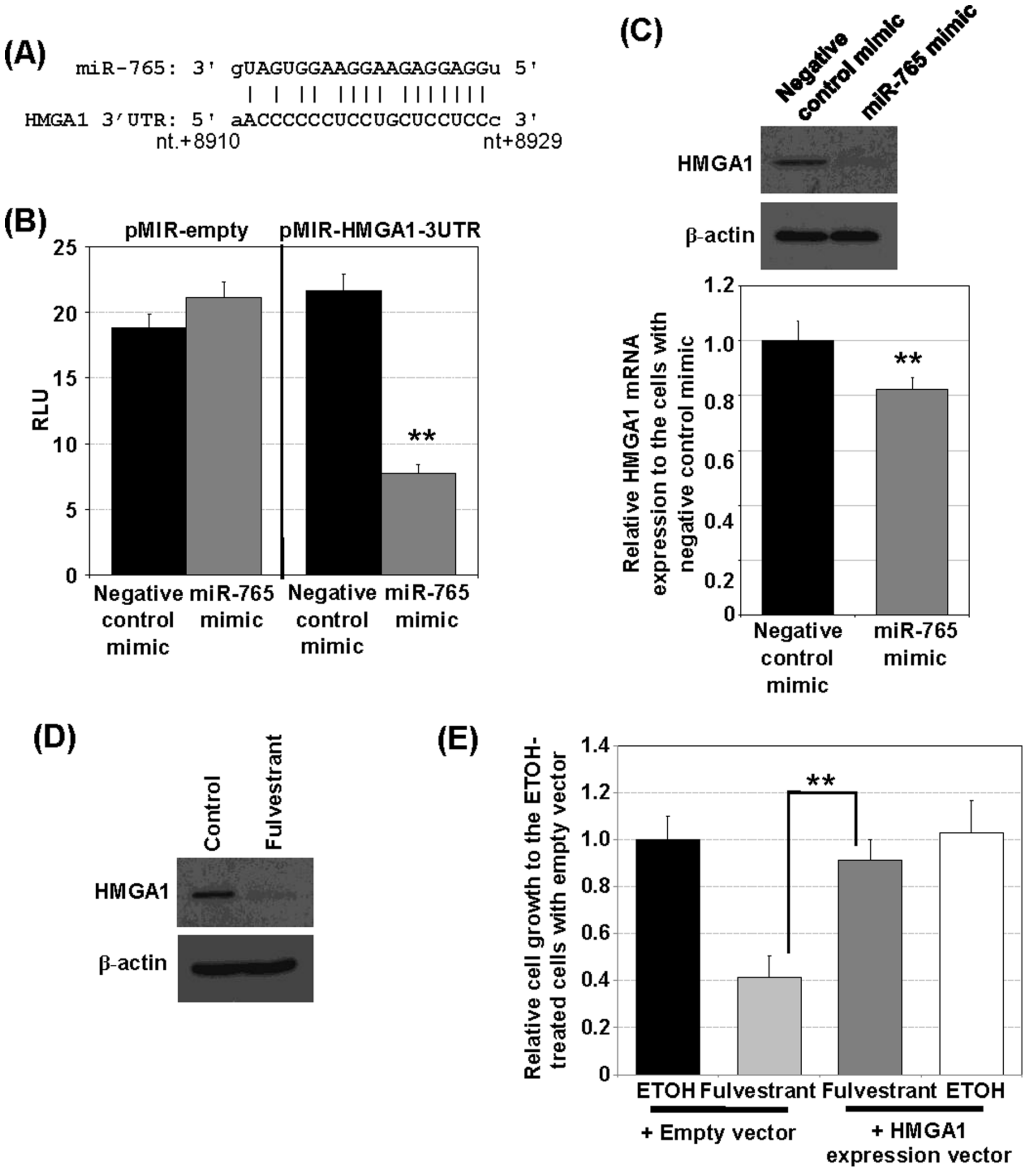
HMGA1 is a direct target of *hsa-miR-765*. (A) The 3′UTR of *HMGA1* from +8910 to +8929 is predicted to be *hsa-miR-765* binding site. (B) *Hsa-miR-765* interacts with 3′UTR of *HMGA1* in a targeting reporter assay. DU145 cells were transfected with either pMIR-empty or pMIR-HMGA1-3UTR in which 3′ UTR of *HMGA1* (+8026–+9332) was cloned into the 3′ end of luciferase. Reporter activities of the pMIR-HMGA1-3UTR transfected cells treated with *hsa-miR-765* mimic or negative-control mimic are compared (n = 3). (C) *Hsa-miR-765* mimic reduced HMGA1 protein expression in DU145 cells. Protein and mRNA levels of HMGA1 in the *hsa-miR-765* mimic- and negative-control mimic-treated cells were determined by Western blot analysis (upper) and real-time RT-PCR analysis (lower), respectively. Results from *miR-765* mimic vs negative control mimic are compared (n = 3). (D) Fulvestrant reduces HMGA1 protein expression in DU145 cells. Protein level of HMGA1 and β-actin in the fulvestrant-treated and ethanol-treated control (CTL) cells were determined by Western blot analysis. (E) Ectopic expression of HMGA1 blocks fulvestrant-induced DU145 cell growth inhibition. The relative cell growth was determined after 4 days of treatment with fulvestrant or ethanol after stable transfection of *HMGA1* (or empty vector for control) for a week. Protein levels of HMGA1 were shown in Figure S6. The cell growth of fulvestrant-treated cells with HMGA1 overexpression vs empty vector are compared (n = 8). Student's t-test was performed to determine significance between groups using a cutoff p value of 0.05. **p<0.01; bar = S.D.

Bioinformatic analyses with multiple miRNA target prediction programs suggested HMGA1 as a target of *hsa-miR-765*; a conserved recognition site at +8982 to +9002 with −22.6 kcal/mol of minimum free energy was predicted (Figure 5A, Table S4). Reporter assays demonstrated that transfection of *hsa-miR-765* mimic, but not of a negative-control mimic, significantly decreased *HMGA1 3′ UTR*-dependent luciferase activity (Figure 5B); no such difference was observed in DU145 cells carrying the pMIR-empty vector. Importantly, transfection of the *hsa-miR-765* mimic completely blocked the expression of HMGA1 protein (Figure 5C upper panel), along with a slight reduction in the mRNA level (Figure 5C, lower panel). Of interest, treatment with fulvestrant also effectively shut down the expression of HMGA1 protein (Figure 5D). These data support an inhibitory role of *hsa-miR-765* on HMGA1 expression at the protein level and a mediator role in fulvestrant action on PCa cells. Finally, ectopic expression of HMGA1 effectively reduced the growth inhibitory effect of fulvestrant on DU145 cells (Figure 5E), a finding consistent with the reported oncogenic action of HMGA1 in the prostate [40].

### F. *Hsa-miR-765* is elevated but HMGA1 protein is reduced in fulvestrant-treated PCa specimens

**Figure 6:**
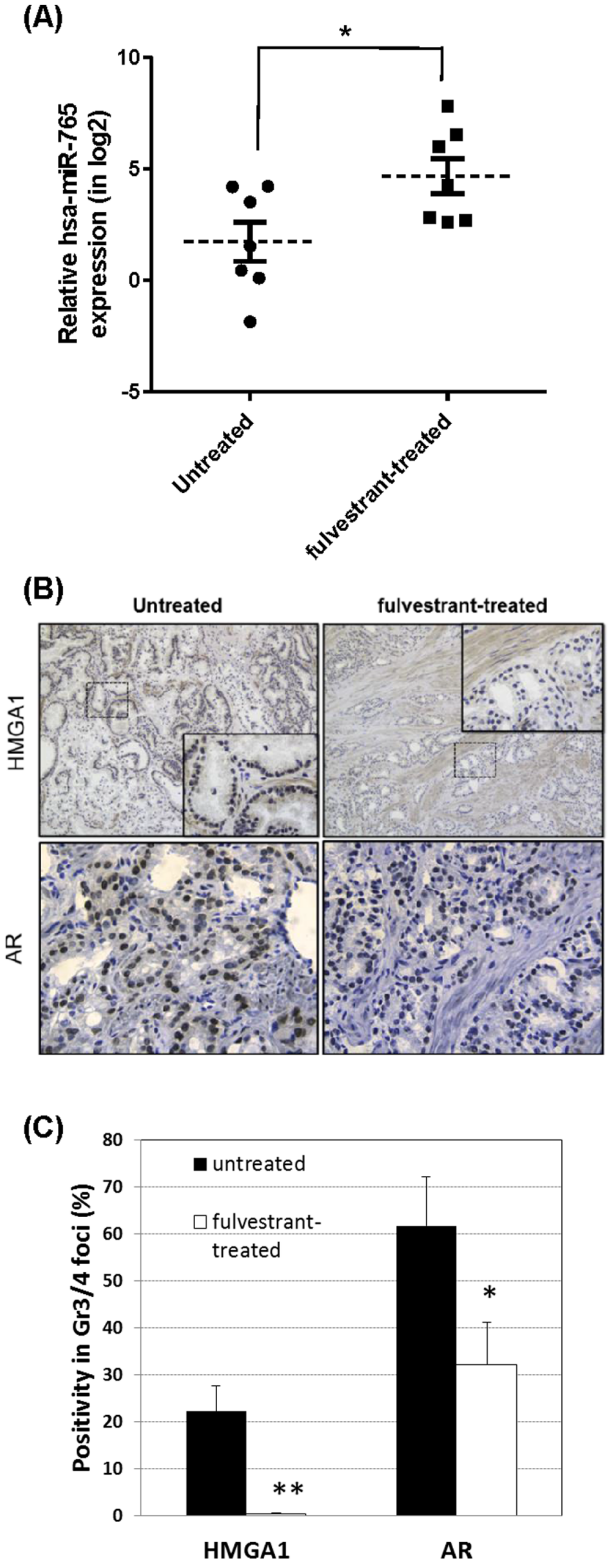
Significant reduction of HMGA1 protein correlates with enhanced expression of *hsa-miR-765* in fulvestrant-treated clinical PCa specimens. (A) Higher level of *hsa-miR-765* is detected in fulvestrant-treated clinical PCa specimens. Relative fold changes between expression of *hsa-miR-765* in the fulvestrant-treated (n = 7) and untreated (n = 7) clinical specimen are presented. Student's t-test was performed to determine significance between two groups. *p<0.05; bar = S.E.M. (B) Nuclear expression of HMGA1 and AR is reduced in fulvestrant-treated clinical PCa specimens. HMGA1 immunostaining was performed in the clinical PCa specimens from the fulvestrant-treated (n = 5) and untreated (n = 5) patients. Representative micrographs (100×) are shown. In upper panel, a magnified view (400×) of a selected region (dashed rectangle) in each micrograph is shown as a small insert to show the immunostaining of HGMA1 in the nuclei of Gleason grade 3/4 cancer foci. Imunnopositivity of nuclear AR is reduced in fulvestrant-treated Gleason grade 3/4 foci as shown in lower panel (400×). (C) Expression of both HMGA1 and AR is significantly reduced in fulvestrant-treated clinical PCa specimens when compared with their respective untreated samples (*p<0.05; **p<0.01; n = 9 (from 5 patients) for untreated samples; n = 10 (from 5 patients) for fulvestrant-treated samples, bar = S.E.M).

Prostatectomy tissues were obtained from patients with localized PCa who received or not received fulvestrant treatment (250 mg, i.m. 28 days before prostatectomy). Real time RT-PCR analyses showed upregulation of *hsa-miR-765* mRNA (2.7-fold, *p*<0.05, n = 7) in the PCa specimens from fulvestrant-treated patients as compared with those from untreated controls (n = 7) (Figure 6A). In contrast, immunohistological analyses revealed a marked reduction in HMGA1 immunopositivity in specimens from fulvestrant-treated patients (Figure 6B). HMGA1 was localized primarily in the nuclei of PCa cells in cancer foci, with only weak cytoplasmic staining in stromal cells. Immunostaining was negligible in fulvestrant-treated specimens in almost all cancer foci (Figure 6C, *p*<0.01, n = 5). Androgen receptor was previously shown to be downregulated by fulvestrant in a rodent [41] and a cell model [21]. Here, we showed AR was also significantly downregulated in our clinical study (Figure 6B and C). However, as we reported previously, ERβ was significant loss in these foci [33] and fulvestrant treatment did not affect the ERβ level (data not shown). These results, taken together, indicate that fulvestrant enhanced *hsa-miR-765* expression but suppressed HGMA1 and AR protein expression in PCa specimens from treated-patients.

## Discussion

In the present study, we identified a novel mechanism of action of fulvestrant that is mediated by ERβ-dependent upregulation of *hsa-miR765*, a miRNA with strong anti-PCa action. Deletion analysis identified a short sequence that interacts with fulvestrant (∼140 bp) within a 3.3 kb 5′-regulatory region of *hsa-miR765*. Interestingly, on the basis of prediction analysis, this short sequence does not harbor any known ERβ interacting elements. Yet, fulvestrant can promote recruitment of ERβ to the sequence. One possible explanation is that fulvestrant recruits the receptor via tethering [19], [20], [42]–[44] but to an unknown tethering partner of ERβ, whose identity remains to be uncovered.

Bioinformatics analyses of the ERβ binding region have been performed based on TRANSFAC database. No consensus full or half ERE was found. Furthermore, no previously reported ERβ tethering sites such as SP1, AP1 and FOXA1 were found. However, a putative PAX5 binding site was identified. PAX5 is a transcription factor responsible for regulating B cell lineage. We have performed multiple ChIP and re-ChIP assays (data not shown) and could not establish either recruitment of PAX5 to this region nor demonstrate tethering of ERβ to the putative PAX5 binding site. This is not necessary a surprise finding because the characterization of cristrome for ERβ has not been fully completed.

MiRNAs function as negative post-transcriptional regulators for gene expression through imperfect binding to the 3′-UTR of target genes, causing translational repression and/or rapid transcript degradation [29], [45]. In this study, we identified and validated HMGA1 as a target of *hsa-miR-765* in PCa cells. Consistent with our finding is the significant reduction of DU145 cell growth by RNAi-mediated knockdown of HMGA1 [40]. In contrast, ectopic expression of HMGA1 in non-metastatic Dunning PCa cells increased *in vitro* and *in vivo* growth and enhanced *in vitro* invasion [46]. In clinical PCa tissues, high expression of HMGA1 was associated with high Gleason grades, invasiveness, and recurrence [47], [48]. Collectively, these findings implicated HMGA1 as playing an oncogenic role in PCa that can be blocked by fulvestrant or a *hsa-miR-765* mimic.

Current investigations of miRNAs in PCa focused primarily on the identification of aberrantly expressed miRNAs as diagnosis/prognosis biomarkers [28], [29], [45], [49]. However, a more interesting study would address the identification of miRNAs that can be used to predict therapeutic responses. To date, only a few studies have addressed the modulation of miRNA expression in PCa cells in response to hormones (androgen ablation therapy) [49]–[52] and radiation [53], [54]. Our findings of the anti-PCa action of fulvestrant via upregulation of *hsa-miR-765* provide new mechanistic insights to the action of this antiestrogen and an invaluable tool for monitoring fulvestrant-responsiveness in patients. Future identification of additional fulvestrant-regulated miRNAs and their downstream targets would enhance the development of effective PCa therapies beyond the antiestrogen.

Preclinical studies demonstrated that fulvestrant is effective in inhibiting the growth of PCa cells in culture [18]–[21] and as xenografts [20] and in preventing the development of PCa in animal models [17]. However, the mechanism of action of fulvestrant remains unclear, except for a few earlier reports suggesting that ERβ is its key mediator [18]–[20]. In this study, we confirmed that fulvestrant exerts antitumor effects on DU145 cells via ERβ signaling. This finding is consistent with several reports on the anti-PCa action of ERβ, such as inhibition of epithelial-mesenchymal transition [55], downregulation of VEGF-A [55] and TMPRSS2-ERG [56], reduction of PC3 cell migration and invasion *in vitro* [39], and growth of DU145 and PC3 xenografts in nude mice [20]. Other proposed mechanisms of action of fulvestrant include cross-talk of ERβ with the NFκB [19] or the KLF5 [20] signaling pathways to mediate cell death or anoikis, respectively, as well as suppression of androgen-receptor expression [21], [41] and its action [21].

Although no favorable clinical outcome was noted in the only fulvestrant clinical II trial, of 20 patients with CRPC, using a loading dose regimen (500 mg on day 0 and 250 mg thereafter) for six months [22], a smaller study using a higher dose in the first month (500 mg every 14 days) produced a marked reduction in PSA (40–99%) in six of the seven CRPC patients [23]. Our clinical study was the only one that gave fulvestrant (250 mg) 28 days before prostatectomy to patients with PCa who had clinically localized low-grade disease. It was gratifying to observe a significant elevation in *hsa-miR-765* and an almost complete loss of HMGA1 and significant reduction of AR expression in PCa tissues of treated-patients. These findings suggest that fulvestrant given in a neoadjuvant setting can elicit tumor-suppressing action. The question of whether fulvestrant given as neoadjuvant therapy can prevent or delay the recurrence of PCa remains an area worthy of future exploration.

In conclusion, *hsa-miR-765* is a novel fulvestrant-induced and ERβ-associated miRNA in PCa and it targets an oncogenic protein HMGA1. These findings contribute to new insights on the mode of action of fulvestrant and the potential of using miRNA for monitoring drug responsiveness as well as for future therapeutics development. They help promote the use of this antiestrogen for CRPC patients for whom no curative treatment is currently available and raise the possibility of using fulvestrant in preventing/slowing progression for locally confirmed PCa. Identification of additional miRNAs and their targets regulated by estrogens and antiestrogens may afford new opportunities for devising low toxicity therapies for PCa.

## Supporting Information

Figure S1 **Fulvestrant inhibits PC-3 cell growth and up-regulated has-miR-765 expression via an ERβ-dependent mechanism.**
(PDF)

Figure S2 **Fulvestrant significantly inhibits PC-3 cell migration and invasion.**
(PDF)

Figure S3 ***Hsa-miR-765*** **suppresses PC-3 cell growth and migration and up-regulation of HMGA1 mRNA and protein expression in the cells.**
(PDF)

Figure S4 **Effectiveness of siRNA knockdown of ERβ in DU145 cells.**
(PDF)

Figure S5 **Blocking effects of another ERβ siRNA on prostate cancer cell growth and up-regulation of hsa-miR-765 expression.**
(PDF)

Figure S6 **Expression of HMGA proteins in fulvestrant-/ethanol-treated DU145 cells transfectants with either HMGA1 expression vector or empty vector.**
(PDF)

Table S1 **Fulvestrant-regulated miRNAs.**
(PDF)

Table S2 **Primer and oligo sequences used in the study.**
(PDF)

Table S3 **Sources of the antibodies used in the study.**
(PDF)

Table S4 **Results summary of miRNA target prediction.**
(PDF)
