# Correction: Hsa-miRNA-765 as a Key Mediator for Inhibiting Growth, Migration and Invasion in Fulvestrant-Treated Prostate Cancer

**DOI:** 10.1371/journal.pone.0214184

**Published:** 2019-03-18

**Authors:** Yuet-Kin Leung, Queeny Kwan-Yi Chan, Chi-Fai Ng, Fanny Man-Ting Ma, Ho-Man Tse, Ka-Fai To, Jodi Maranchie, Shuk-Mei Ho, Kin-Mang Lau

Following the publication of [1] concerns were noted in the following figures:

- Fig 1D, the ETOH-treated panel at 0h appear similar to Fulvestrant-treated panel at 0h
- Fig 1C β-actin panel appears similar to Fig 5D β-actin panel
- Fig 3D β-actin panel appears similar to Fig 5C β-actin panel

The authors have explained that an error was made during figure preparation. The authors accidentally used the duplicated micrograph image for the Fulvestrant-treated DU145 cells at 0h to represent the ETOH-treated cells at 0h. The authors have provided a replacement image for Fig 1D ETOH-treated panel at 0h, represented by the one of the micrograph images of these cells captured during the wound healing experiment. All underlying data and images for Fig 1 have been provided in the Supporting Information file S1 Dataset.

The lysate samples of the control and Fulvestrant-treated cells used in Western blot analysis were the same for generation of data presented in both Figs 1C, 5D and Figs 3D, 5C, therefore the same image was used for β-actin expression for the purposes of presentation.

Please see the corrected Fig 1 here.

**Fig 1:**
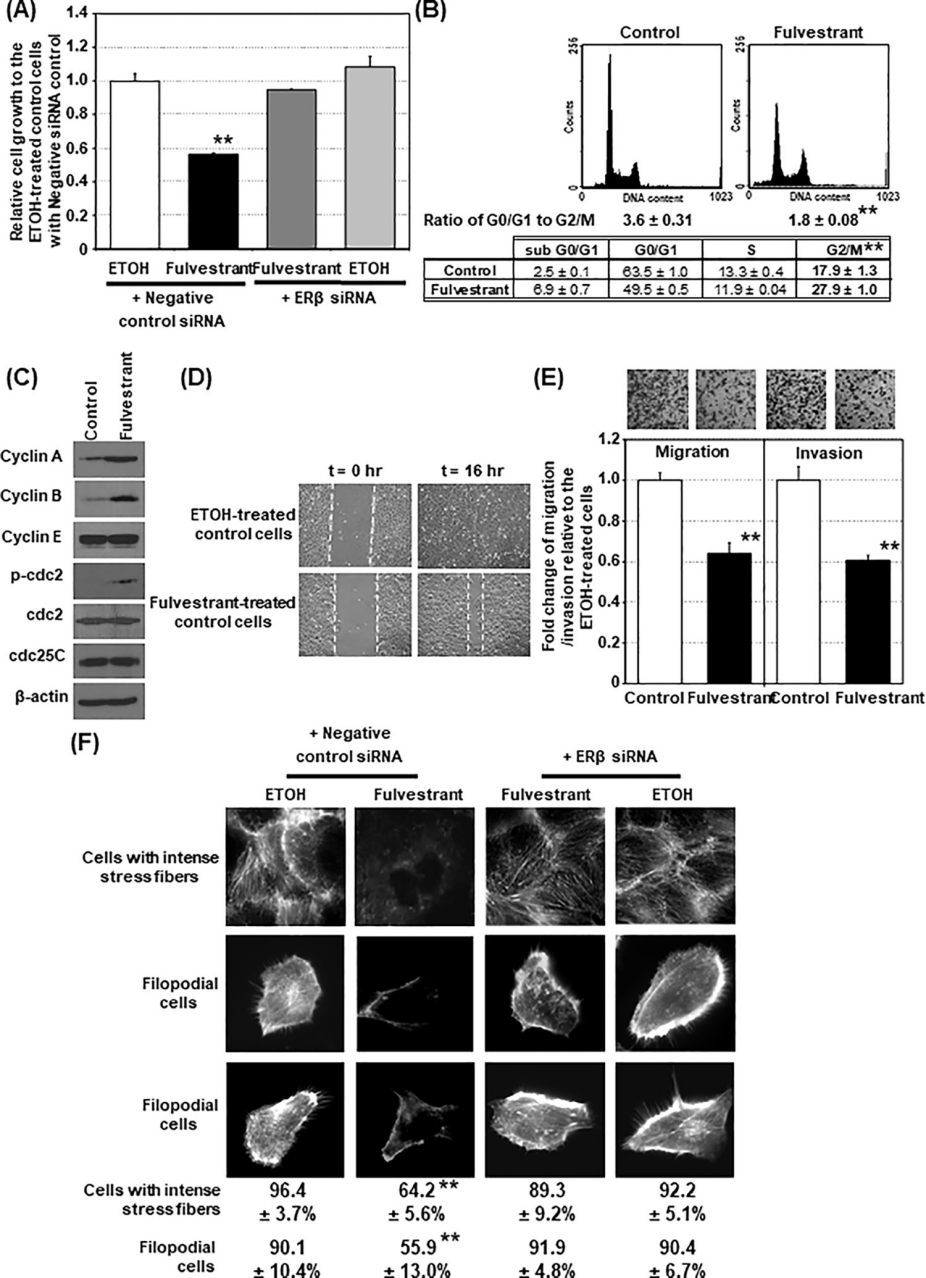
Fulvestrant inhibits DU145 cell growth, migration, and invasion. (A) Fulvestrant induces growth inhibition of DU145 cells via an ERβ-dependent mechanism. The growth of the fulvestrant-treated DU145 cells with or without ERβ siRNA knockdown for 4 days relative to the ethanol-treated control cells with negative-control siRNA are presented and compared (n = 8). ERβ expression was also knocked down by another siRNA (siRNA#2) and the similar results were obtained (S5 Fig). (B) Fulvestrant induces DU145 cell-cycle arrest at G2/M phase. Representative DNA histograms of 48hrs fulvestrant -or ethanol- (control) treated cells and percentage distributions of the cells at G0/G1 and G2/M phases (n = 3) are presented and compared. (C) Fulvestrant induces expression of G2/M markers. DU145 cells were treated with fulvestrant or ethanol for 2 days (control) and cell cycle markers were determined by Western blot analysis. Two independent experiments were performed and one representative set of data was presented. (D) Fulvestrant suppresses cell migration. A wound-healing assay was performed on the fulvestrant- and ethanol (EtOH)-treated DU145 cells (n = 3). Representative micrographs of the fulvestrant- and ethanol-treated cell cultures with scratches at 0 h and after 16 h are shown. The wound is marked by dotted lines. (E) Fulvestrant inhibits transwell migration (left panel) and invasion (right panel) in DU145 cells (n = 3) after 5 hrs of fulvestrant treatment. (F) Reductions of filopodial cells and cells with intense stress fibers by fulvestrant (treated with 48 hrs) via an ERβ-dependent mechanism. Representative micrographs and the percentages of the cells with intense stress fibers and the filopodial cells (n = 3) are presented. Student t-test was performed to determine significance with a cutoff p-value of 0.05. ** p<0.01; bars = S.D.

## Supporting information

S1 Dataset All underlying data and images for Fig 1. (ZIP)
