# Supplementary material for: Hsa-miRNA-765 as a Key Mediator for Inhibiting Growth, Migration and Invasion in Fulvestrant-Treated Prostate Cancer
Source: PLoS One. 2014 May 16;9(5):e98037. doi: 10.1371/journal.pone.0098037 (PMC4024001; doi:10.1371/journal.pone.0098037)
Supplement: Figure S1 — Fulvestrant inhibits PC-3 cell growth and up-regulated has-miR-765 expression via an ERβ-dependent mechanism. (PDF) [file pone.0098037.s001.pdf]

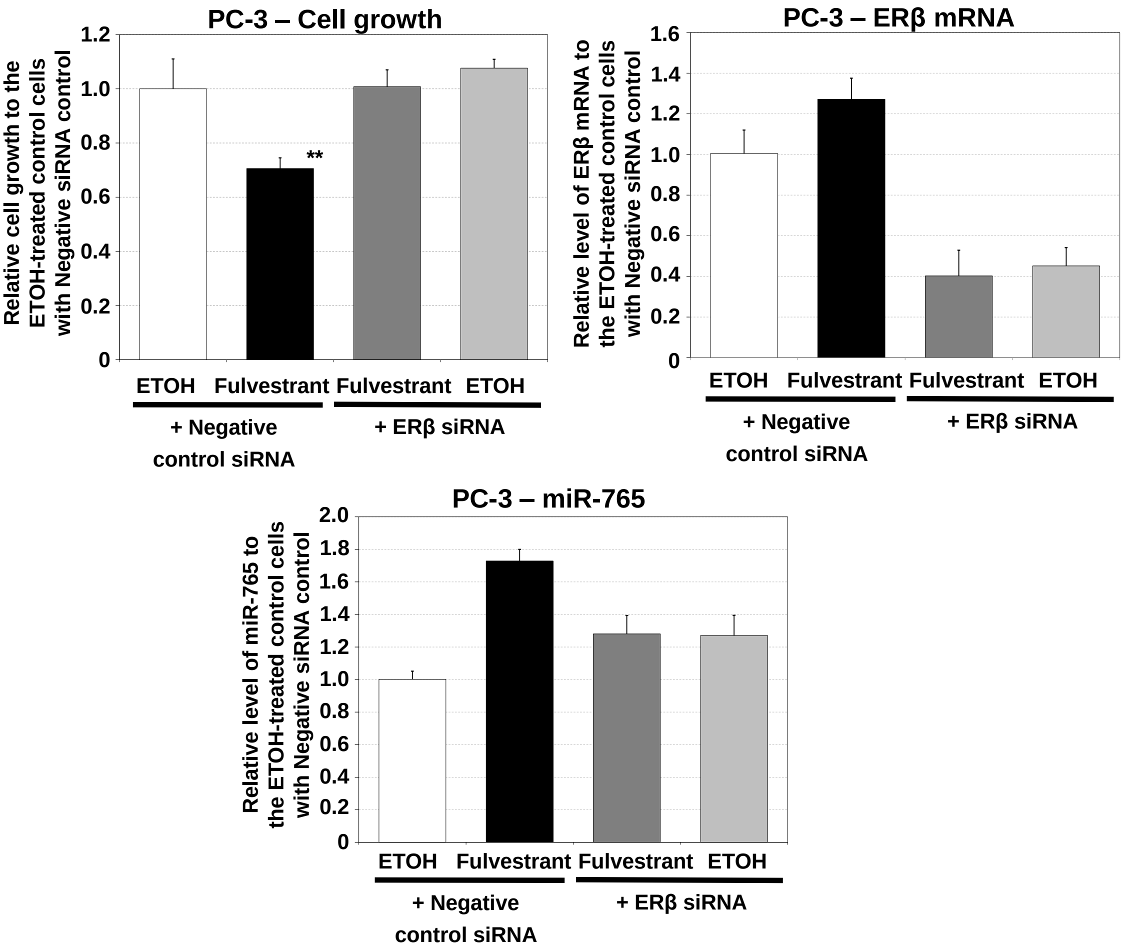


**Figure S1.** Fulvestrant inhibits PC-3 cell growth and up-regulated has-miR-765 expression via an ERβ-dependent mechanism. (A) Growth of the fulvestrant-treated PC-3 cells with or without ERβ siRNA knockdown for 4 days relative to the ethanol-treated control cells with negative-control siRNA are presented and compared (n=8) (Left). To determine the effectiveness of siRNA knockdown of ERβ, the levels of ERβ mRNA in the cells were quantified by real-time RT-PCR analysis. Results indicated the ERβ siRNA effectively reduced the expression of ERβ in the treated cells (Right). ERβ expression was also knocked down by another siRNA (siRNA#2) and the similar results were obtained (Figure S5). (B) *Hsa-miR-765* is induced by fulvestrant in PC-3 cells. The hsa-miR-765 in the fulvestrant- and ethanol-treated control PC-3 cells was quantified by miRNA qRT-PCR analysis. Relative fold changes between the expression of *hsa-miR-765* in the fulvestrant-treated and control cells are presented. Columns=means; bars =S.D.; n=3.
