# Supplementary material for: Hsa-miRNA-765 as a Key Mediator for Inhibiting Growth, Migration and Invasion in Fulvestrant-Treated Prostate Cancer
Source: PLoS One. 2014 May 16;9(5):e98037. doi: 10.1371/journal.pone.0098037 (PMC4024001; doi:10.1371/journal.pone.0098037)
Supplement: Figure S2 — Fulvestrant significantly inhibits PC-3 cell migration and invasion. (PDF) [file pone.0098037.s002.pdf]

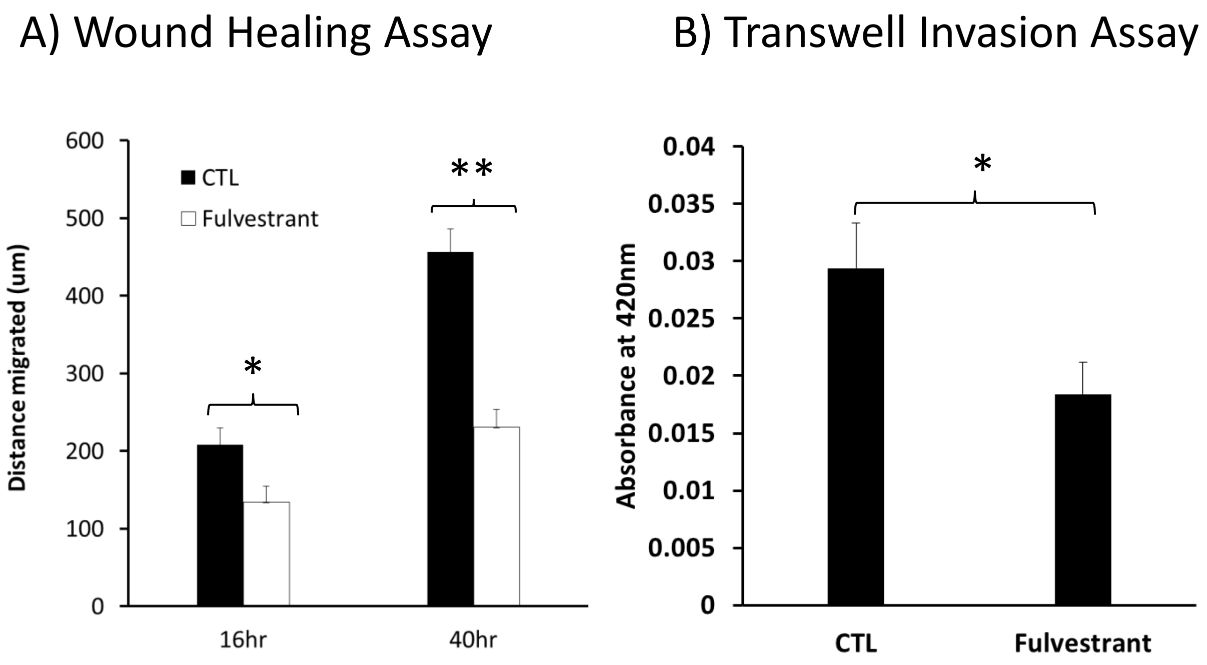

Figure S2. Fulvestrant significantly inhibits PC-3 cell migration and invasion. (A) Fulvestrant suppresses PC-3 cell migration. A wound-healing assay was performed on the fulvestrant- and vehicle-treated PC-3 cells (n=3). Migration distance of the fulvestrant- and vehicle-treated (24 hr treatment) cell cultures with scratches at 0 h and after 16 h and 40 hr was measured. (B) Fulvestrant inhibits transwell invasion in PC-3 cells (n=5) after 24 hr of fulvestrant treatment. Student t-test was performed to determine significance between ICI and CTL group with a cutoff p value of 0.05. ** $p<0.01$; bars = S.D.
