# Supplementary material for: Hsa-miRNA-765 as a Key Mediator for Inhibiting Growth, Migration and Invasion in Fulvestrant-Treated Prostate Cancer
Source: PLoS One. 2014 May 16;9(5):e98037. doi: 10.1371/journal.pone.0098037 (PMC4024001; doi:10.1371/journal.pone.0098037)
Supplement: Figure S3 — Hsa-miR-765 suppresses PC-3 cell growth and migration and up-regulation of HMGA1 mRNA and protein expression in the cells. (PDF) [file pone.0098037.s003.pdf]

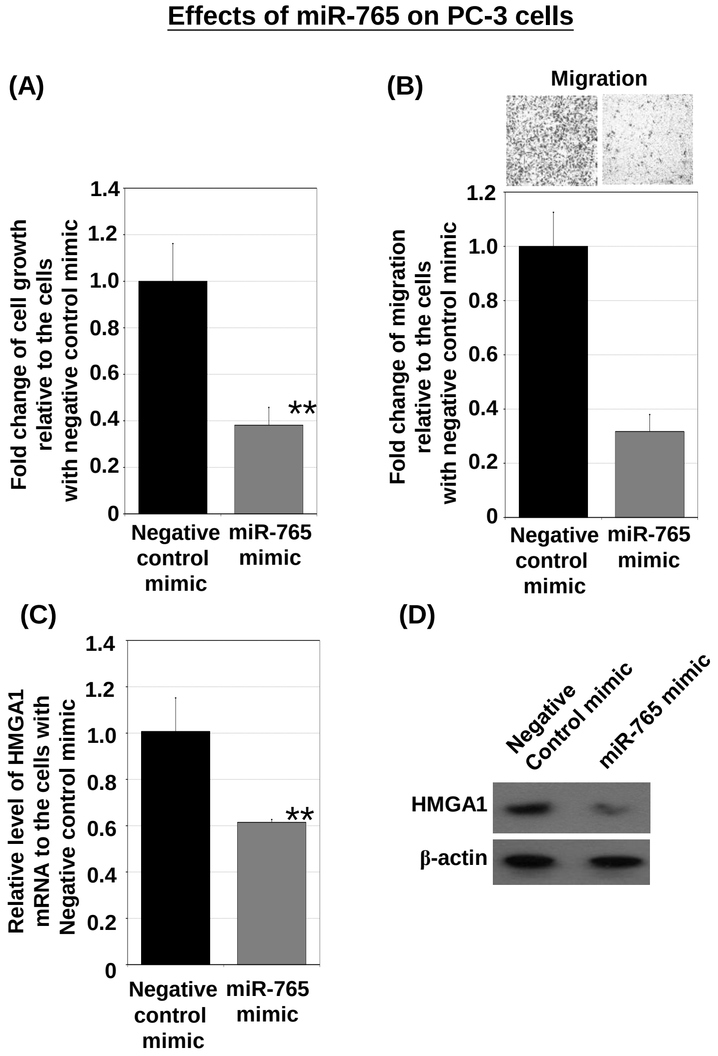

Figure S3. *Hsa-miR-765* suppresses PC-3 cell growth and migration and up-regulation of HMGA1 mRNA and protein expression in the cells. (A) *Hsa-miR-765* mimic reduces PC-3 cell growth. MTS assay was performed on the cells treated with *hsa-miR-765* mimic or negative-control mimic or transfection control for 4 days (n=8). (B) *Hsa-miR-765* mimic suppresses DU145 cell migration as shown in transwell migration assay. Representative micrographs of the cells after transwell migration (top) are presented. Fold changes of migration (bottom left) and invasion (bottom right) of PC-3 cells with either *hsa-miR-765* mimic or negative-control mimic relative to the control cells with negative-control mimic are presented (n=3). (C and D) *Hsa-miR-765* mimic reduced HMGA1 protein expression in PC-3 cells. mRNA and protein levels of HMGA1 in the *hsa-miR-765* mimic- and negative-control mimic-treated cells were determined by real-time RT-PCR analysis (C) and Western blot analysis (D), respectively. Columns=means; bar=S.D.; n=3.
