# Supplementary material for: Hsa-miRNA-765 as a Key Mediator for Inhibiting Growth, Migration and Invasion in Fulvestrant-Treated Prostate Cancer
Source: PLoS One. 2014 May 16;9(5):e98037. doi: 10.1371/journal.pone.0098037 (PMC4024001; doi:10.1371/journal.pone.0098037)
Supplement: Figure S4 — Effectiveness of siRNA knockdown of ERβ in DU145 cells. (PDF) [file pone.0098037.s004.pdf]

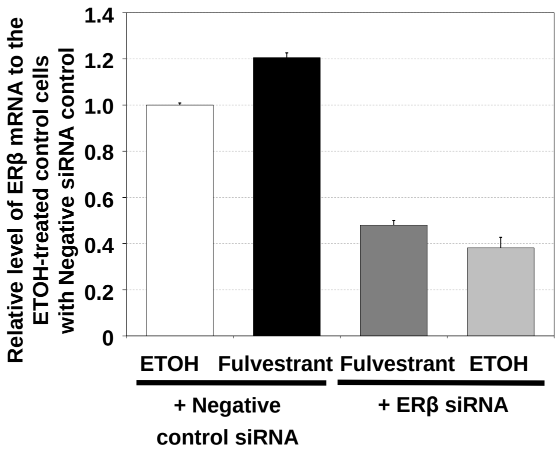

Figure S4. Effectiveness of siRNA knockdown of ERβ in DU145 cells. DU145 cells were treated with fulvestrant or ethanol (Control) in the presence of ERβ siRNA or negative-control siRNA for 4 days. The levels of ERβ in the cells were quantified by real-time RT-PCR analysis. Columns= means; bars=S.D.; n=3.
