# Supplementary material for: Hsa-miRNA-765 as a Key Mediator for Inhibiting Growth, Migration and Invasion in Fulvestrant-Treated Prostate Cancer
Source: PLoS One. 2014 May 16;9(5):e98037. doi: 10.1371/journal.pone.0098037 (PMC4024001; doi:10.1371/journal.pone.0098037)
Supplement: Figure S5 — Blocking effects of another ERβ siRNA on prostate cancer cell growth and up-regulation of hsa-miR-765 expression. (PDF) [file pone.0098037.s005.pdf]

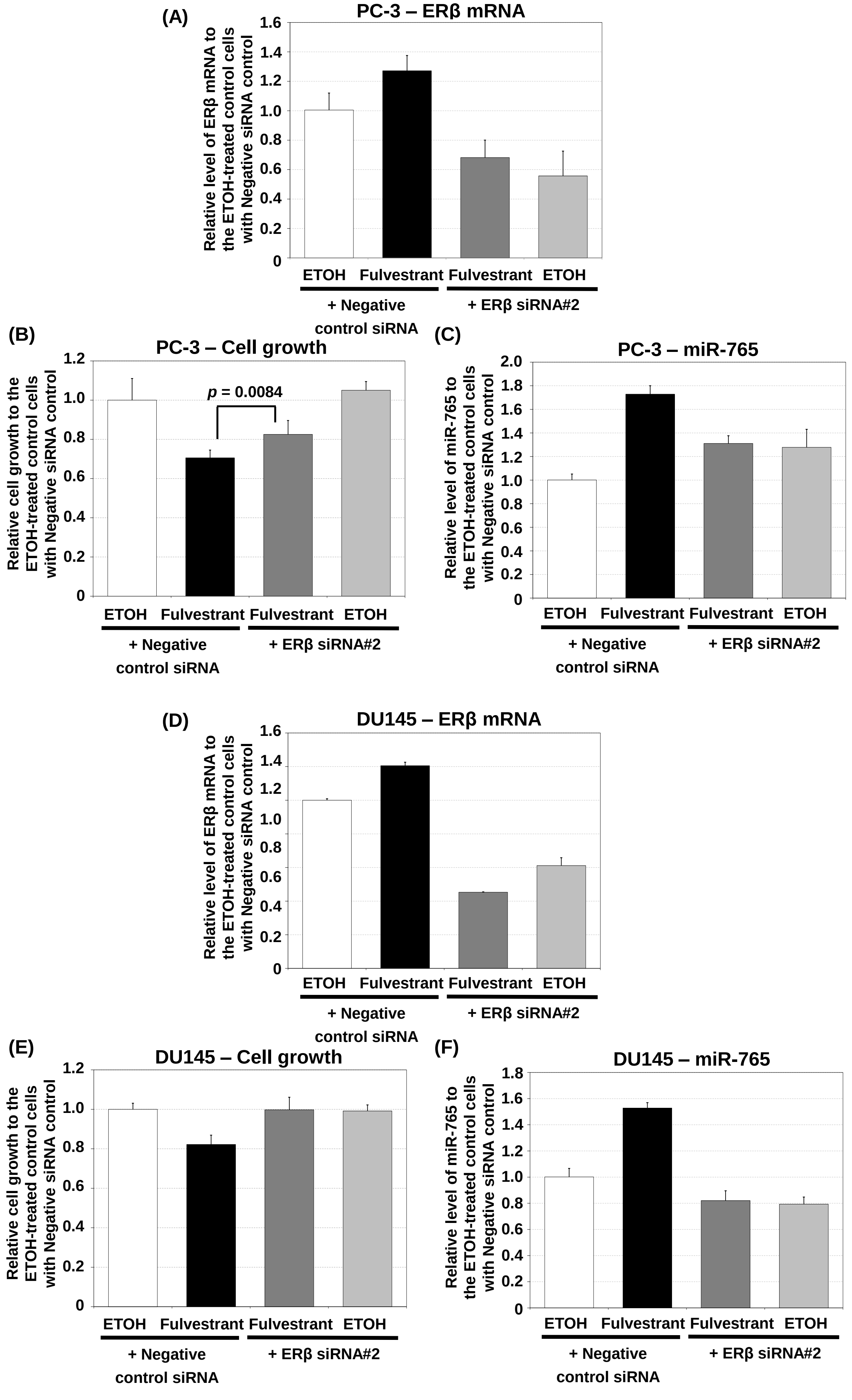
(A)
PC-3 – ERβ mRNA
Relative level of ERβ mRNA to the ETOH-treated control cells with Negative siRNA control
0
0.2
0.4
0.6
0.8
1.0
1.2
1.4
1.6
ETOH
Fulvestrant
Fulvestrant
ETOH
+ Negative control siRNA
+ ERβ siRNA#2
(B)
PC-3 – Cell growth
Relative cell growth to the ETOH-treated control cells with Negative siRNA control
0
0.2
0.4
0.6
0.8
1.0
1.2
p = 0.0084
ETOH
Fulvestrant
Fulvestrant
ETOH
+ Negative control siRNA
+ ERβ siRNA#2
(C)
PC-3 – miR-765
Relative level of miR-765 to the ETOH-treated control cells with Negative siRNA control
0
0.2
0.4
0.6
0.8
1.0
1.2
1.4
1.6
1.8
2.0
ETOH
Fulvestrant
Fulvestrant
ETOH
+ Negative control siRNA
+ ERβ siRNA#2
(D)
DU145 – ERβ mRNA
Relative level of ERβ mRNA to the ETOH-treated control cells with Negative siRNA control
0
0.2
0.4
0.6
0.8
1.0
1.2
1.4
1.6
ETOH
Fulvestrant
Fulvestrant
ETOH
+ Negative control siRNA
+ ERβ siRNA#2
(E)
DU145 – Cell growth
Relative cell growth to the ETOH-treated control cells with Negative siRNA control
0
0.2
0.4
0.6
0.8
1.0
1.2
ETOH
Fulvestrant
Fulvestrant
ETOH
+ Negative control siRNA
+ ERβ siRNA#2
(F)
DU145 – miR-765
Relative level of miR-765 to the ETOH-treated control cells with Negative siRNA control
0
0.2
0.4
0.6
0.8
1.0
1.2
1.4
1.6
1.8
ETOH
Fulvestrant
Fulvestrant
ETOH
+ Negative control siRNA
+ ERβ siRNA#2

Figure S5. Blocking effects of another ERβ siRNA on prostate cancer cell growth and up-regulation of hsa-miR-765 expression. (A and D) Effectiveness of siRNA#2 on knockdown of ERβ in PC-3 (A) and DU145 (D) cells. PC-3 and DU145 cells were treated with fulvestrant or ethanol (Control) in the presence of ERβ siRNA or negative-control siRNA for 4 days. The levels of ERβ in the cells were quantified by real-time RT-PCR analysis. Fulvestrant inhibits PC-3 and DU145 cell growth and up-regulated has-miR-765 expression via an ERβ-dependent mechanism. (B and E) Growth of the fulvestrant-treated PC-3 (B) and DU145 (E) cells with or without ERβ siRNA knockdown for 4 days relative to the ethanol-treated control cells with negative-control siRNA are presented and compared (n=8) (C and F) *Hsa-miR-765* is induced by fulvestrant in PC-3 (C) and DU145 (F) cells in an ERβ-dependent mechanism. The hsa-miR-765 in the fulvestrant- and ethanol-treated control cells was quantified by miRNA qRT-PCR analysis. Relative fold changes between the expression of *hsa-miR-765* in the fulvestrant-treated and control cells are presented. Columns=means; bars =S.D.; n=3.
