# Supplementary material for: Hsa-miRNA-765 as a Key Mediator for Inhibiting Growth, Migration and Invasion in Fulvestrant-Treated Prostate Cancer
Source: PLoS One. 2014 May 16;9(5):e98037. doi: 10.1371/journal.pone.0098037 (PMC4024001; doi:10.1371/journal.pone.0098037)
Supplement: Figure S6 — Expression of HMGA proteins in fulvestrant-/ethanol-treated DU145 cells transfectants with either HMGA1 expression vector or empty vector. (PDF) [file pone.0098037.s006.pdf]

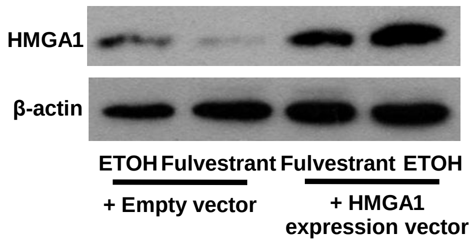

Figure S6. Expression of HMGA proteins in fulvestrant-/ethanol-treated DU145 cells transfectants with either HMGA1 expression vector or empty vector. DU145 cells were transfected with HMGA1 expression vector or empty vector (control) and treated with fulvestrant/ethanol for 4 days. The cell lysates were subjected to Western blot analysis using anti-human HMGA1 antibody to determine the levels of HMGA1 protein in the cells. Two independent experiments were performed and one representative set of data were presented.
