# Supplementary material for: Hsa-miRNA-765 as a Key Mediator for Inhibiting Growth, Migration and Invasion in Fulvestrant-Treated Prostate Cancer
Source: PLoS One. 2014 May 16;9(5):e98037. doi: 10.1371/journal.pone.0098037 (PMC4024001; doi:10.1371/journal.pone.0098037)
Supplement: Table S1 — Fulvestrant-regulated miRNAs. (PDF) [file pone.0098037.s007.pdf]

Table S1: Fulvestrant-regulated miRNAs

Upregulated miRNAs	Control	Fulvestrant-treated	Ratio	p-values
**hsa-miR-185**	319 ± 7	1228 ± 119	3.8519	0.0002
**hsa-let-7b**	2276 ± 105	3546 ± 148	1.5583	0.0003
**hsa-miR-765**	2001 ± 459	7523 ± 715	3.7599	0.0004
**hsa-let-7a**	2115 ± 249	5073 ± 734	2.3992	0.0027
**hsa-miR-601**	317 ± 206	1271 ± 215	4.0130	0.0052
**hsa-miR-768-5p**	461 ± 124	1041 ± 140	2.2605	0.0055

Normalized signal intensities of the upregulated miRNAs with a cutoff p value of 0.05
