# Supplementary material for: Hsa-miRNA-765 as a Key Mediator for Inhibiting Growth, Migration and Invasion in Fulvestrant-Treated Prostate Cancer
Source: PLoS One. 2014 May 16;9(5):e98037. doi: 10.1371/journal.pone.0098037 (PMC4024001; doi:10.1371/journal.pone.0098037)
Supplement: Table S2 — Primer and oligo sequences used in the study. (PDF) [file pone.0098037.s008.pdf]

Table S2. Primer and oligo sequences used in the study.

	Primer/Oligo sequences (5'----3')
**A. Real-time qPCR for miRNAs/small nuclear RNA**	
**hsa-miR-765**	
hsa-miR-765 qRT forward primer	TGGAGGAGAAGGAAGGTGATG
Universal qPCR reverse primer	N.A.
**RNU6**	
RNU6 qRT forward primer	CGCAAGGATGACACGCAAAT
Universal qPCR reverse primer	N.A.
**B. Real-time RT-PCR for gene mRNA**	
**HMGA1 mRNA**	
HMGA1-qRT-PCR-F	GAAAAGGACGGCACTGAGAA
HMGA1-qRT-PCR-R	CTTCCTGGAGTTGTGGTGGT
**RPS3 mRNA**	
RPS3-qRT-PCR-F	AGCCACCAGAACACAGAATG
RPS3-qRT-PCR-R	CTAGTGGCCACCTTTTCAGC
**C. Promoter analysis**	
**5' upstream sequence of hsa-miR-765**	
hsa-miR-765 promoter -F	GGAAGATCTCTGGCACAAACCAACTACCA
hsa-miR-765 promoter -R	CCCAAGCTTCTCCTCCAGAAACCCCAGTT
**D. miR-765 target sites into pMIR-REPORT vector**	
**3'UTR HMGA1**	
HMGA1 3 UTR-F	GGACTAGTCCTGCTCCTCACTGGAGGAG
HMGA1 3 UTR-R	CCCAAGCTTAGTAACTGCAAATAGGAAACC
**Complementary hsa-miR-765 recognition site**	
miR-765-Insert-sense oligo	CTAGTTTGCATCACCTTCCTTCTCCTCCACCCA
miR-765-Insert-antisense oligo	AGCTTGGGTGGAGGAGAAGGAAGGTGATGCAAA
**E. Chromatin Immunoprecipitation assay**	
ChIP-PCR-F	AAGTGATCCACCCACCTCAG
ChIP-PCR-R	CCAGTTTCTCGTAGGGCTTG
