# Supplementary material for: Hsa-miRNA-765 as a Key Mediator for Inhibiting Growth, Migration and Invasion in Fulvestrant-Treated Prostate Cancer
Source: PLoS One. 2014 May 16;9(5):e98037. doi: 10.1371/journal.pone.0098037 (PMC4024001; doi:10.1371/journal.pone.0098037)
Supplement: Table S3 — Sources of the antibodies used in the study. (PDF) [file pone.0098037.s009.pdf]

Table S3. Sources of the antibodies used in the study. The primary antibodies were diluted in 3% non-fat milk powder/5% bovine serum albumin in PBS in accordance with the recommended concentrations.

| **Protein targets of antibodies** | **Source*** |
|---|---|
| Cyclin A | Novocastra |
| Cyclin B1 | CST |
| Cyclin E1 | BD |
| cdc2 | CST |
| Phosphorylated cdc2 (Tyr15) | CST |
| cdc25C | CST |
| β-actin | Sigma |
| ERβ | SCB |
| HMGA1 | Abcam |
| goat anti-mouse IgG antibody HRP conjugated (secondary antibody) | SCB |

* Novocastra, Newcastle upon Tyne, UK; CST denotes Cell Signaling Technology, Danvers, MA; BD denotes Becton Dickinson Pharmingen, San Diego, CA; Sigma-Aldrich Corporate, St. Louis, MO; SCB denotes Santa Cruz Biotechnology, Santa Cruz, CA; Dako, Carpinteria, CA., Abcam, Cambridge, UK.
