# Supplementary material for: Hsa-miRNA-765 as a Key Mediator for Inhibiting Growth, Migration and Invasion in Fulvestrant-Treated Prostate Cancer
Source: PLoS One. 2014 May 16;9(5):e98037. doi: 10.1371/journal.pone.0098037 (PMC4024001; doi:10.1371/journal.pone.0098037)
Supplement: Table S4 — Results summary of miRNA target prediction. (PDF) [file pone.0098037.s010.pdf]

Table S4. Results summary of miRNA target prediction

	**miRNA target prediction program**	**Total no. of predicted targets**	**Scores for HMGA1**	**References**
1	TargetScan 4.2	162	Total context score = -0.11	Grimson et al 2007
2	miRanda	1667	Score = 910	John et al 2004
3	RNAhybrid	9832	N.A.	Rehmsmeier et al 2004
4	EIMMo2 (Bayesian Target Prediction algorithm)	5472	Score = 0.255723	Gaidatzis et al 2007
5	PITA algorithm	N.A.	ΔΔG score = -19.43	Kertesz et al 2007

Grimson A, Farh KK, Johnston WK, Garrett-Engele P, Lim LP, Bartel DP. MicroRNA targeting specificity in mammals: determinants beyond seed pairing. Mol Cell. 27:91-105, 2007.

John B, Enright AJ, Aravin A, Tuschl T, Sander C, Marks DS. Human MicroRNA targets. PLoS Biol. 2:e363, 2004.

Rehmsmeier M, Steffen P, Hochsmann M, Giegerich R. Fast and effective prediction of microRNA/target duplexes. RNA. 10:1507-17, 2004.

Gaidatzis D, van Nimwegen E, Hausser J, Zavolan M. Inference of miRNA targets using evolutionary conservation and pathway analysis. BMC

Kertesz M, Iovino N, Unnerstall U, Gaul U, Segal E. The role of site accessibility in microRNA target recognition. Nat Genet. 39:1278-84, 2007.

The 3'UTR of HMGA1 (nt. +8982 to +9002) is a conserved region among human, mouse, rat, and dog and is recognized by miR-765 with -22.6 kcal/mol of minimum free energy. By PITA algorithm (Kertesz et al 2007), theΔΔG score (energetic score) for the interaction between HMGA1 and miR-765 is -19.43 which is higher than the cutoff (i.e. -10). It indicates that the binding of miR-765 onto recognition site of HMGA1 is strong and the recognition is likely to be functional in endogenous miRNA expression levels. Since ΔΔG is an energetic score, the lower (more negative) its value, the stronger the binding of the microRNA to the given site is expected to be (Kertesz et al 2007). The complete lists of predicted targets of miR-765 by TargetScan 4.2, miRanda, RNAhybrid, and EIMMo2 were shown in Supplementary Tables S5-8).
