# Supplementary material for: Correction: Hsa-miRNA-765 as a Key Mediator for Inhibiting Growth, Migration and Invasion in Fulvestrant-Treated Prostate Cancer
Source: PLoS One. 2019 Mar 18;14(3):e0214184. doi: 10.1371/journal.pone.0214184 (PMC6422296; doi:10.1371/journal.pone.0214184)
Supplement: S1 Dataset — (ZIP) [file pone.0214184.s001.zip › raw data/Figure 1B raw data.pptx]

## Slide 1
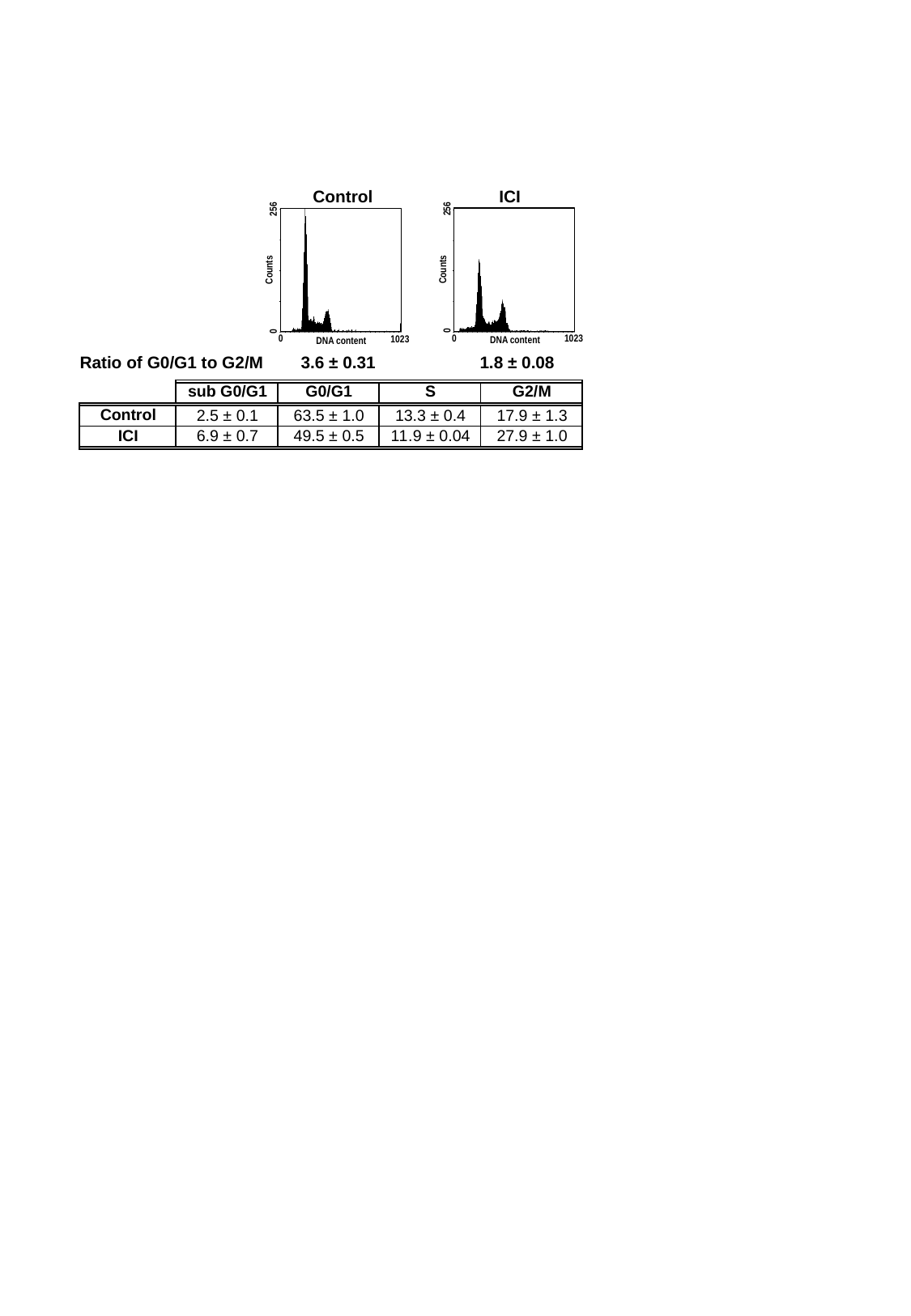

Control
256
Counts
0
0
1023
DNA content
ICI
256
Counts
0
0
1023
DNA content
Ratio of G0/G1 to G2/M
3.6 ± 0.31
1.8 ± 0.08

## Slide 2
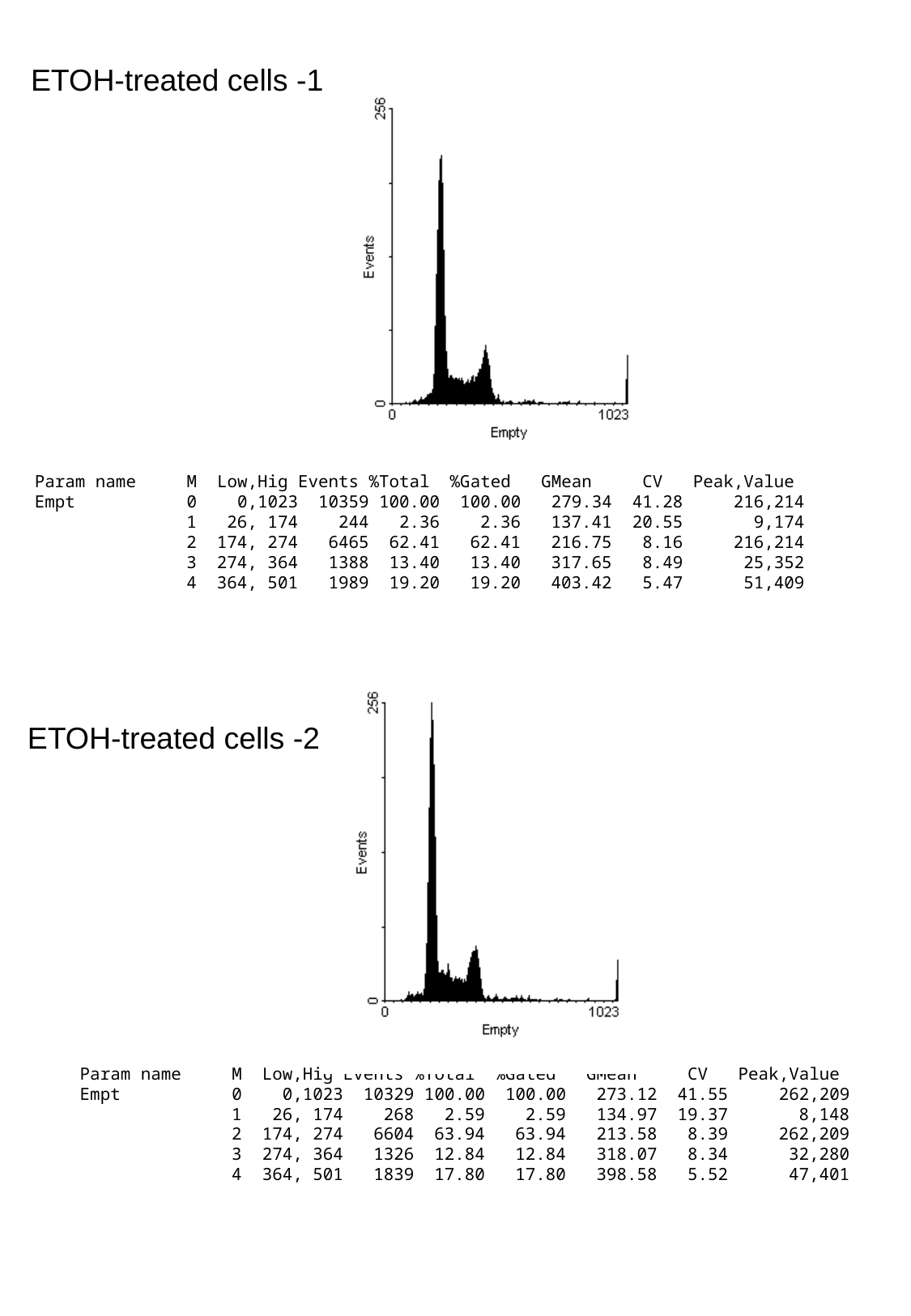

ETOH-treated cells -1
Param name M Low,Hig Events %Total %Gated GMean CV Peak,Value
Empt 0 0,1023 10359 100.00 100.00 279.34 41.28 216,214
 1 26, 174 244 2.36 2.36 137.41 20.55 9,174
 2 174, 274 6465 62.41 62.41 216.75 8.16 216,214
 3 274, 364 1388 13.40 13.40 317.65 8.49 25,352
 4 364, 501 1989 19.20 19.20 403.42 5.47 51,409
ETOH-treated cells -2
Param name M Low,Hig Events %Total %Gated GMean CV Peak,Value
Empt 0 0,1023 10329 100.00 100.00 273.12 41.55 262,209
 1 26, 174 268 2.59 2.59 134.97 19.37 8,148
 2 174, 274 6604 63.94 63.94 213.58 8.39 262,209
 3 274, 364 1326 12.84 12.84 318.07 8.34 32,280
 4 364, 501 1839 17.80 17.80 398.58 5.52 47,401

## Slide 3
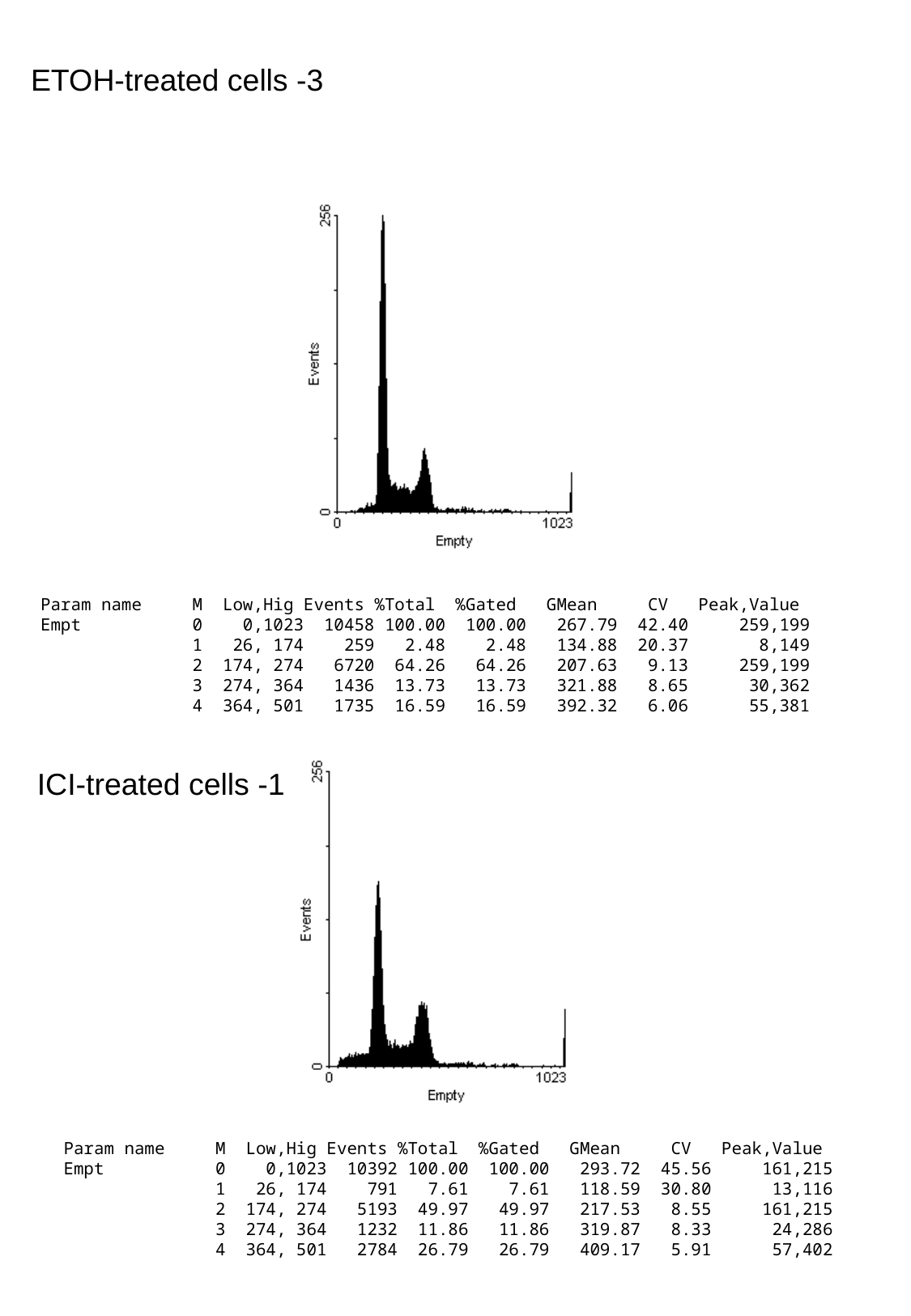

ETOH-treated cells -3
Param name M Low,Hig Events %Total %Gated GMean CV Peak,Value
Empt 0 0,1023 10458 100.00 100.00 267.79 42.40 259,199
 1 26, 174 259 2.48 2.48 134.88 20.37 8,149
 2 174, 274 6720 64.26 64.26 207.63 9.13 259,199
 3 274, 364 1436 13.73 13.73 321.88 8.65 30,362
 4 364, 501 1735 16.59 16.59 392.32 6.06 55,381
ICI-treated cells -1
Param name M Low,Hig Events %Total %Gated GMean CV Peak,Value
Empt 0 0,1023 10392 100.00 100.00 293.72 45.56 161,215
 1 26, 174 791 7.61 7.61 118.59 30.80 13,116
 2 174, 274 5193 49.97 49.97 217.53 8.55 161,215
 3 274, 364 1232 11.86 11.86 319.87 8.33 24,286
 4 364, 501 2784 26.79 26.79 409.17 5.91 57,402

## Slide 4
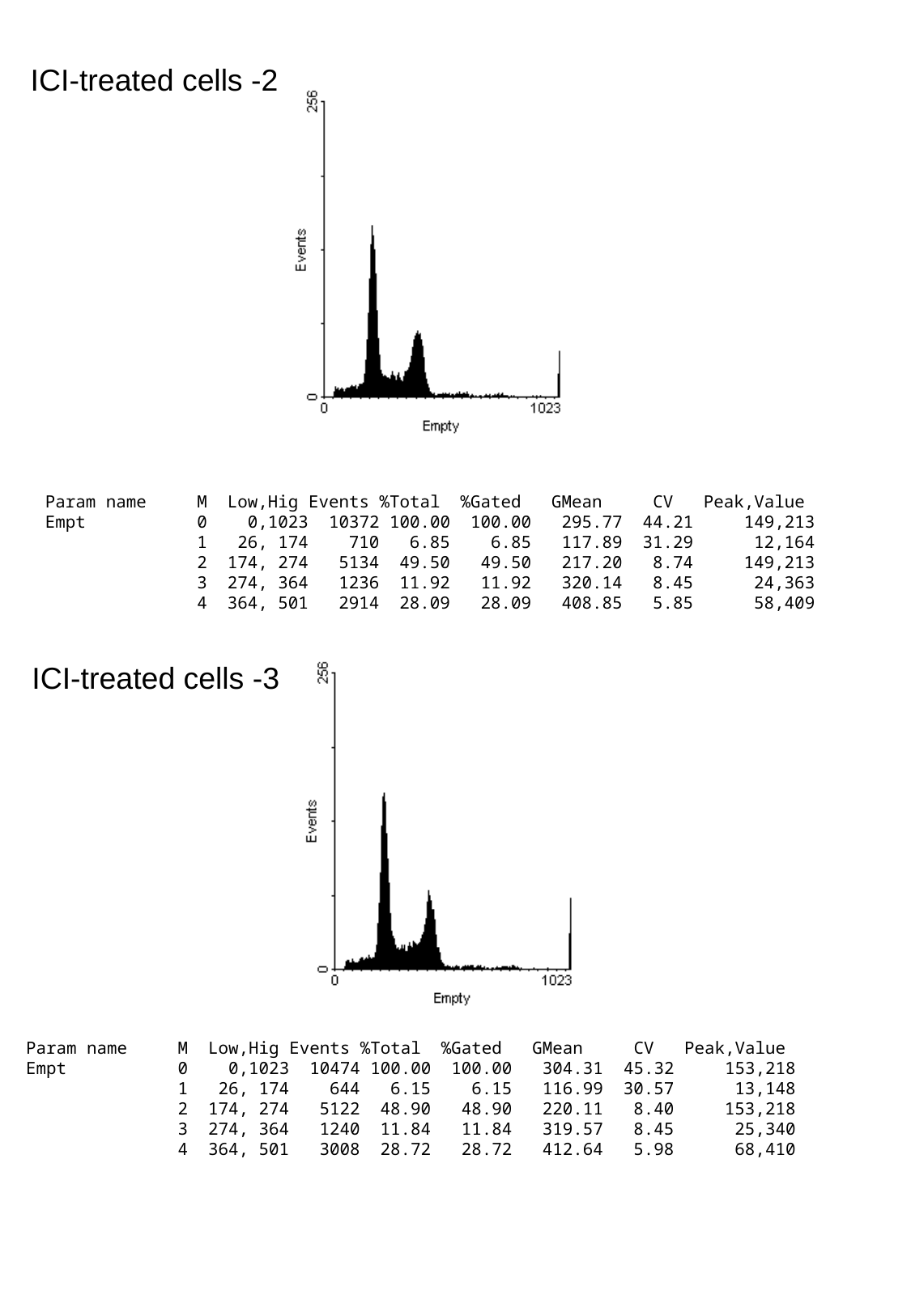

ICI-treated cells -2
Param name M Low,Hig Events %Total %Gated GMean CV Peak,Value
Empt 0 0,1023 10372 100.00 100.00 295.77 44.21 149,213
 1 26, 174 710 6.85 6.85 117.89 31.29 12,164
 2 174, 274 5134 49.50 49.50 217.20 8.74 149,213
 3 274, 364 1236 11.92 11.92 320.14 8.45 24,363
 4 364, 501 2914 28.09 28.09 408.85 5.85 58,409
ICI-treated cells -3
Param name M Low,Hig Events %Total %Gated GMean CV Peak,Value
Empt 0 0,1023 10474 100.00 100.00 304.31 45.32 153,218
 1 26, 174 644 6.15 6.15 116.99 30.57 13,148
 2 174, 274 5122 48.90 48.90 220.11 8.40 153,218
 3 274, 364 1240 11.84 11.84 319.57 8.45 25,340
 4 364, 501 3008 28.72 28.72 412.64 5.98 68,410
