# Supplementary figures and images for: Correction: Hsa-miRNA-765 as a Key Mediator for Inhibiting Growth, Migration and Invasion in Fulvestrant-Treated Prostate Cancer
Source: PLoS One. 2019 Mar 18;14(3):e0214184. doi: 10.1371/journal.pone.0214184 (PMC6422296; doi:10.1371/journal.pone.0214184)

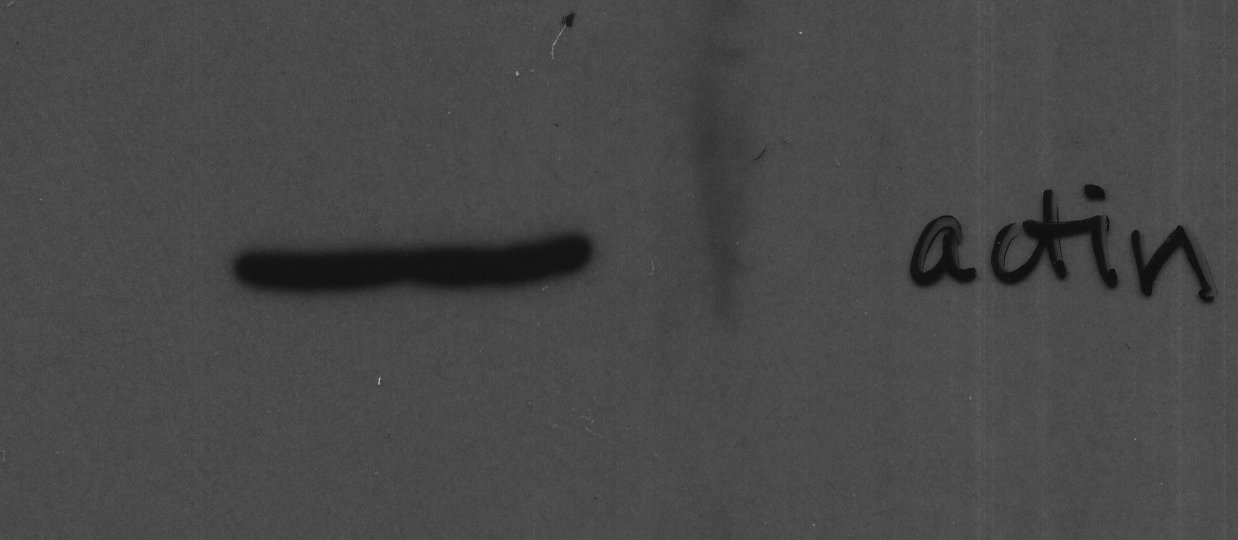

Supplement: S1 Dataset — (ZIP) [file pone.0214184.s001.zip › raw data/Figure 1C raw data/actin_DU145_EtOH-ICI_raw.jpg]

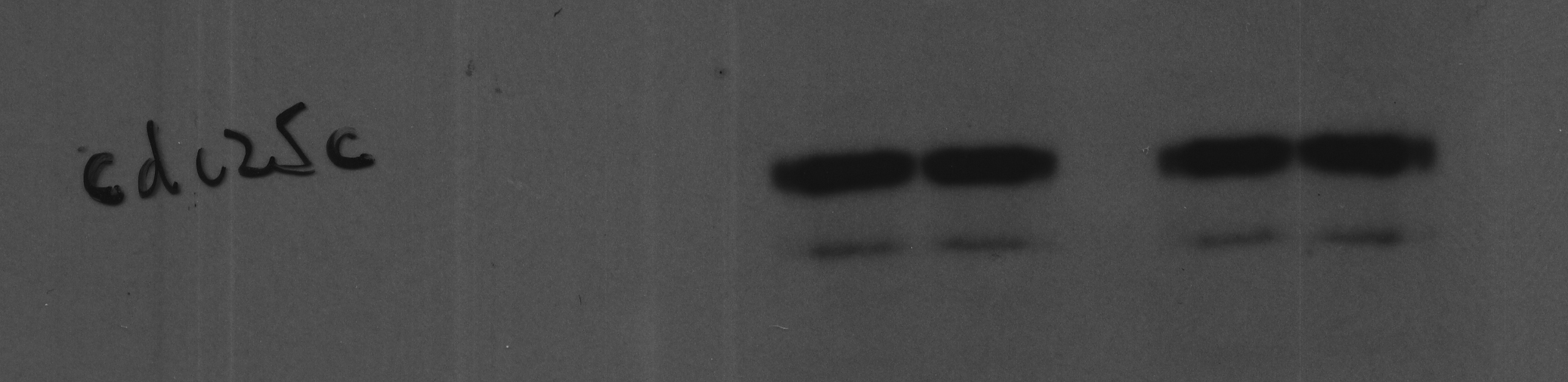

Supplement: S1 Dataset — (ZIP) [file pone.0214184.s001.zip › raw data/Figure 1C raw data/cdc25C_DU145_EtOH-ICI_raw_Right_Panel.jpg]

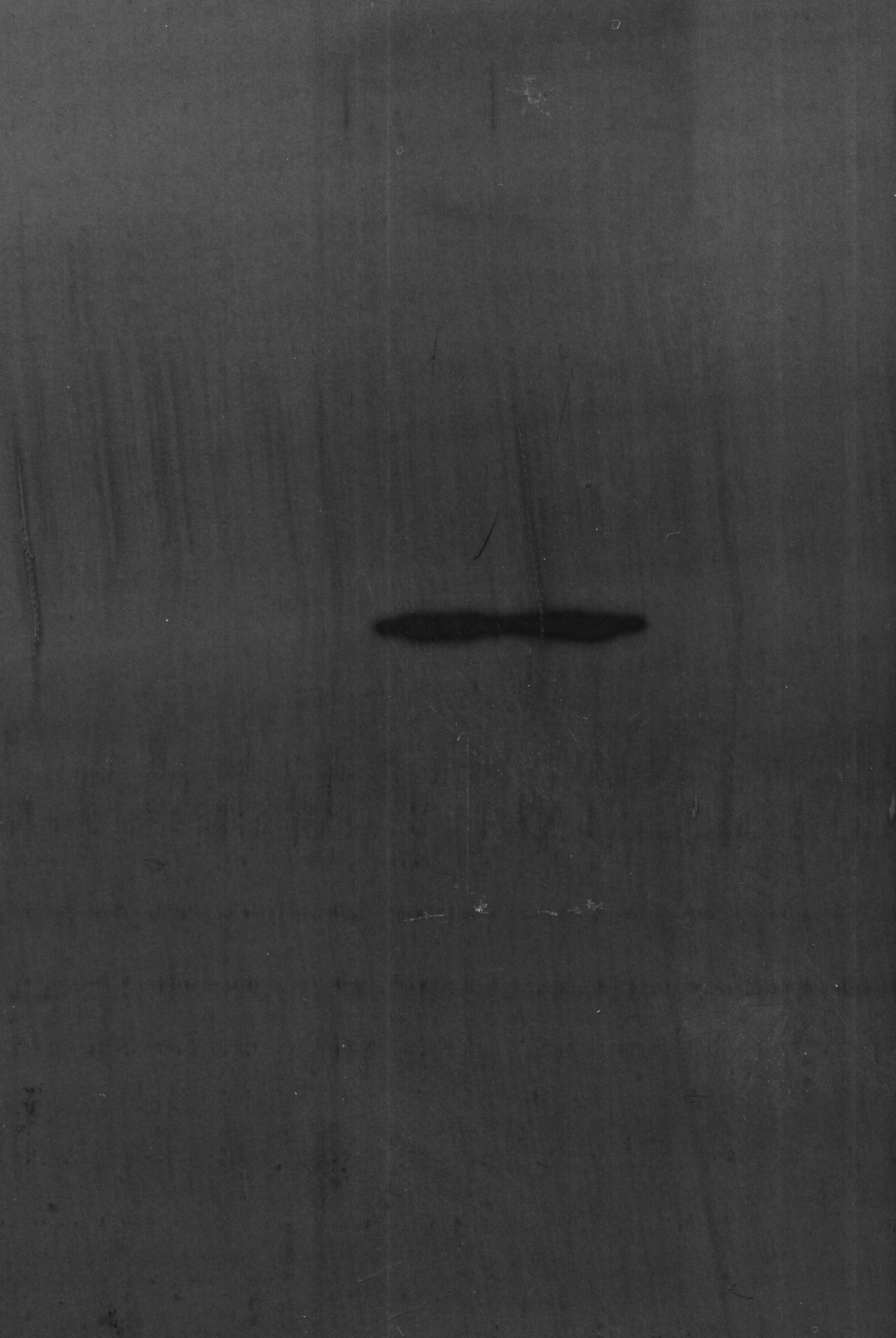

Supplement: S1 Dataset — (ZIP) [file pone.0214184.s001.zip › raw data/Figure 1C raw data/cdc2_DU145_EtOH-ICI_raw.jpg]

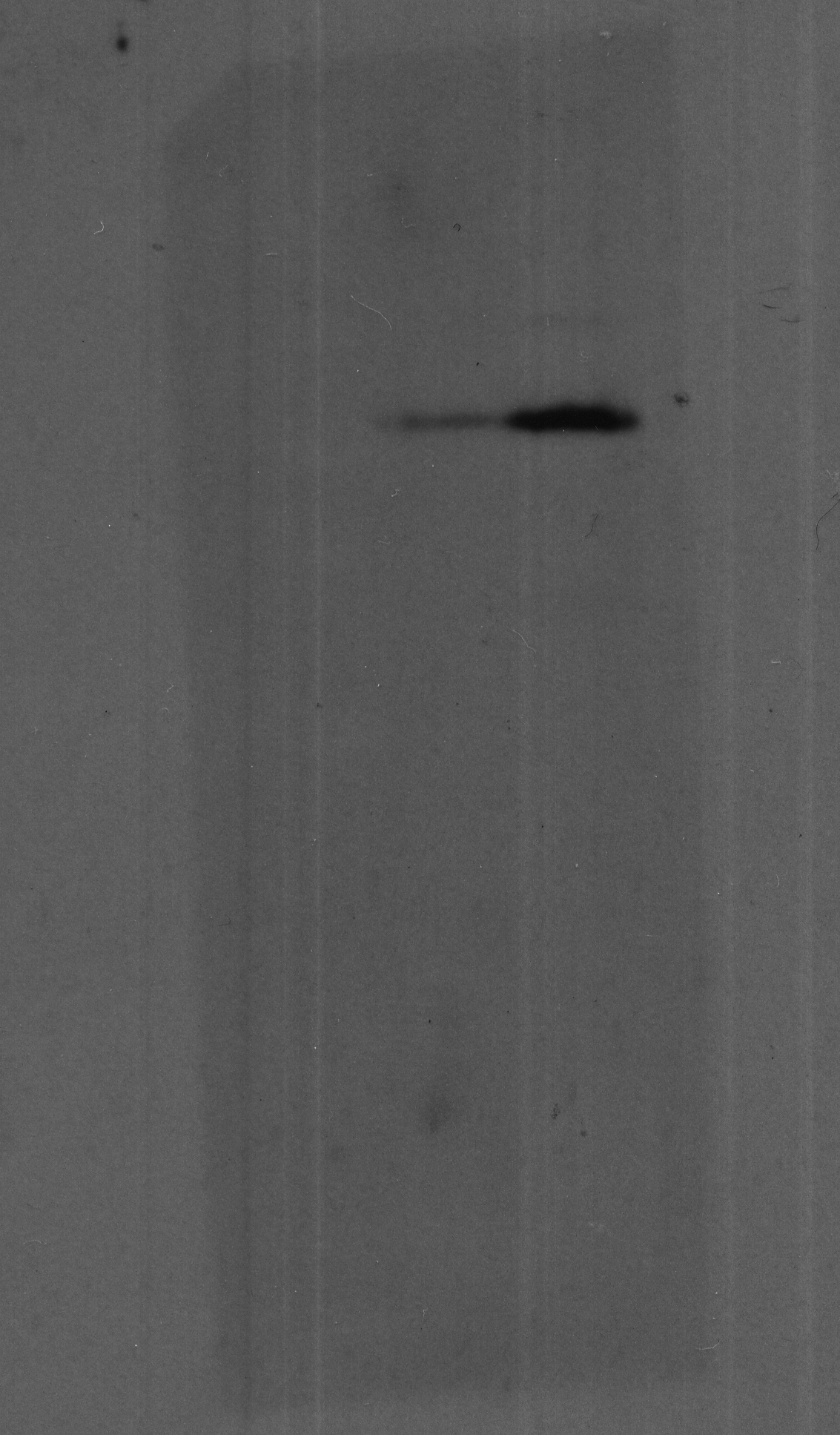

Supplement: S1 Dataset — (ZIP) [file pone.0214184.s001.zip › raw data/Figure 1C raw data/cyclin B_DU145_EtOH-ICI_raw.jpg]

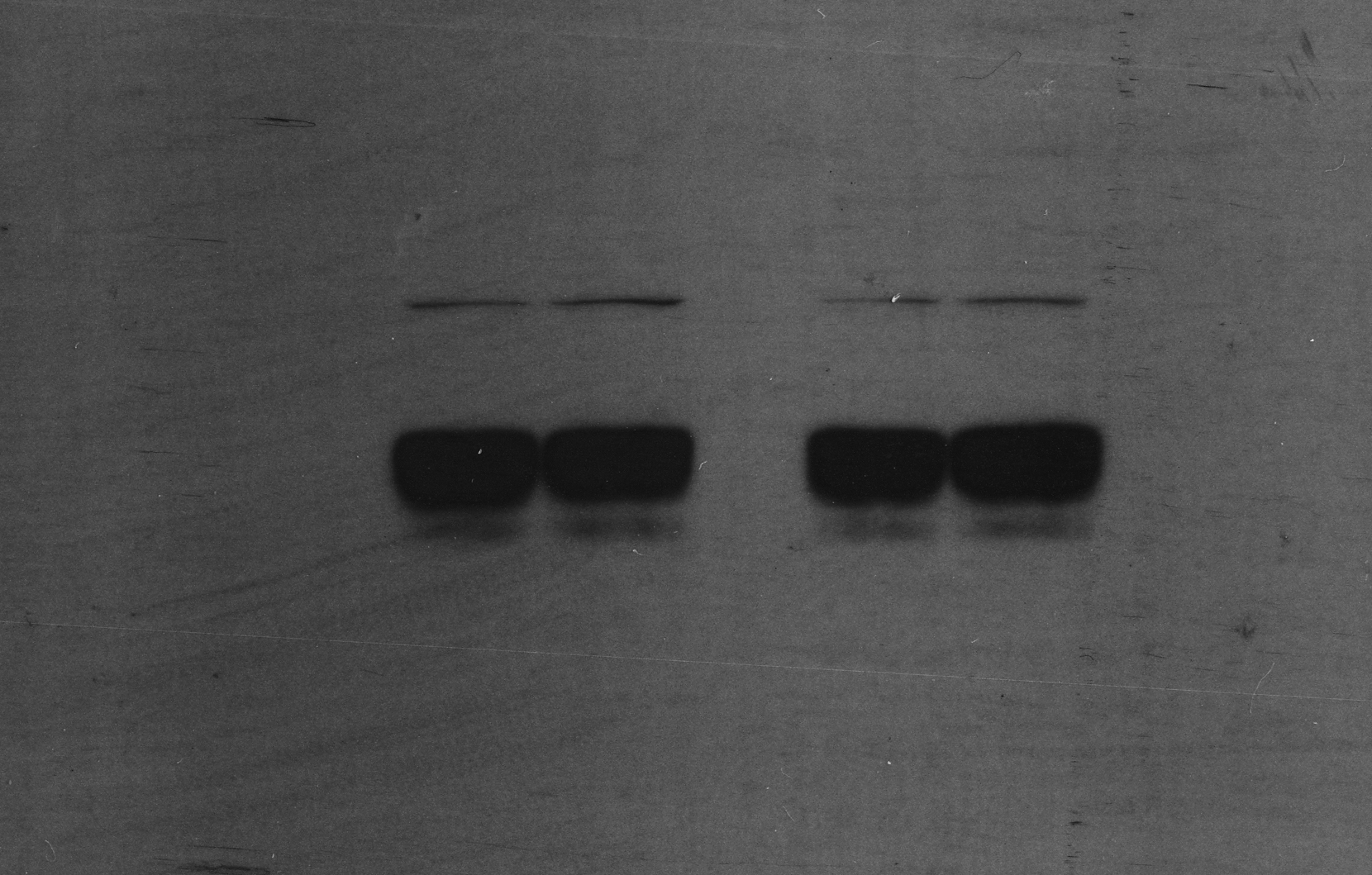

Supplement: S1 Dataset — (ZIP) [file pone.0214184.s001.zip › raw data/Figure 1C raw data/cyclin E_DU145_EtOH-ICI_raw_Right_Panel.jpg]

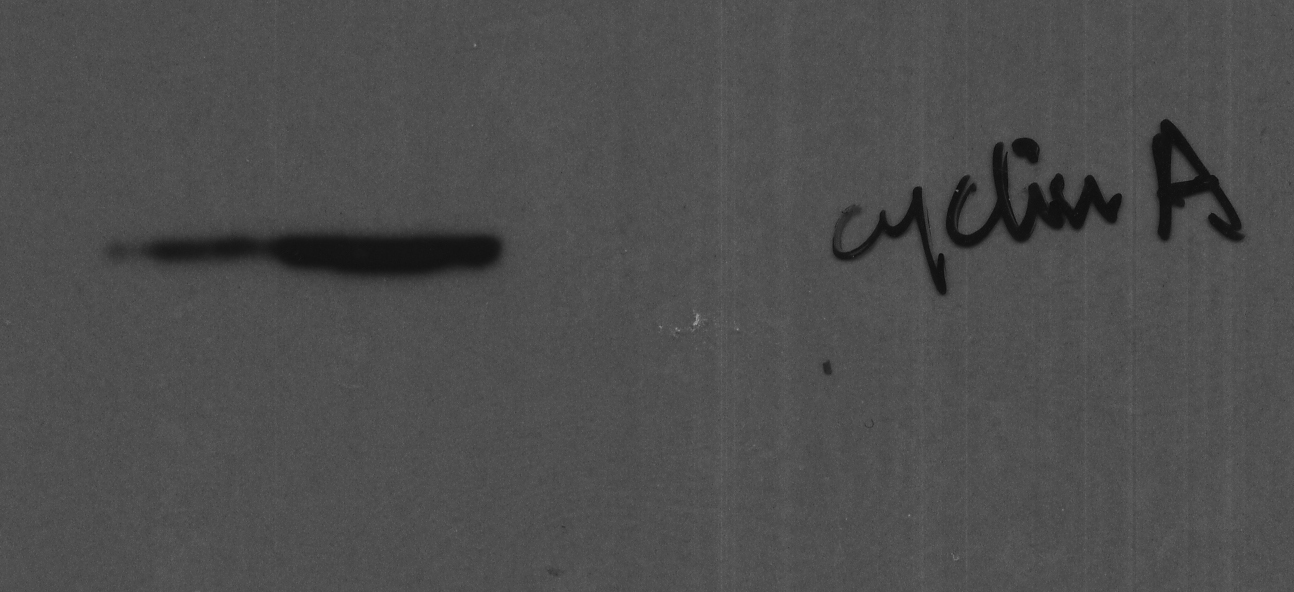

Supplement: S1 Dataset — (ZIP) [file pone.0214184.s001.zip › raw data/Figure 1C raw data/cyclinA_DU145_EtOH-ICI_raw.jpg]

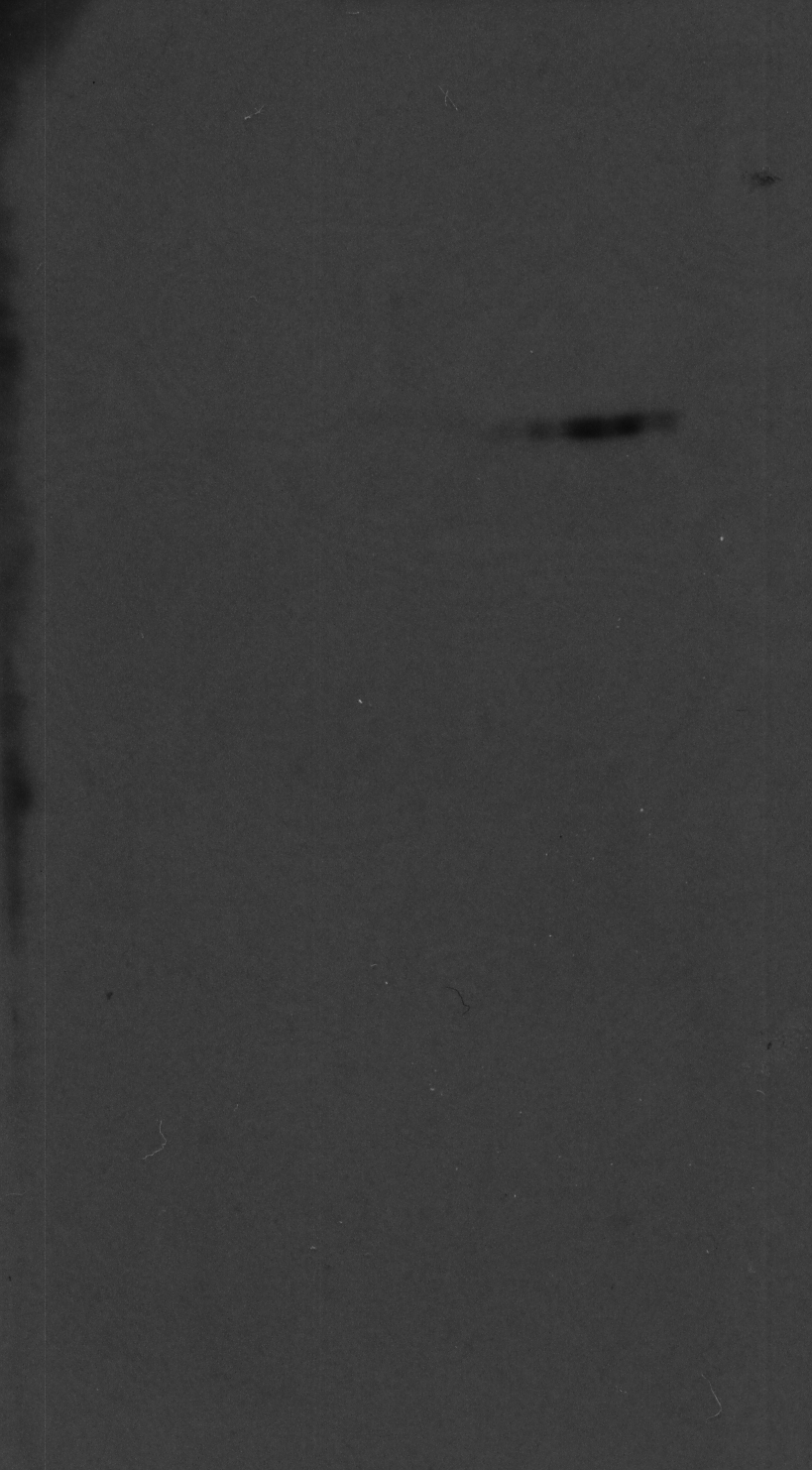

Supplement: S1 Dataset — (ZIP) [file pone.0214184.s001.zip › raw data/Figure 1C raw data/P-cdc2_DU145_EtOH-ICI_raw.jpg]

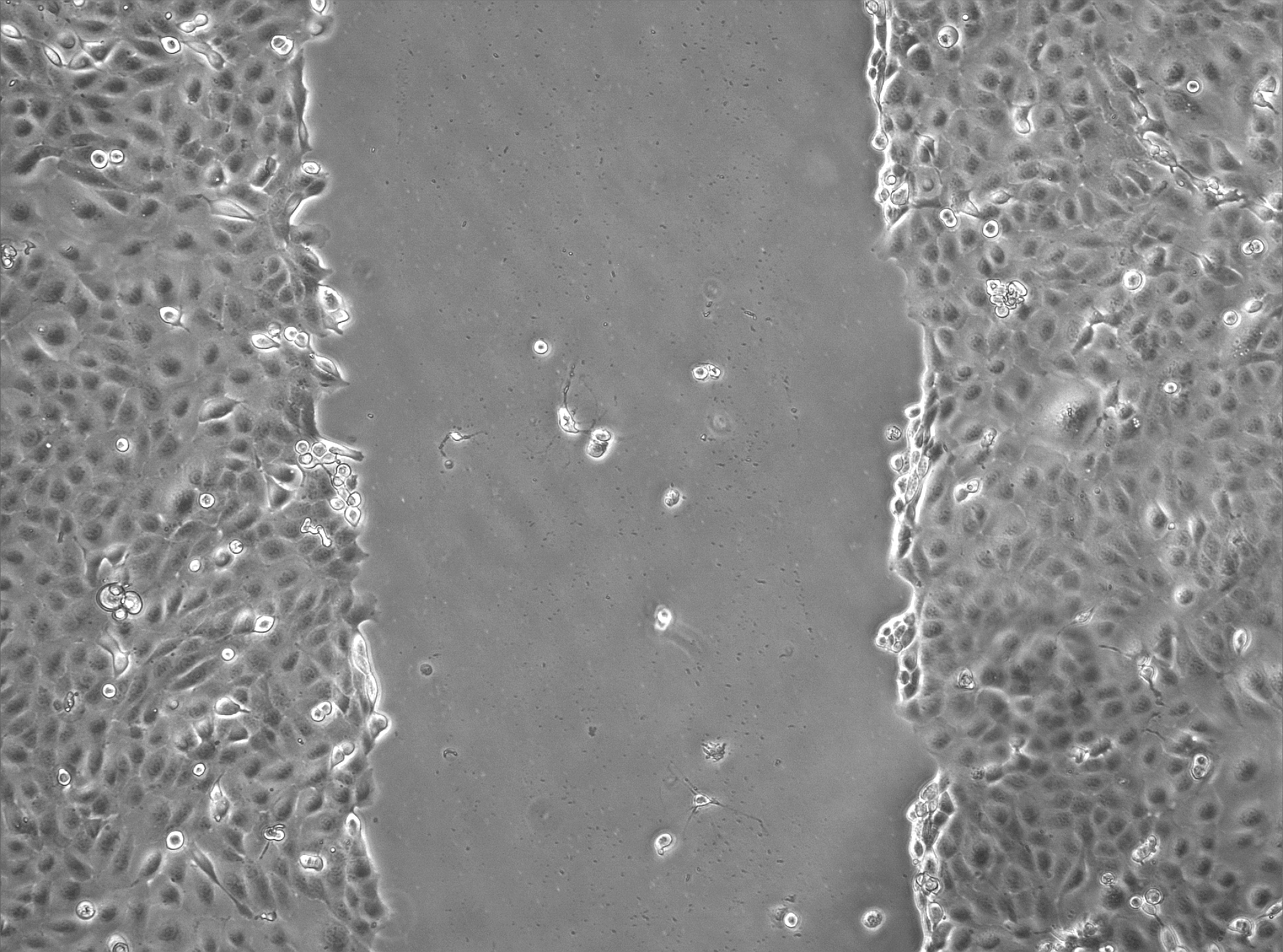

Supplement: S1 Dataset — (ZIP) [file pone.0214184.s001.zip › raw data/Figure 1D raw data/EtOH 1 position 1 t=0.jpg]

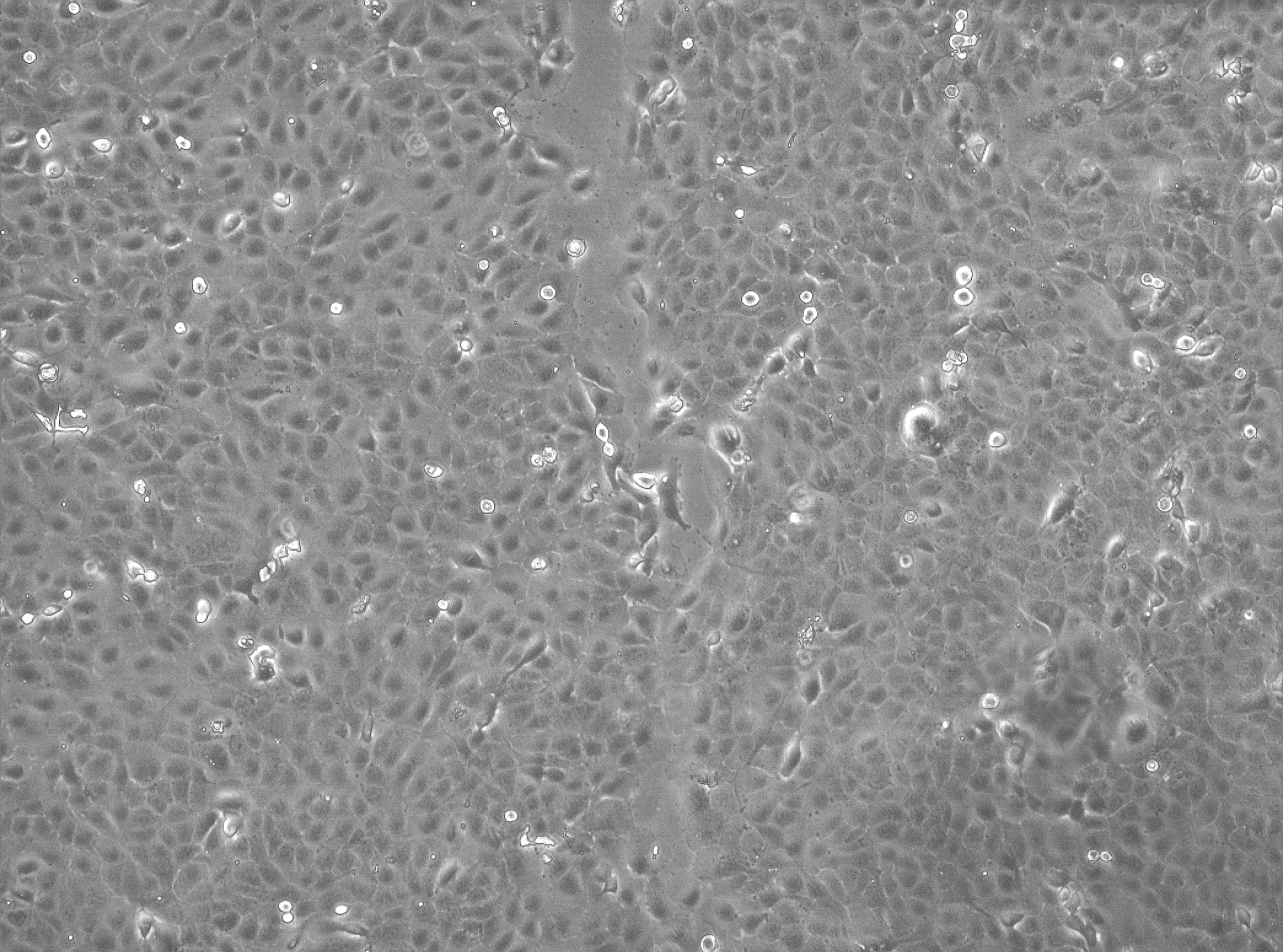

Supplement: S1 Dataset — (ZIP) [file pone.0214184.s001.zip › raw data/Figure 1D raw data/EtOH 1 position 1 t=16.jpg]

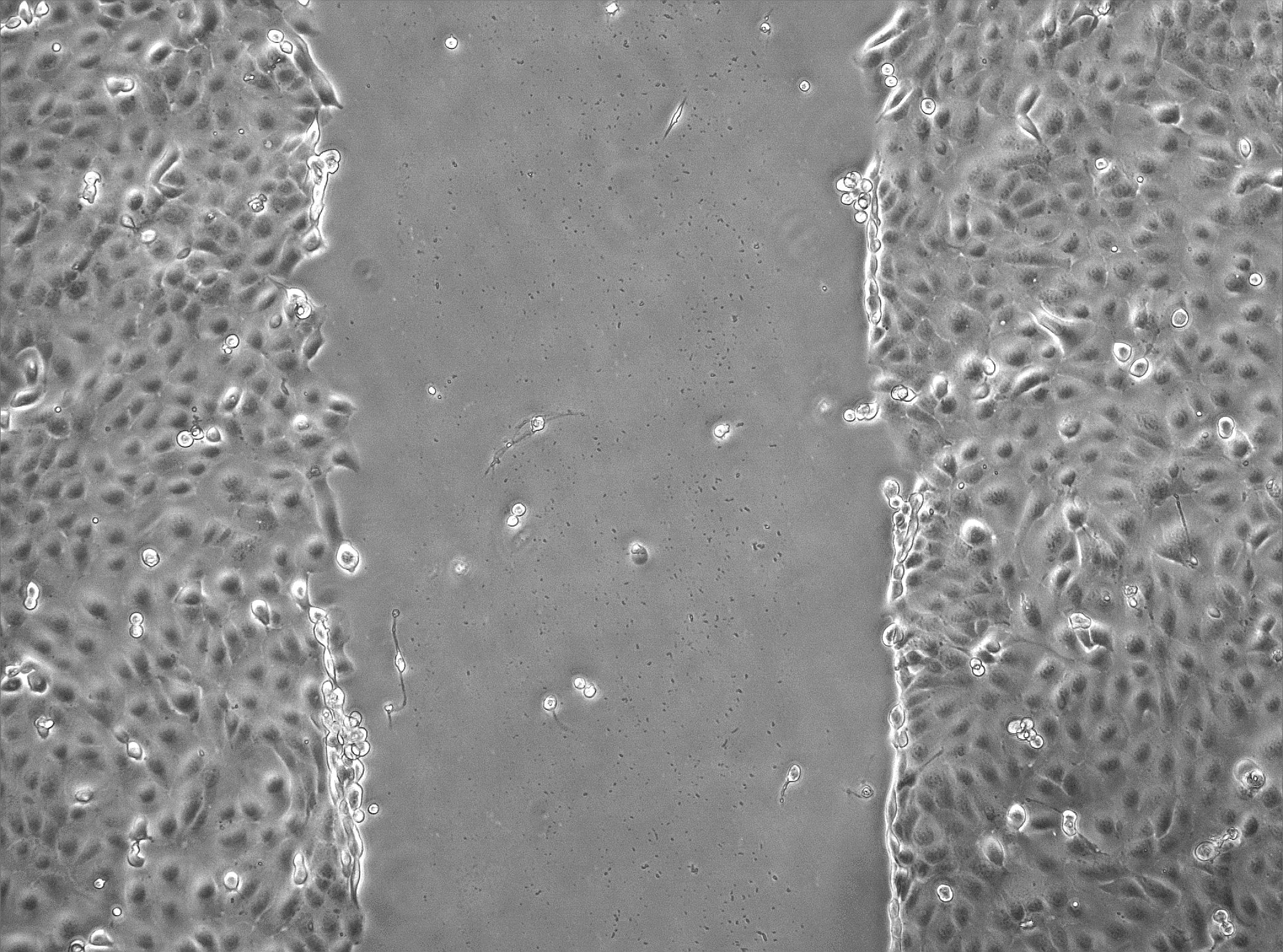

Supplement: S1 Dataset — (ZIP) [file pone.0214184.s001.zip › raw data/Figure 1D raw data/EtOH 1 position 2 t=0.jpg]

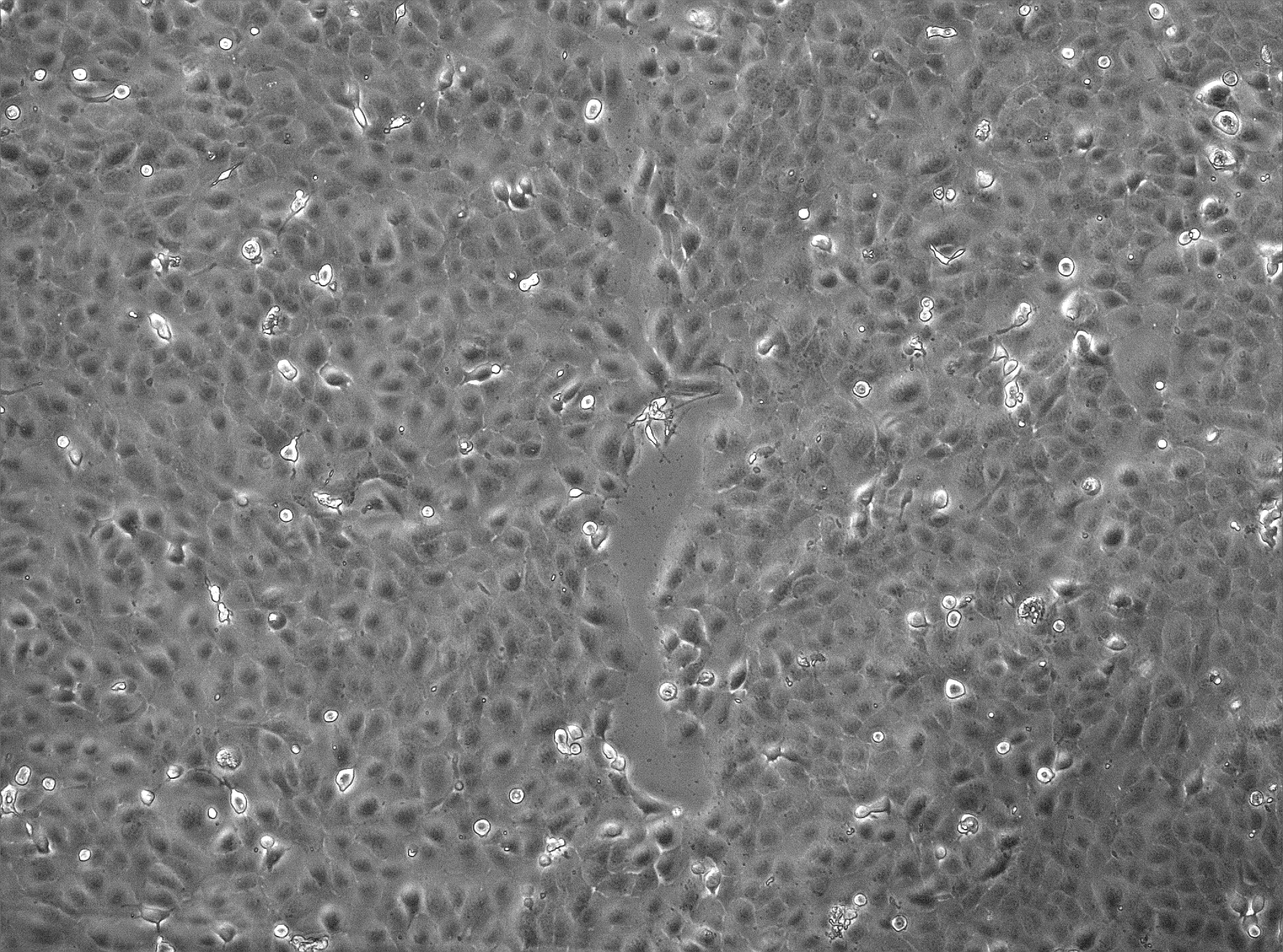

Supplement: S1 Dataset — (ZIP) [file pone.0214184.s001.zip › raw data/Figure 1D raw data/EtOH 1 position 2 t=16.jpg]

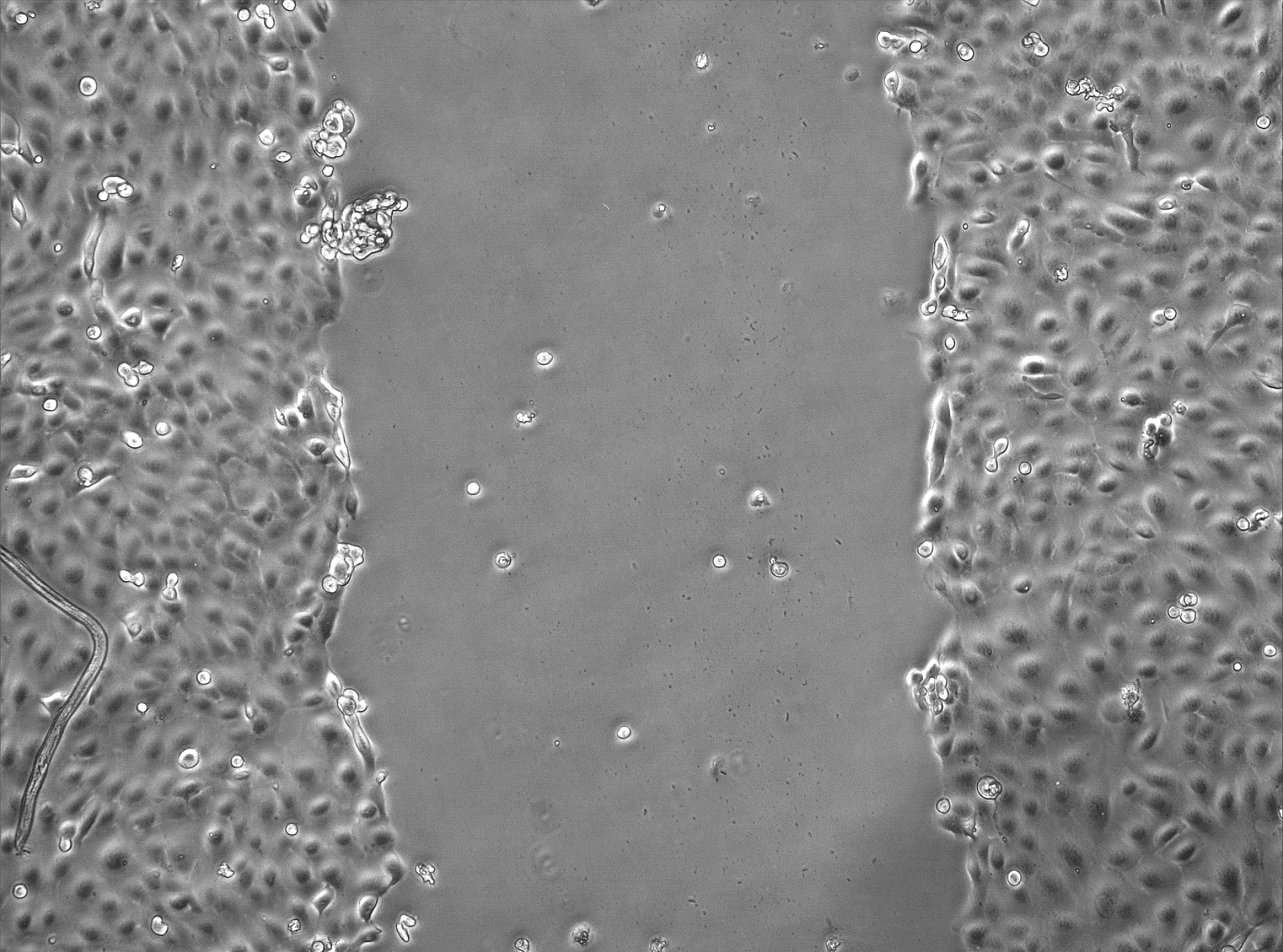

Supplement: S1 Dataset — (ZIP) [file pone.0214184.s001.zip › raw data/Figure 1D raw data/EtOH 1 position 3 t=0.jpg]

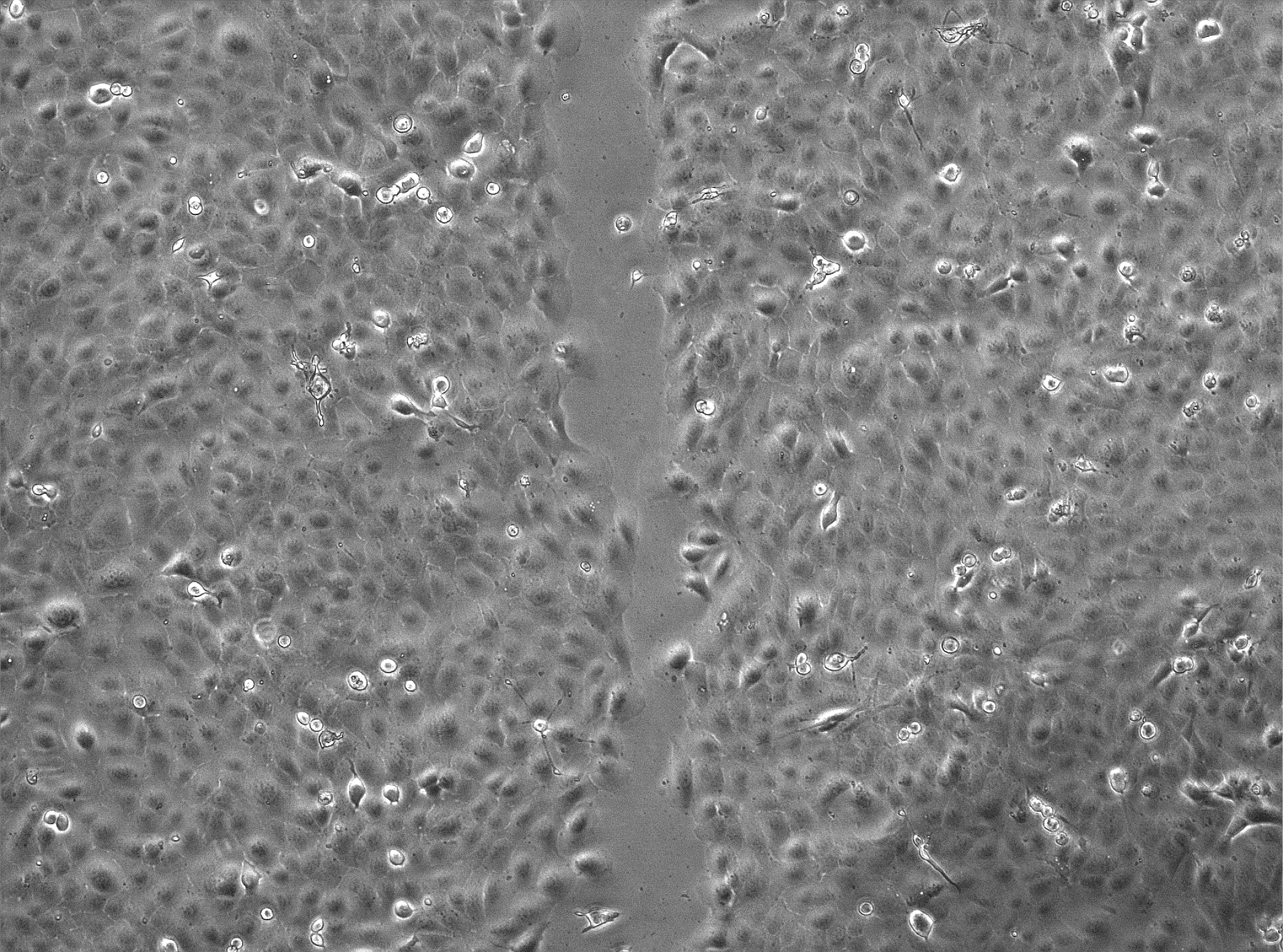

Supplement: S1 Dataset — (ZIP) [file pone.0214184.s001.zip › raw data/Figure 1D raw data/EtOH 1 position 3 t=16.jpg]

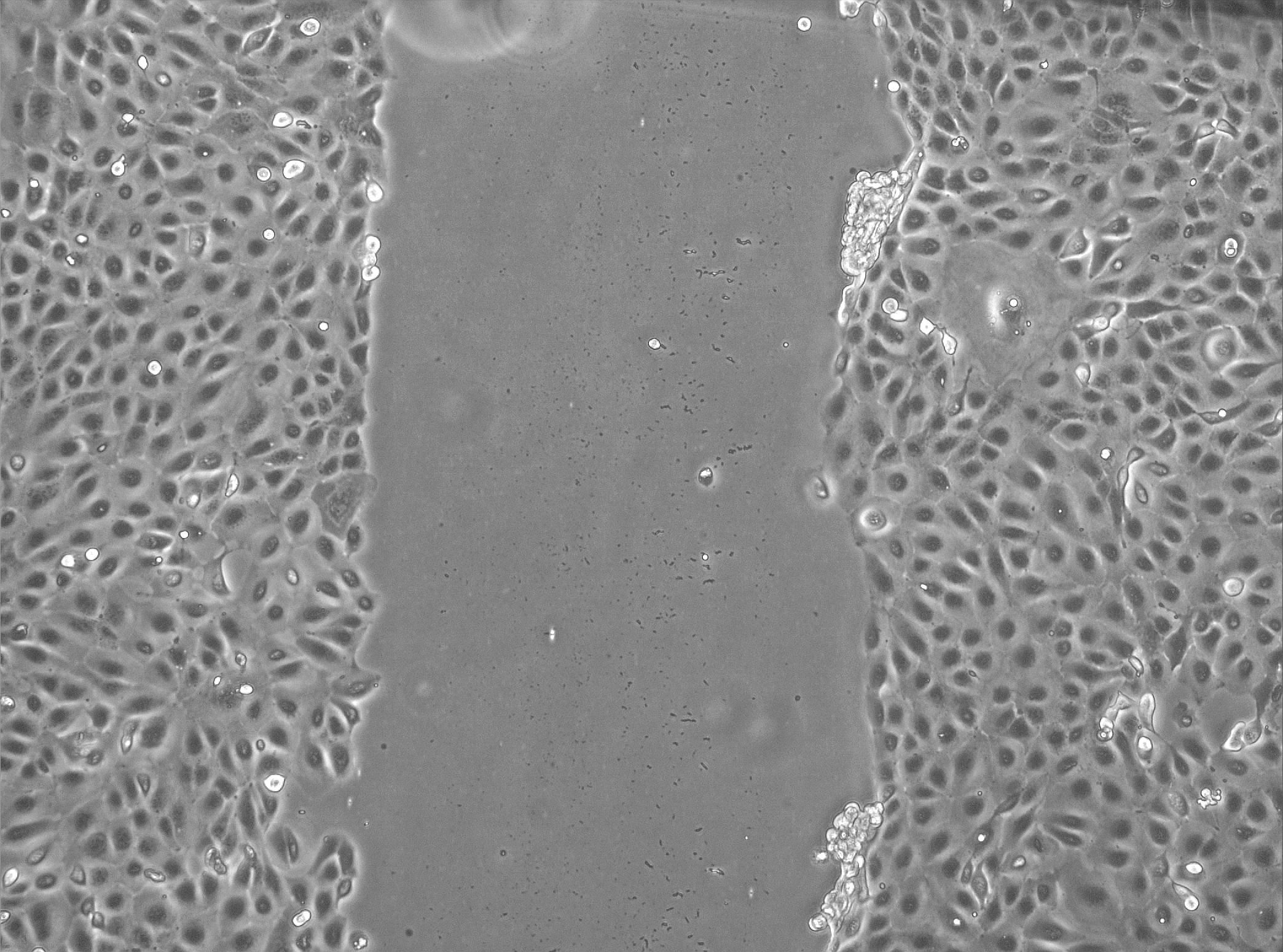

Supplement: S1 Dataset — (ZIP) [file pone.0214184.s001.zip › raw data/Figure 1D raw data/EtOH 4 position 1 t=0.jpg]

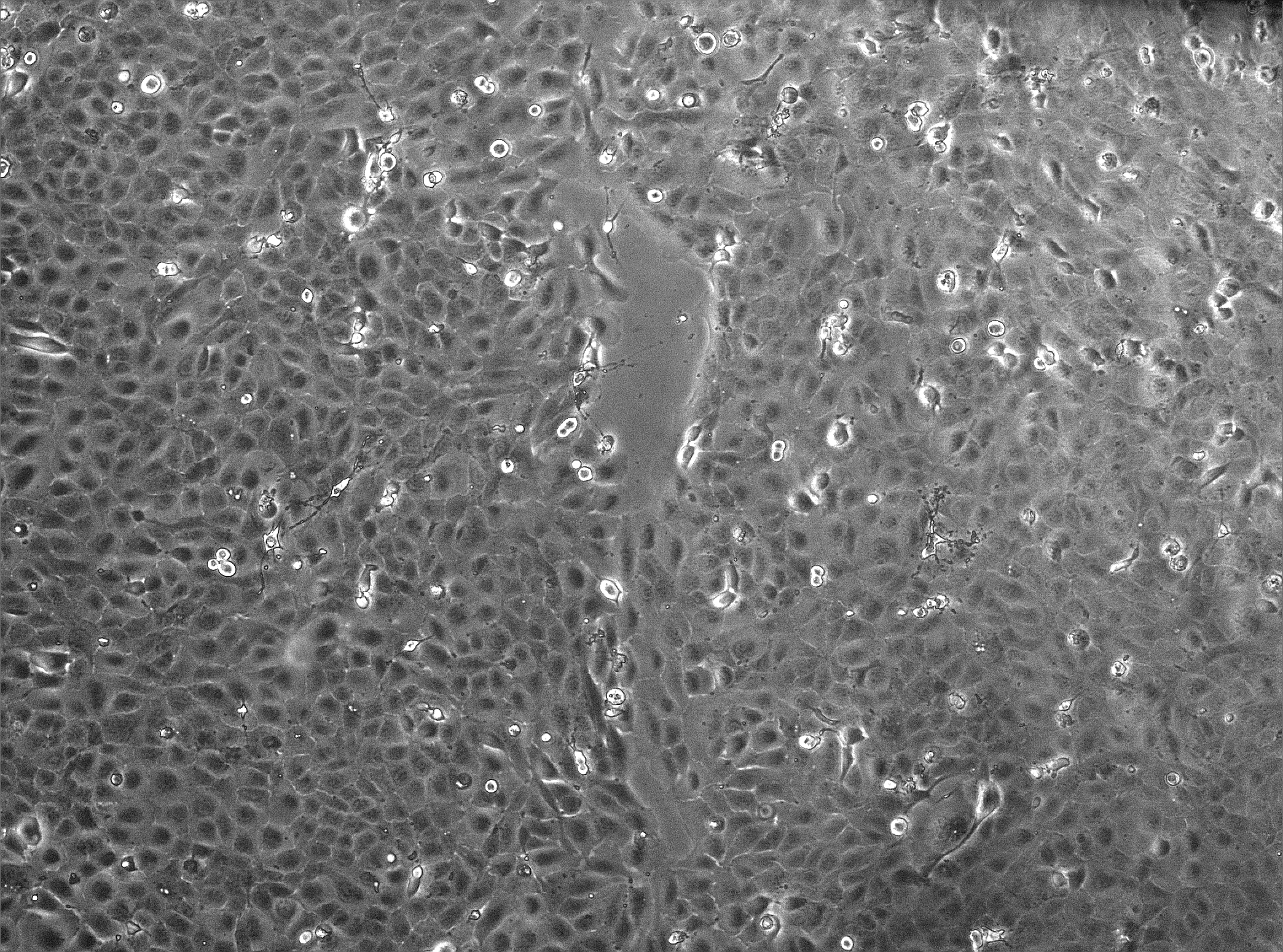

Supplement: S1 Dataset — (ZIP) [file pone.0214184.s001.zip › raw data/Figure 1D raw data/EtOH 4 position 1 t=16.jpg]

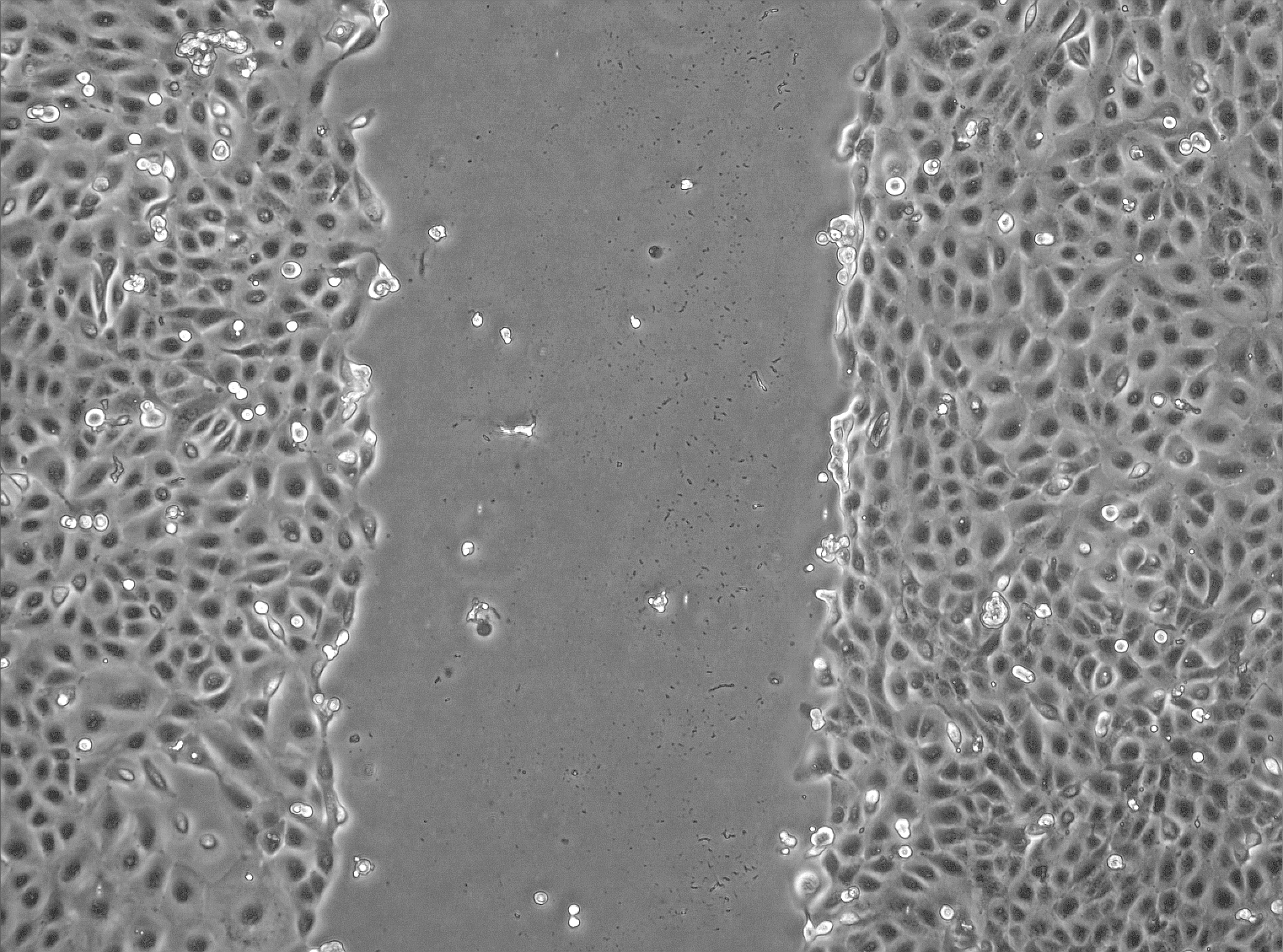

Supplement: S1 Dataset — (ZIP) [file pone.0214184.s001.zip › raw data/Figure 1D raw data/EtOH 4 position 2 t=0.jpg]

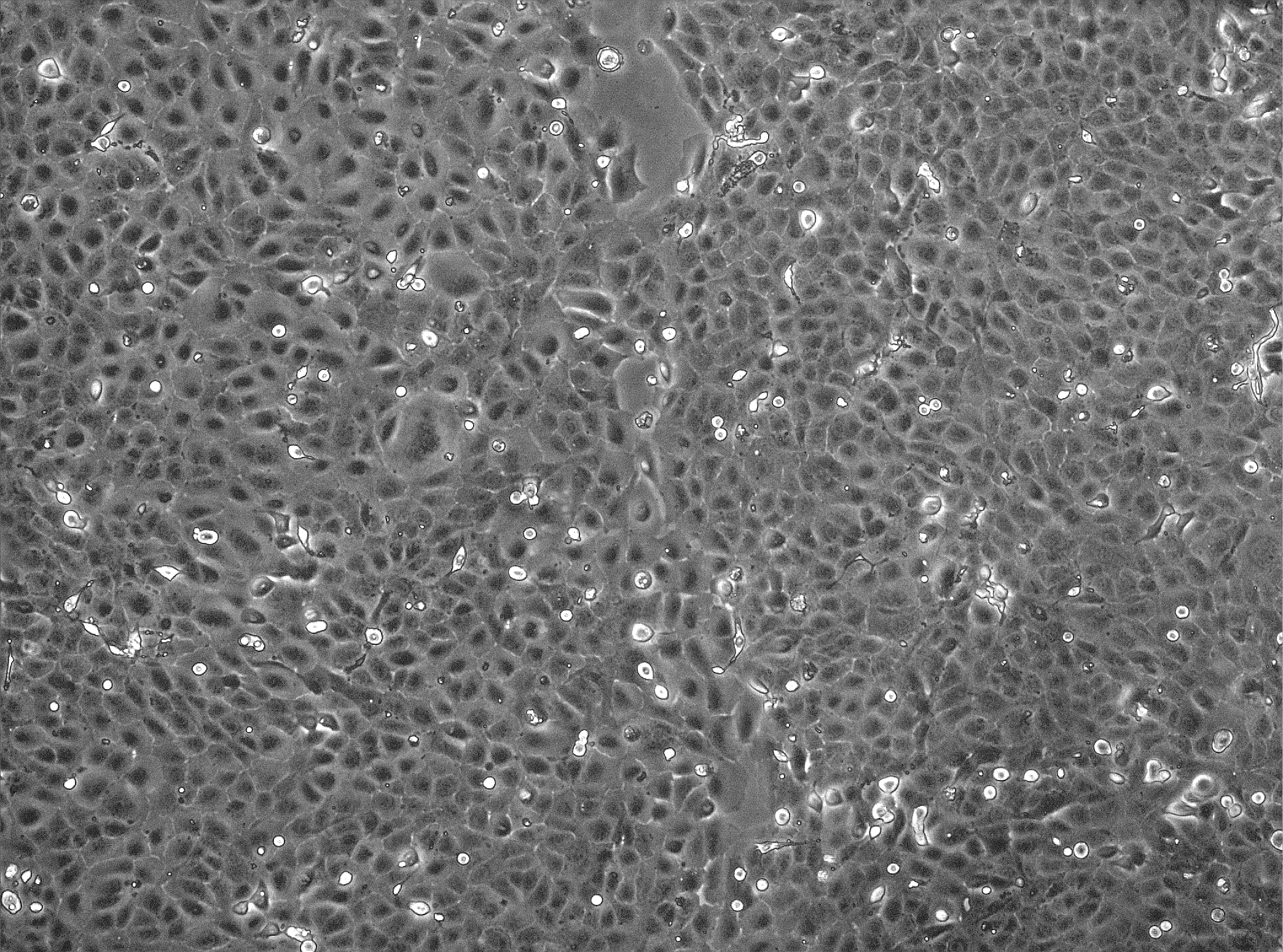

Supplement: S1 Dataset — (ZIP) [file pone.0214184.s001.zip › raw data/Figure 1D raw data/EtOH 4 position 2 t=16.jpg]

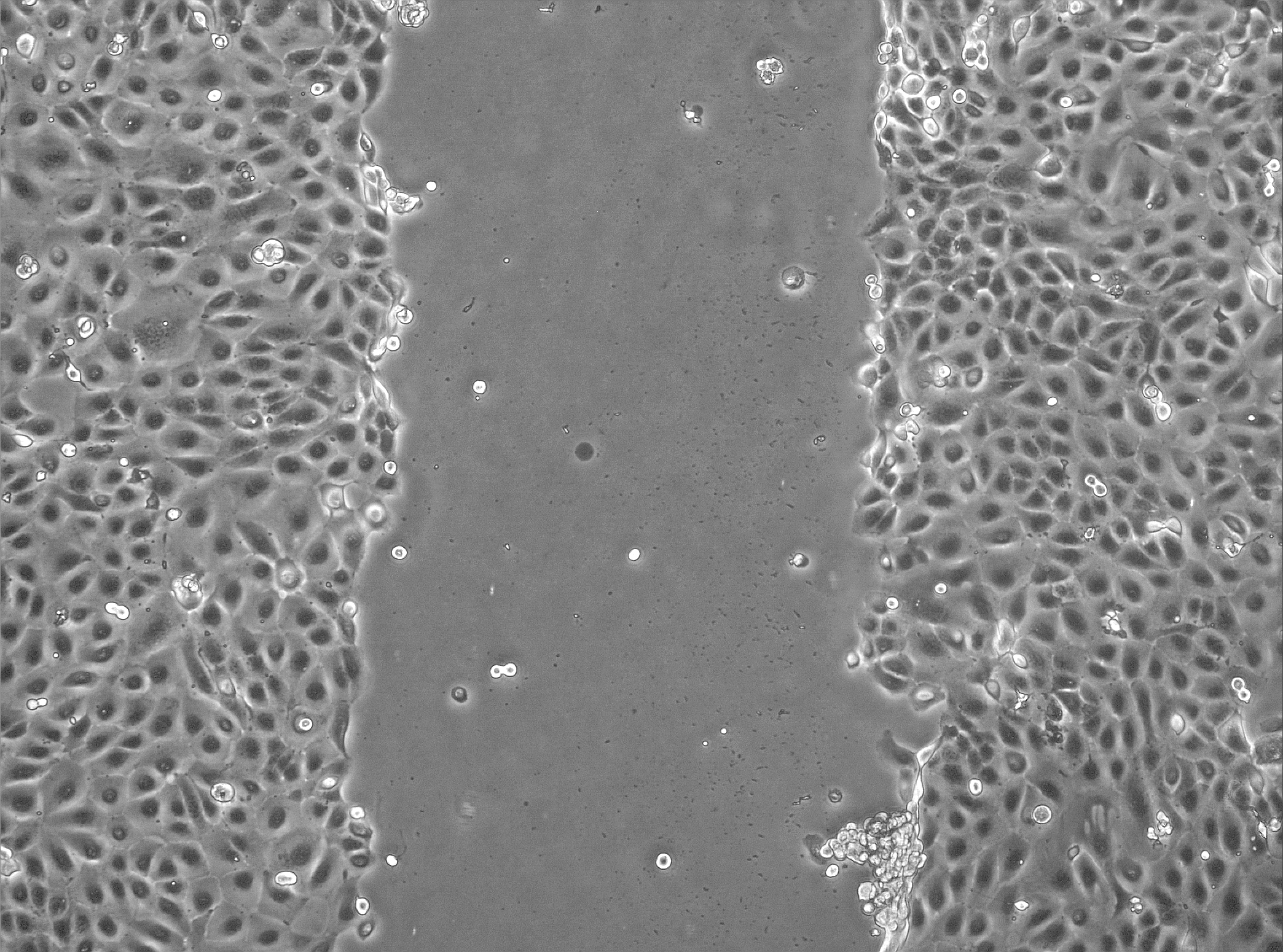

Supplement: S1 Dataset — (ZIP) [file pone.0214184.s001.zip › raw data/Figure 1D raw data/EtOH 4 position 3 t=0.jpg]

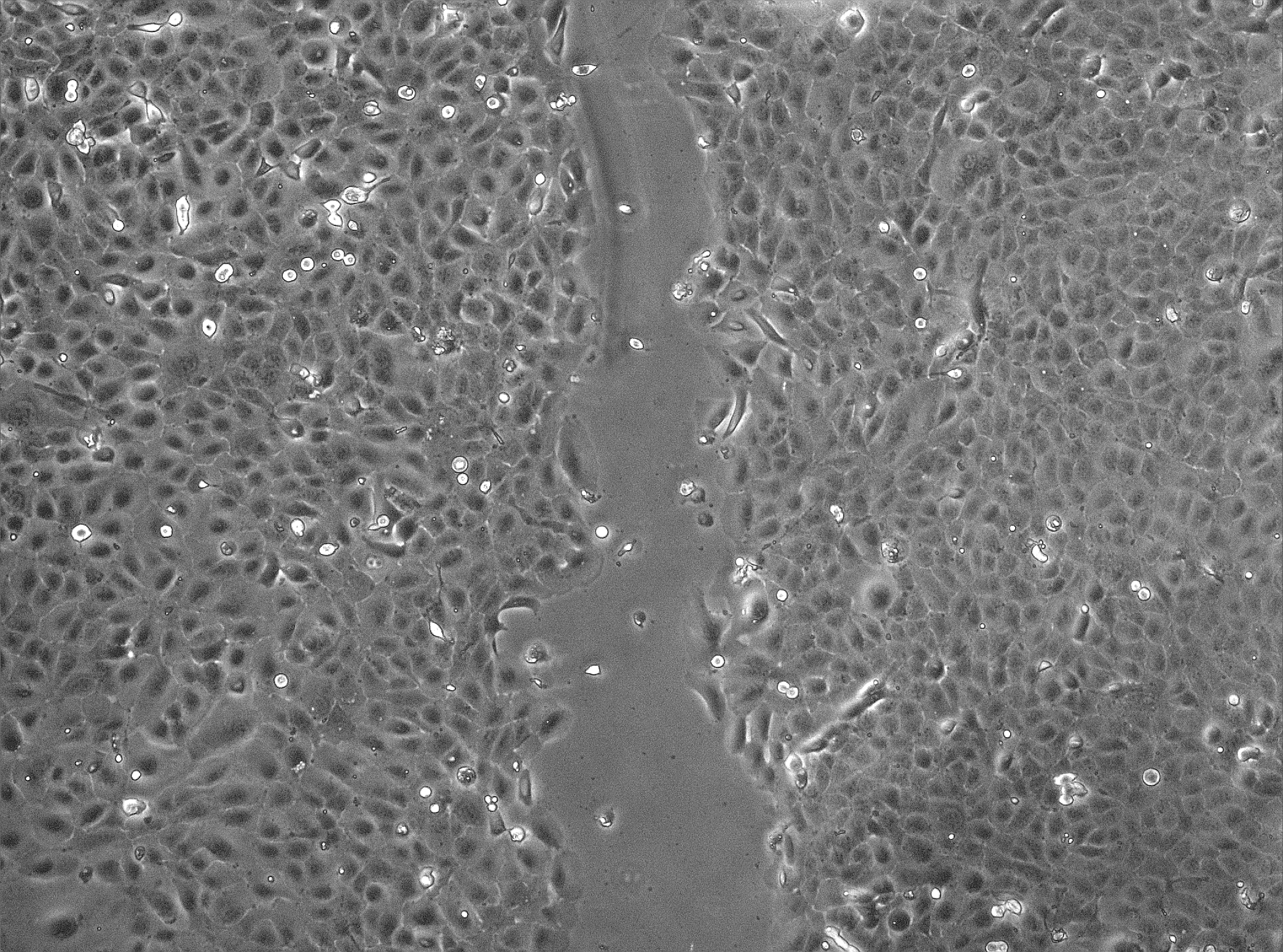

Supplement: S1 Dataset — (ZIP) [file pone.0214184.s001.zip › raw data/Figure 1D raw data/EtOH 4 position 3 t=16.jpg]

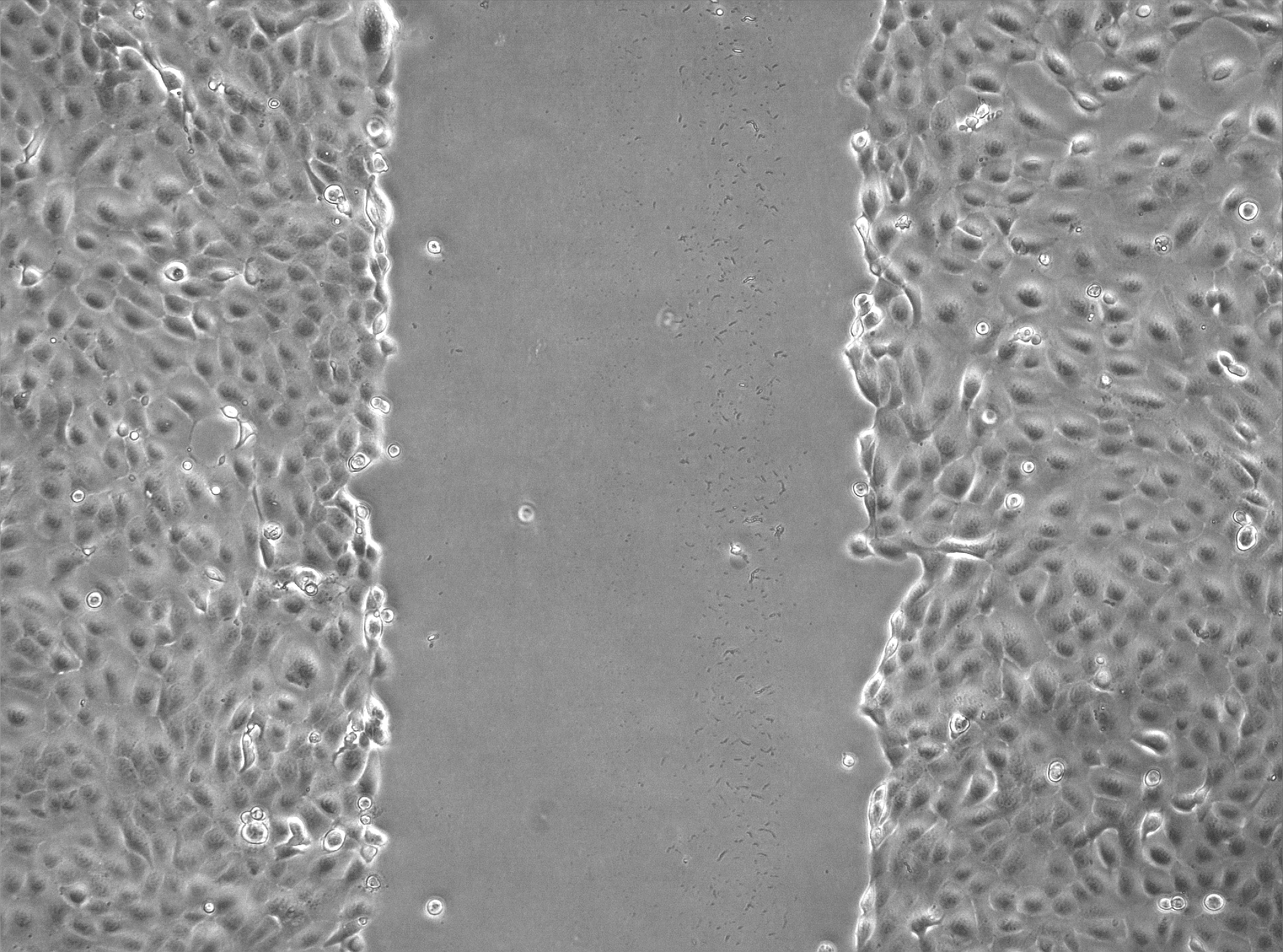

Supplement: S1 Dataset — (ZIP) [file pone.0214184.s001.zip › raw data/Figure 1D raw data/ICI 1 position 1 t=0.jpg]

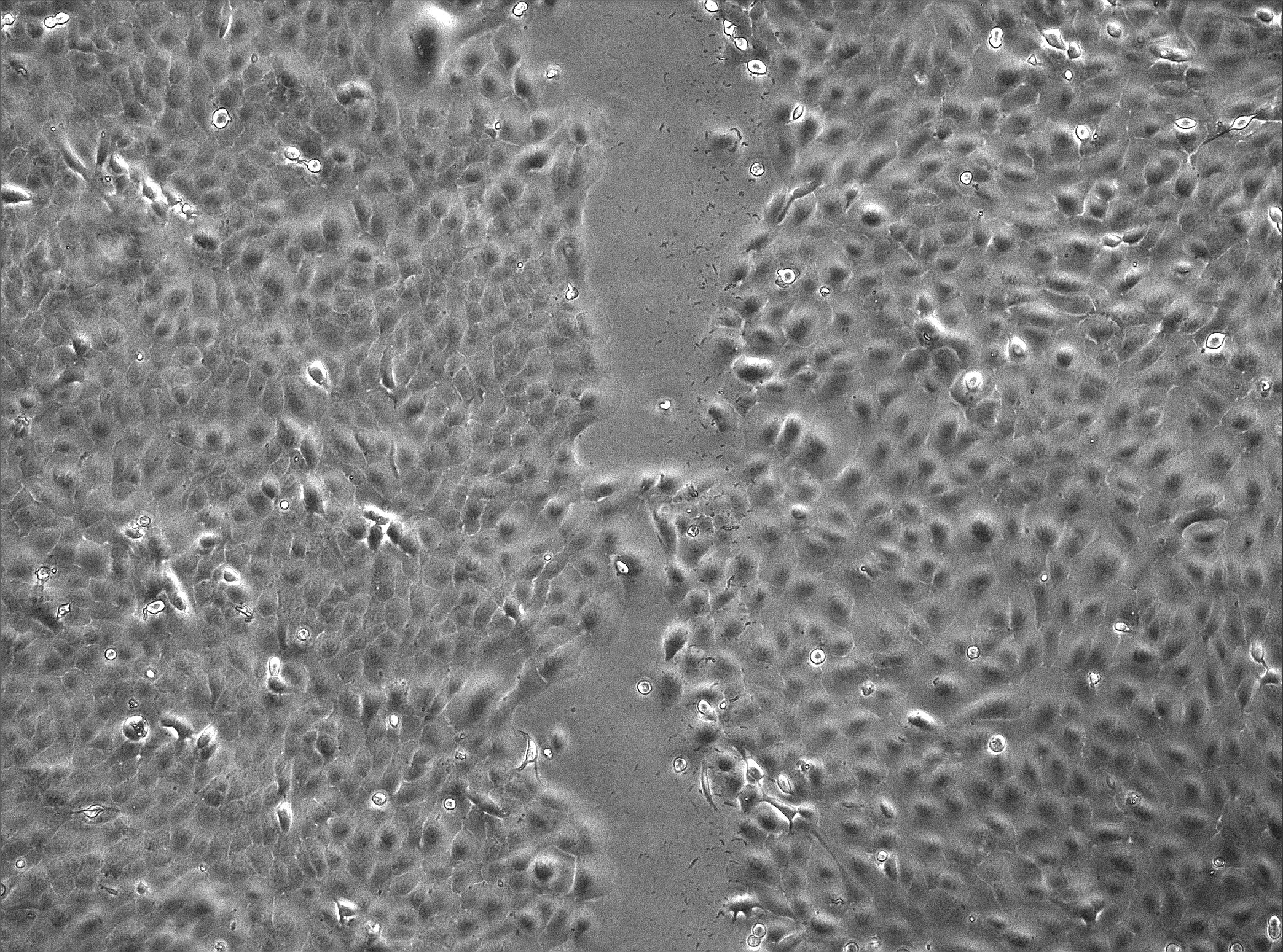

Supplement: S1 Dataset — (ZIP) [file pone.0214184.s001.zip › raw data/Figure 1D raw data/ICI 1 position 1 t=16.jpg]

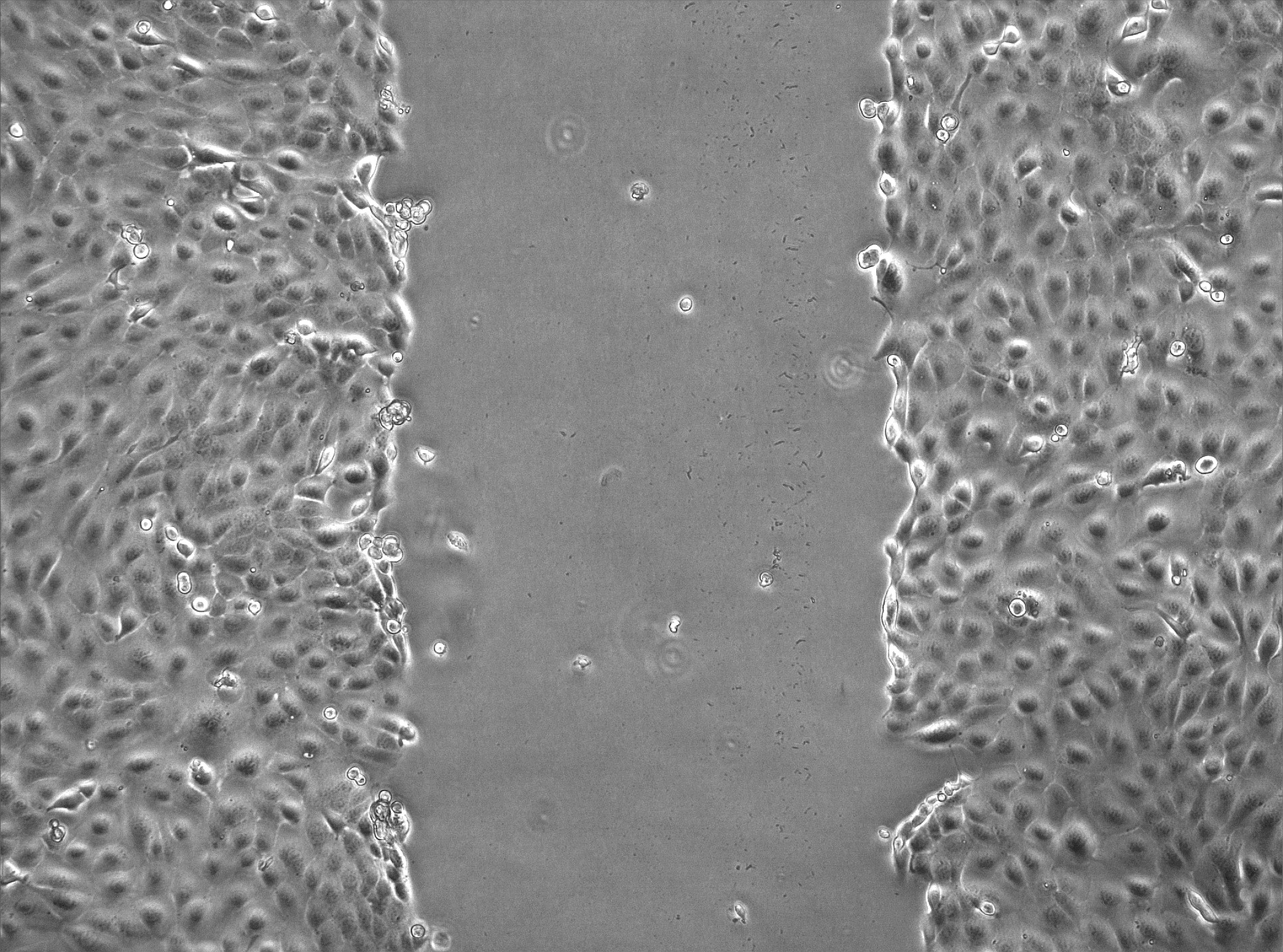

Supplement: S1 Dataset — (ZIP) [file pone.0214184.s001.zip › raw data/Figure 1D raw data/ICI 1 position 2 t=0.jpg]

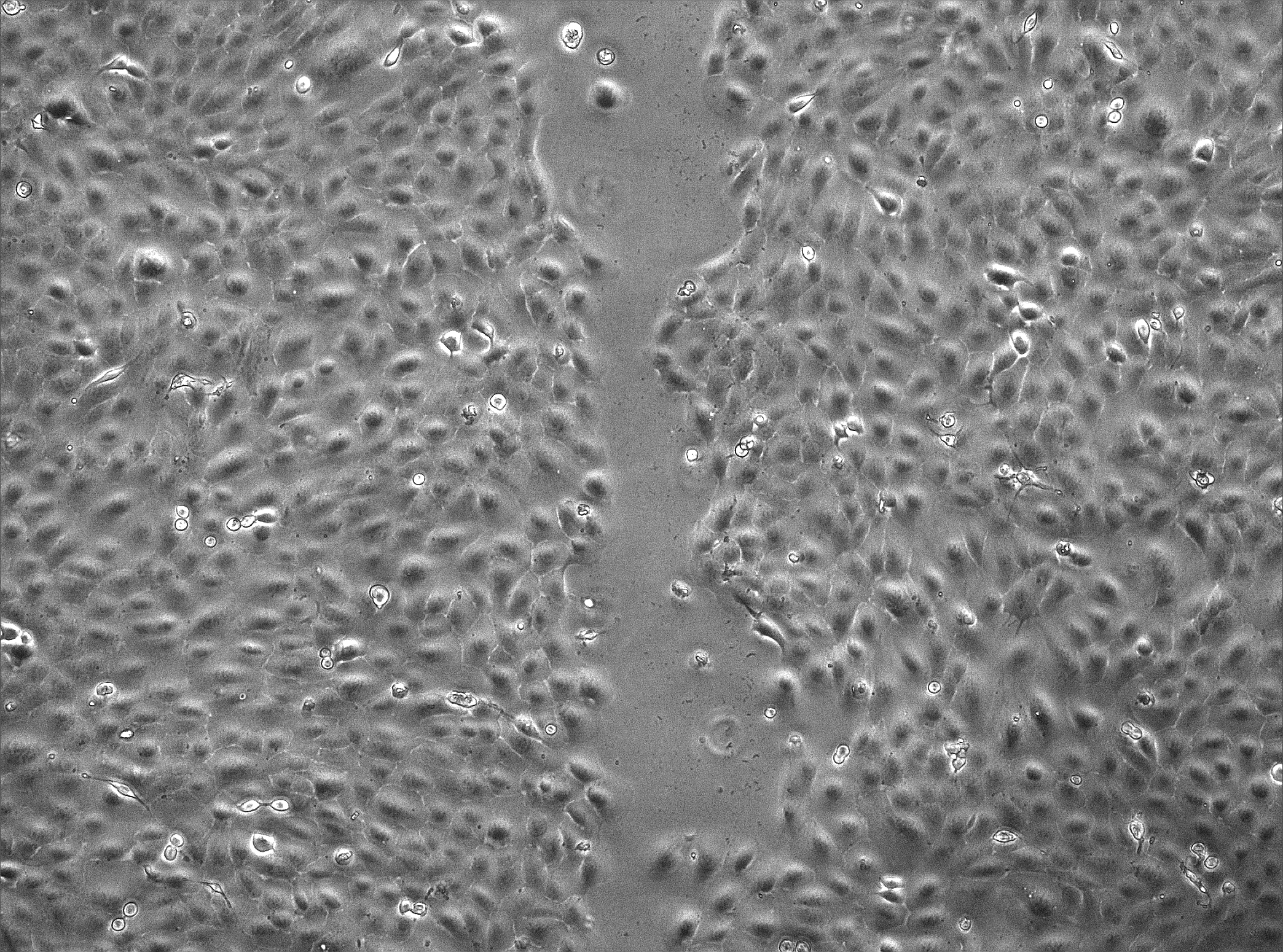

Supplement: S1 Dataset — (ZIP) [file pone.0214184.s001.zip › raw data/Figure 1D raw data/ICI 1 position 2 t=16.jpg]

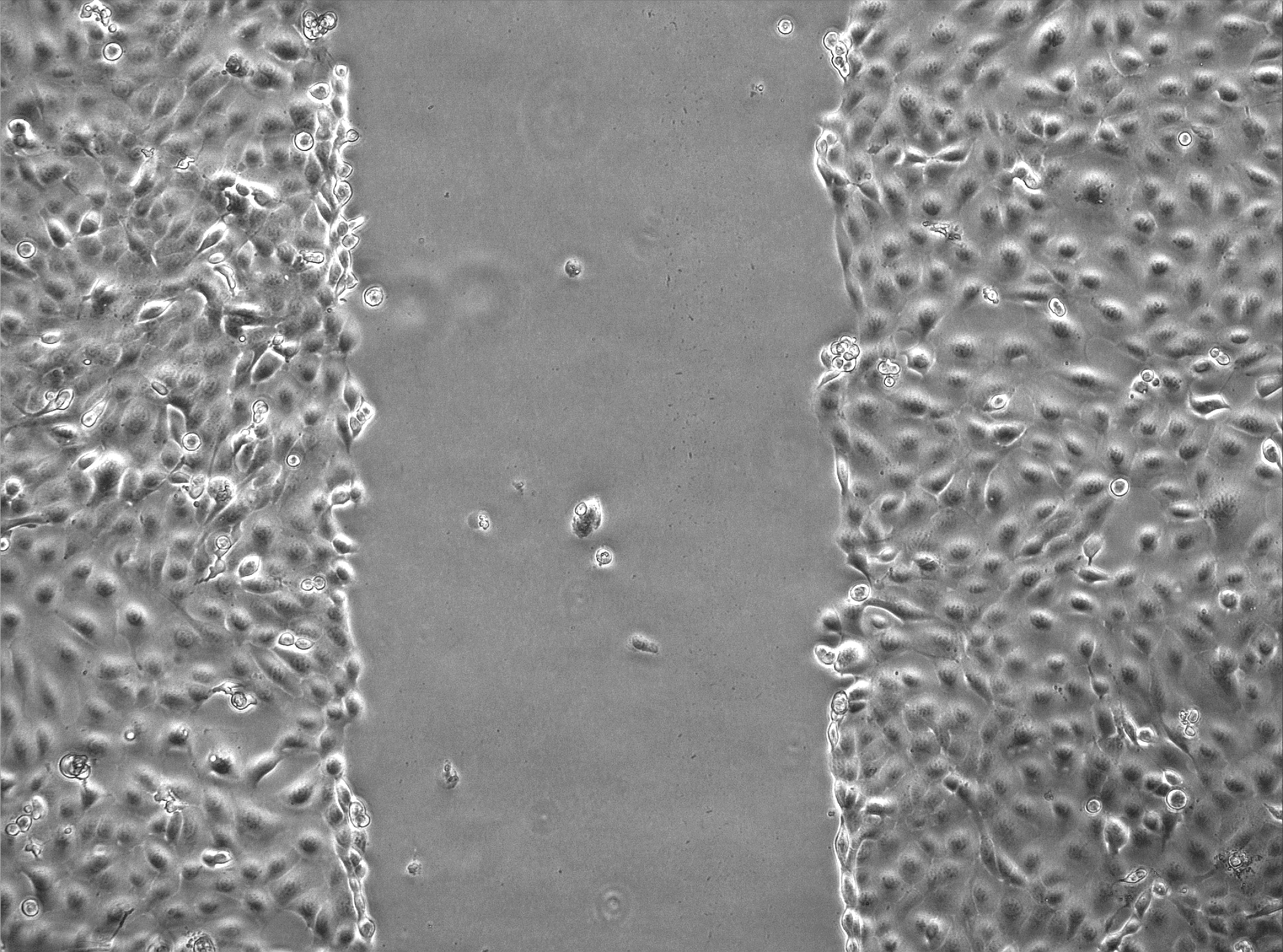

Supplement: S1 Dataset — (ZIP) [file pone.0214184.s001.zip › raw data/Figure 1D raw data/ICI 1 position 3 t=0.jpg]

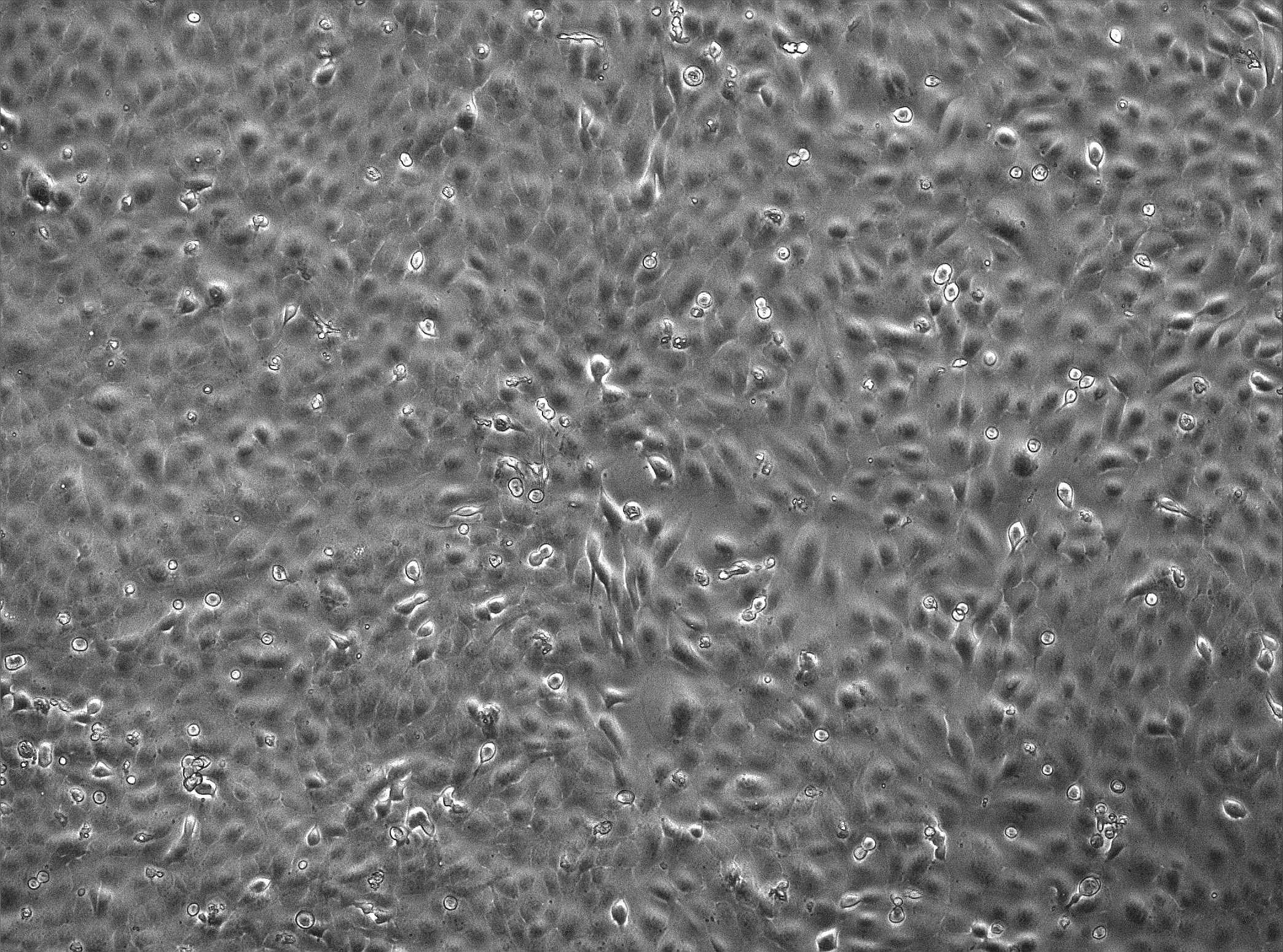

Supplement: S1 Dataset — (ZIP) [file pone.0214184.s001.zip › raw data/Figure 1D raw data/ICI 1 position 3 t=16.jpg]

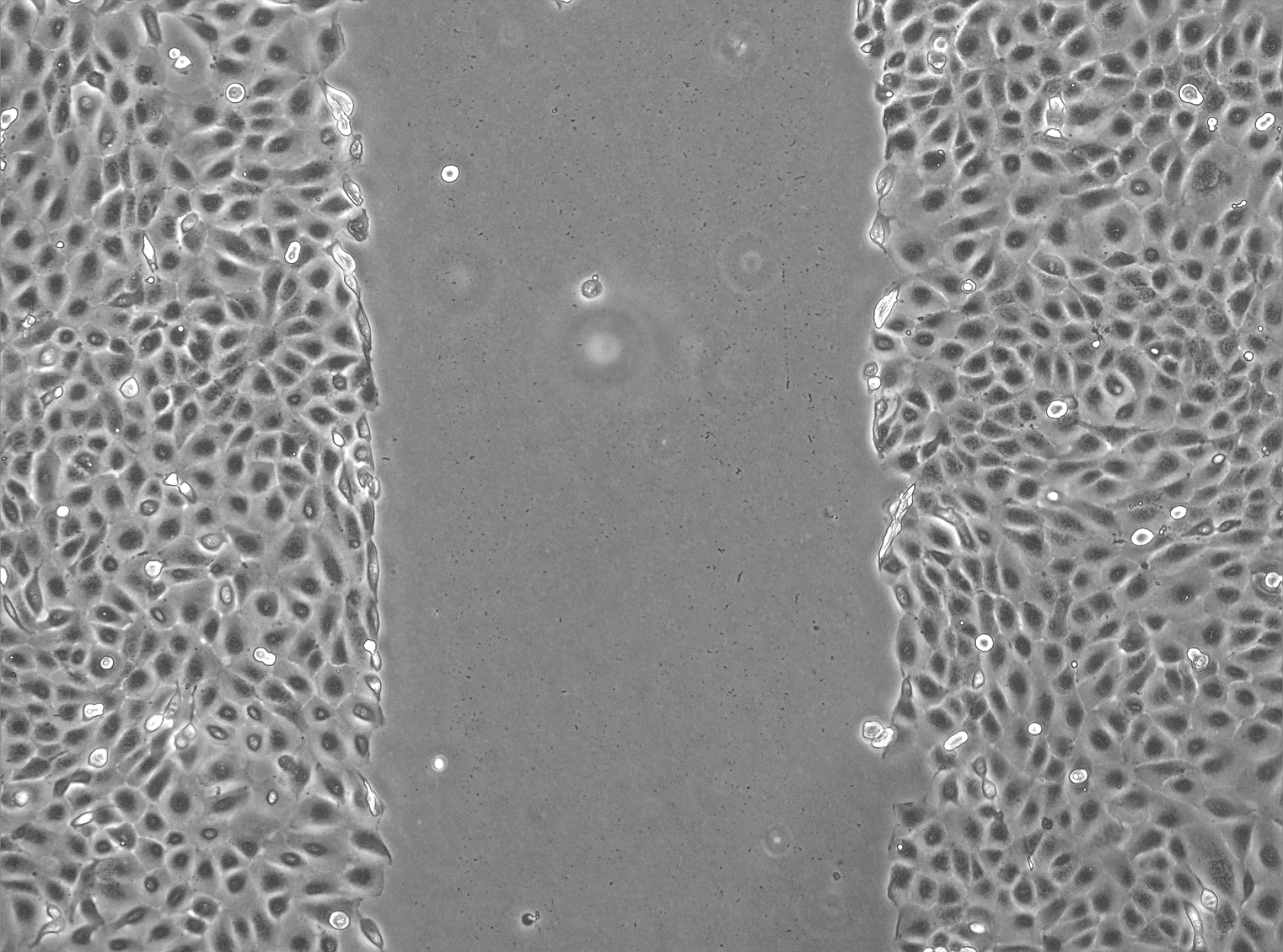

Supplement: S1 Dataset — (ZIP) [file pone.0214184.s001.zip › raw data/Figure 1D raw data/ICI 4 position 1 t=0.jpg]

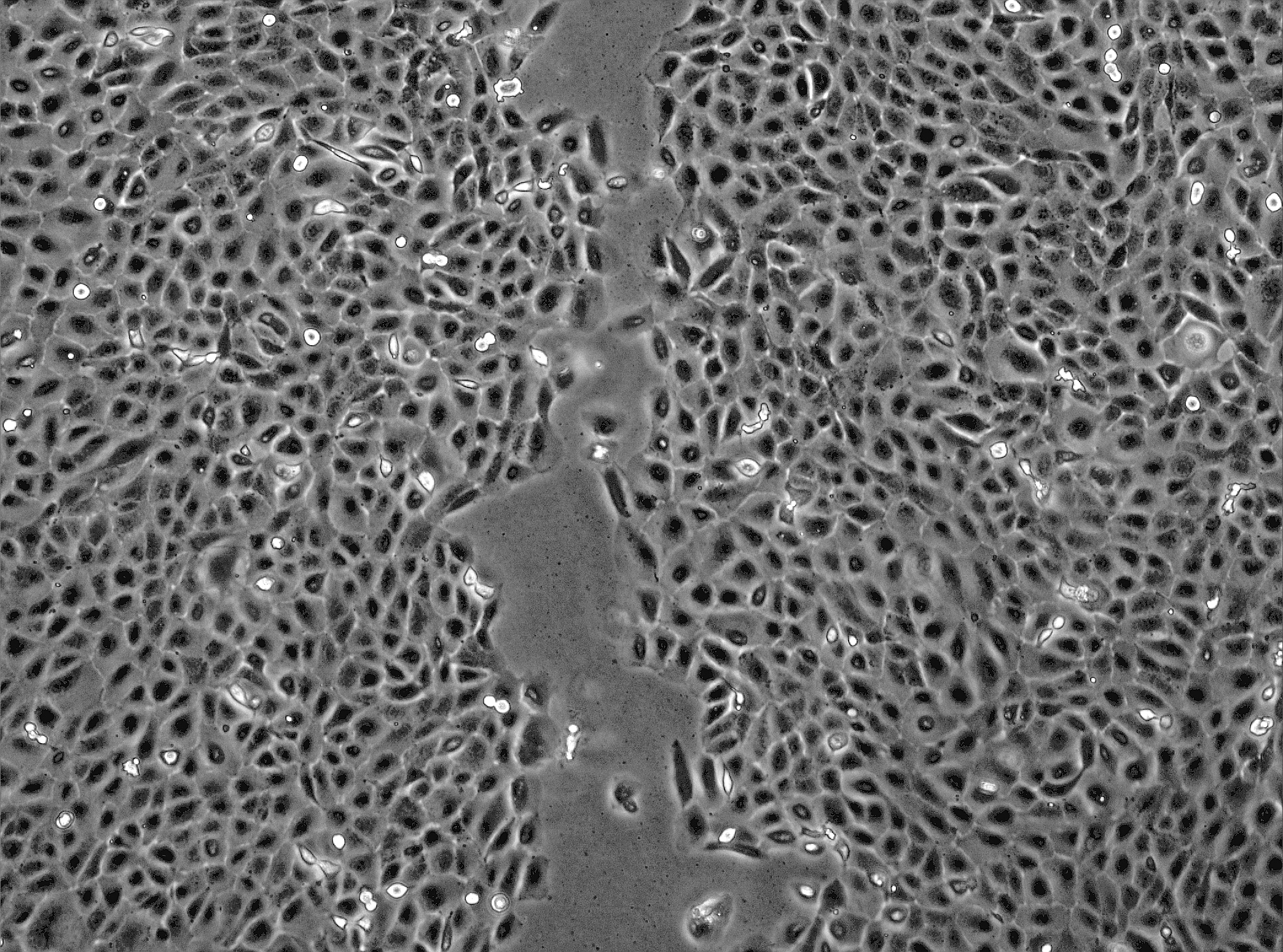

Supplement: S1 Dataset — (ZIP) [file pone.0214184.s001.zip › raw data/Figure 1D raw data/ICI 4 position 1 t=16.jpg]

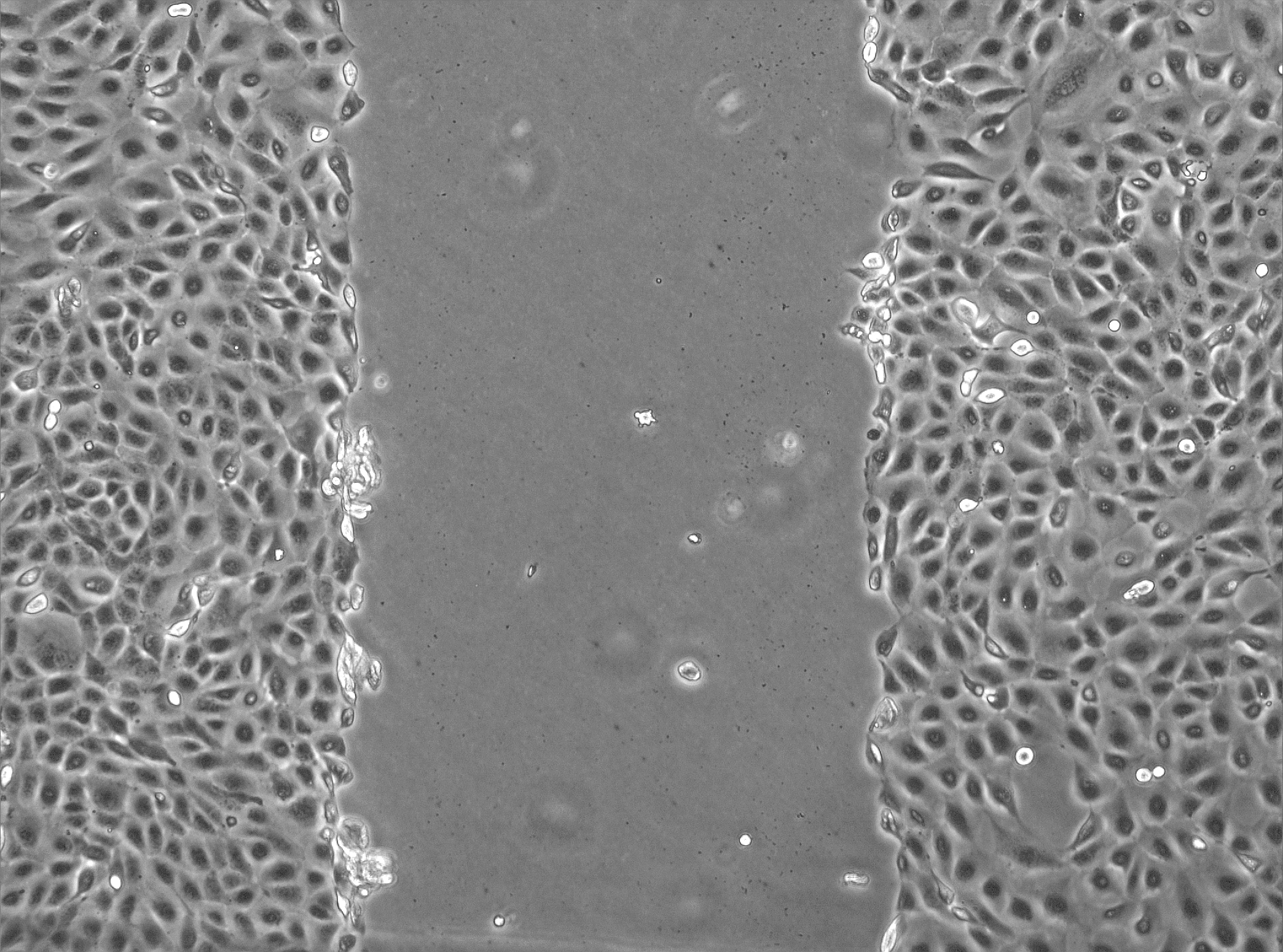

Supplement: S1 Dataset — (ZIP) [file pone.0214184.s001.zip › raw data/Figure 1D raw data/ICI 4 position 2 t=0.jpg]

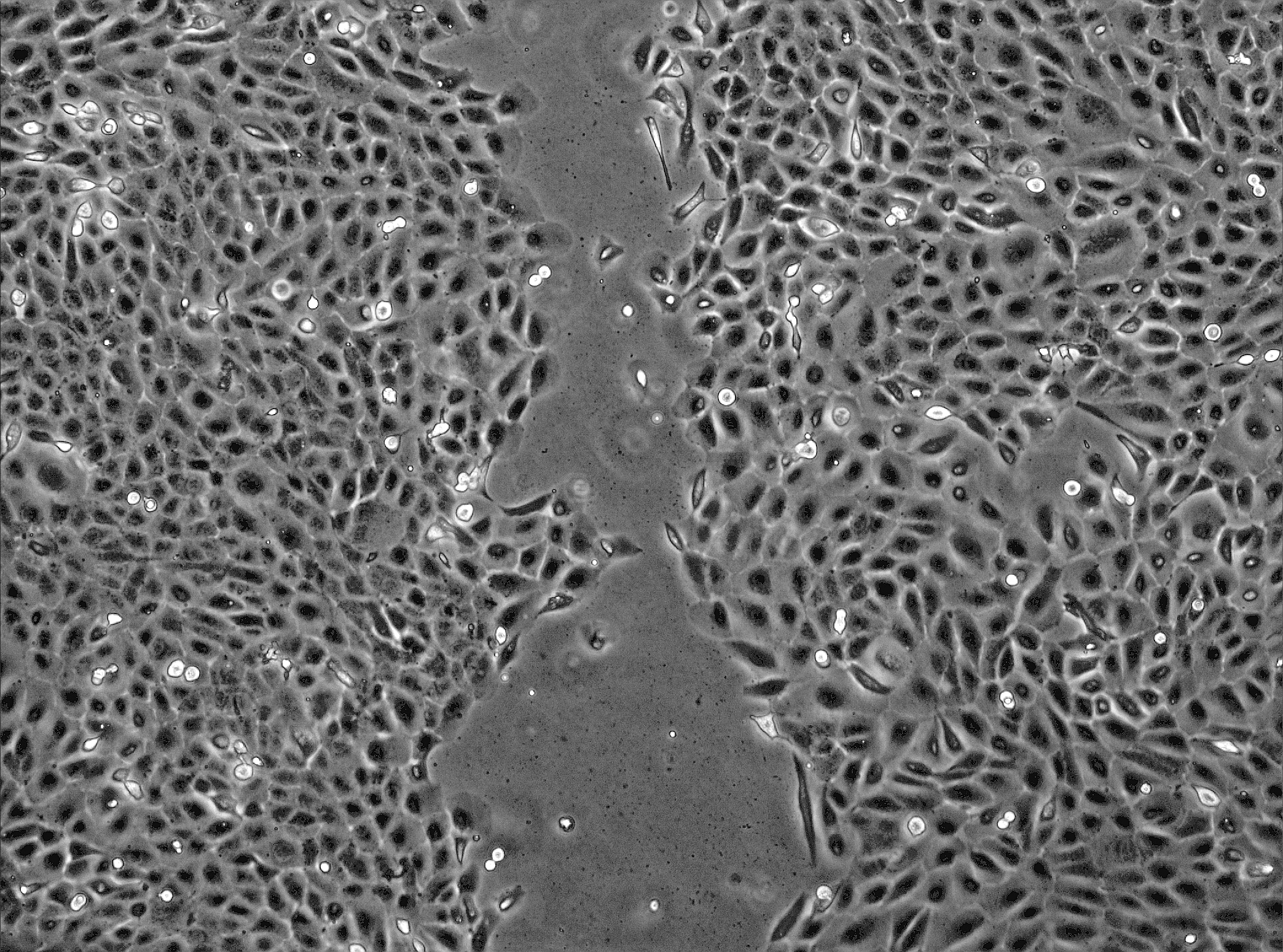

Supplement: S1 Dataset — (ZIP) [file pone.0214184.s001.zip › raw data/Figure 1D raw data/ICI 4 position 2 t=16.jpg]

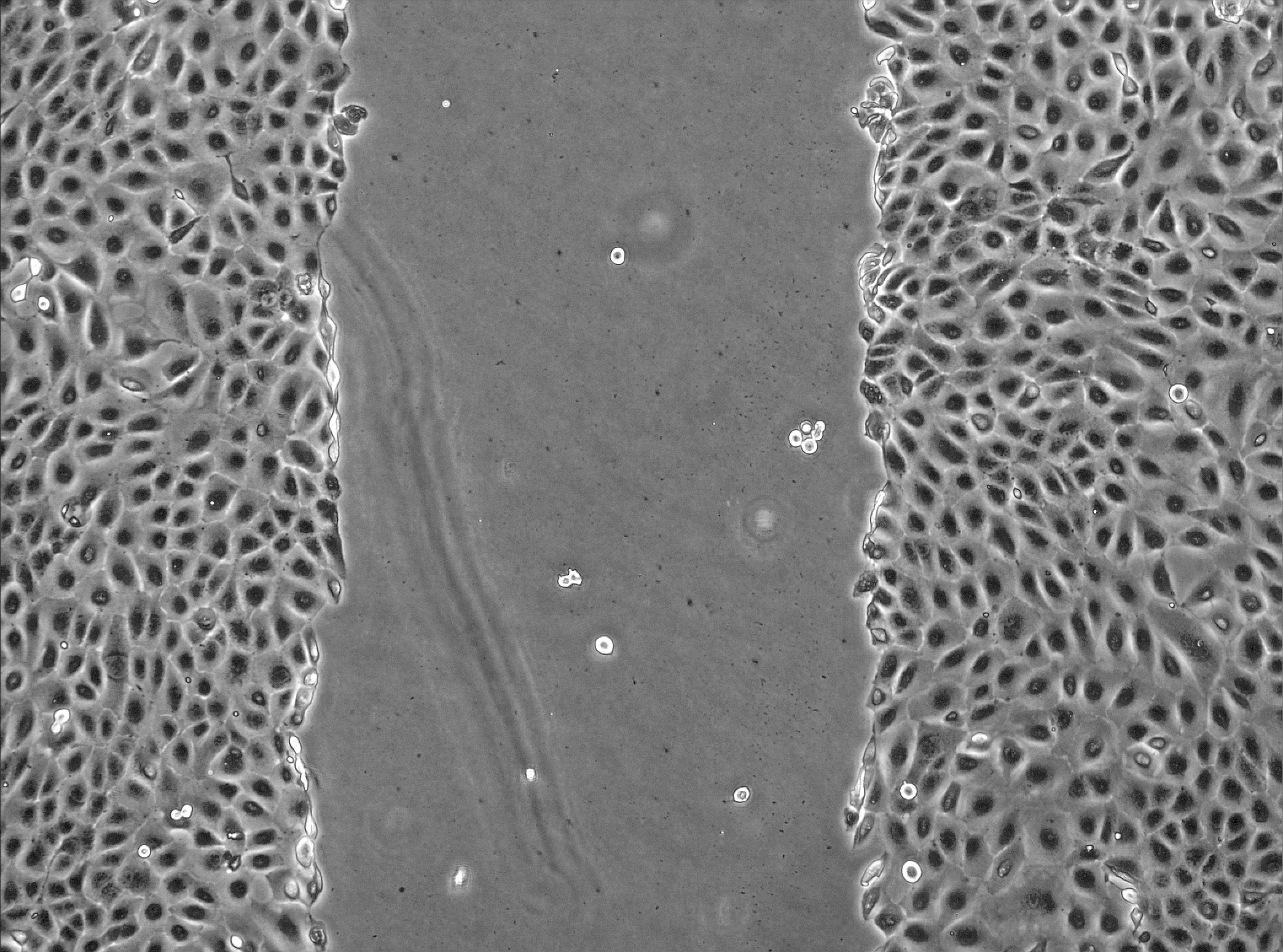

Supplement: S1 Dataset — (ZIP) [file pone.0214184.s001.zip › raw data/Figure 1D raw data/ICI 4 position 3 t=0.jpg]

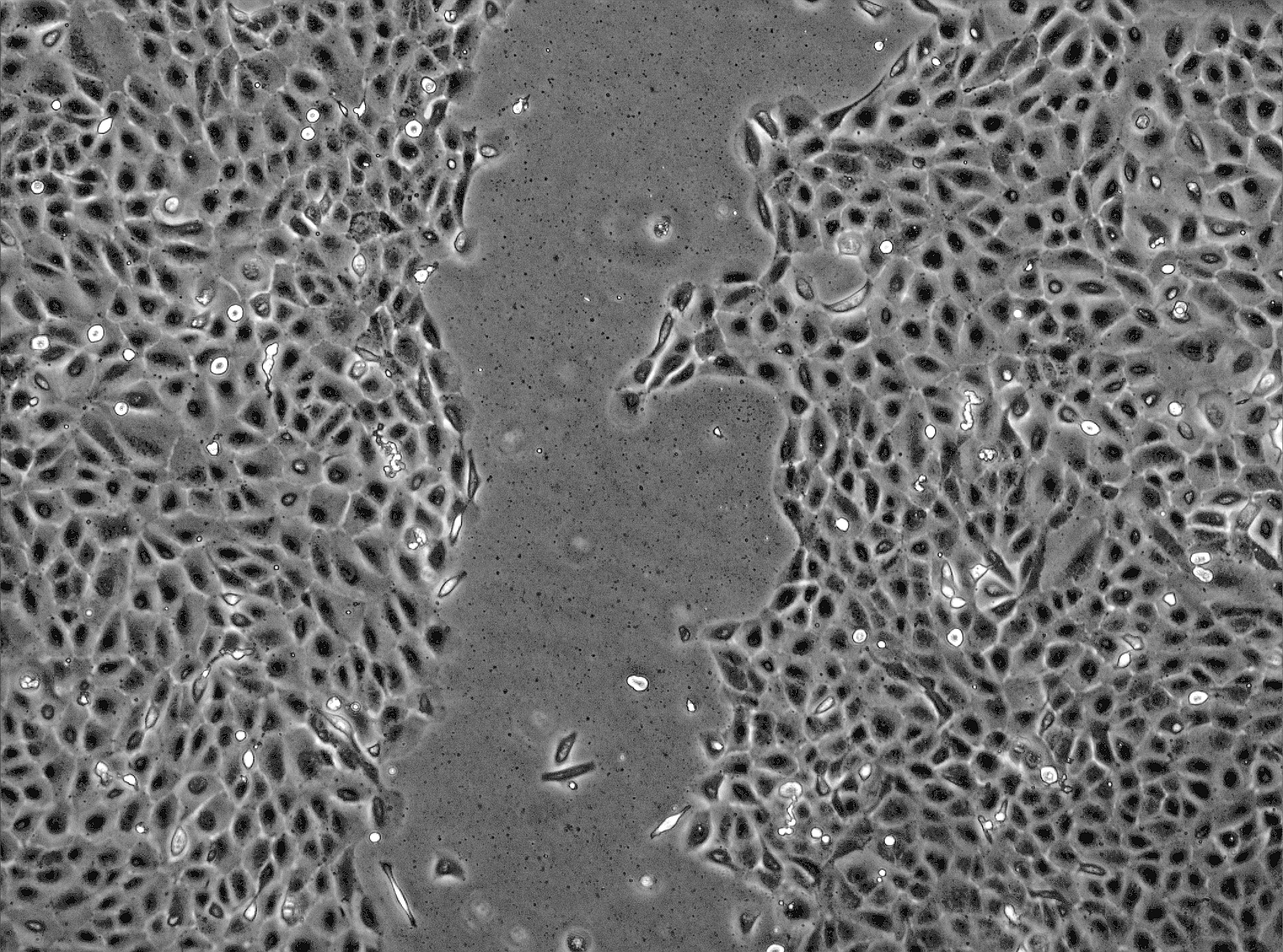

Supplement: S1 Dataset — (ZIP) [file pone.0214184.s001.zip › raw data/Figure 1D raw data/ICI 4 position 3 t=16.jpg]

## Slide 1
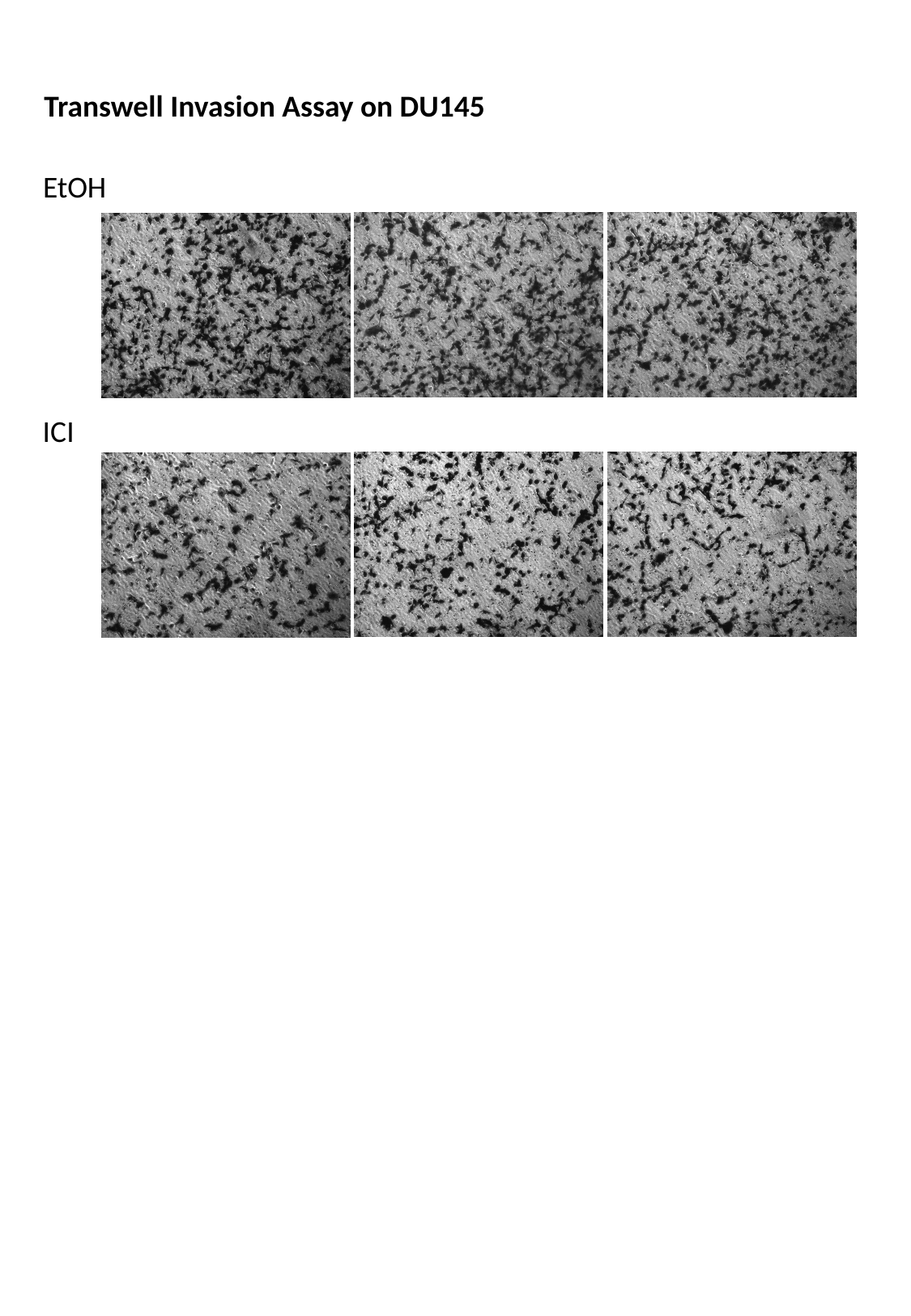

Transwell Invasion Assay on DU145
EtOH
ICI

Supplement: S1 Dataset — (ZIP) [file pone.0214184.s001.zip › raw data/Figure 1E raw data/DU145_ICI_Transwell Invasion Summary-Figure 1E.pptx]

## Slide 1
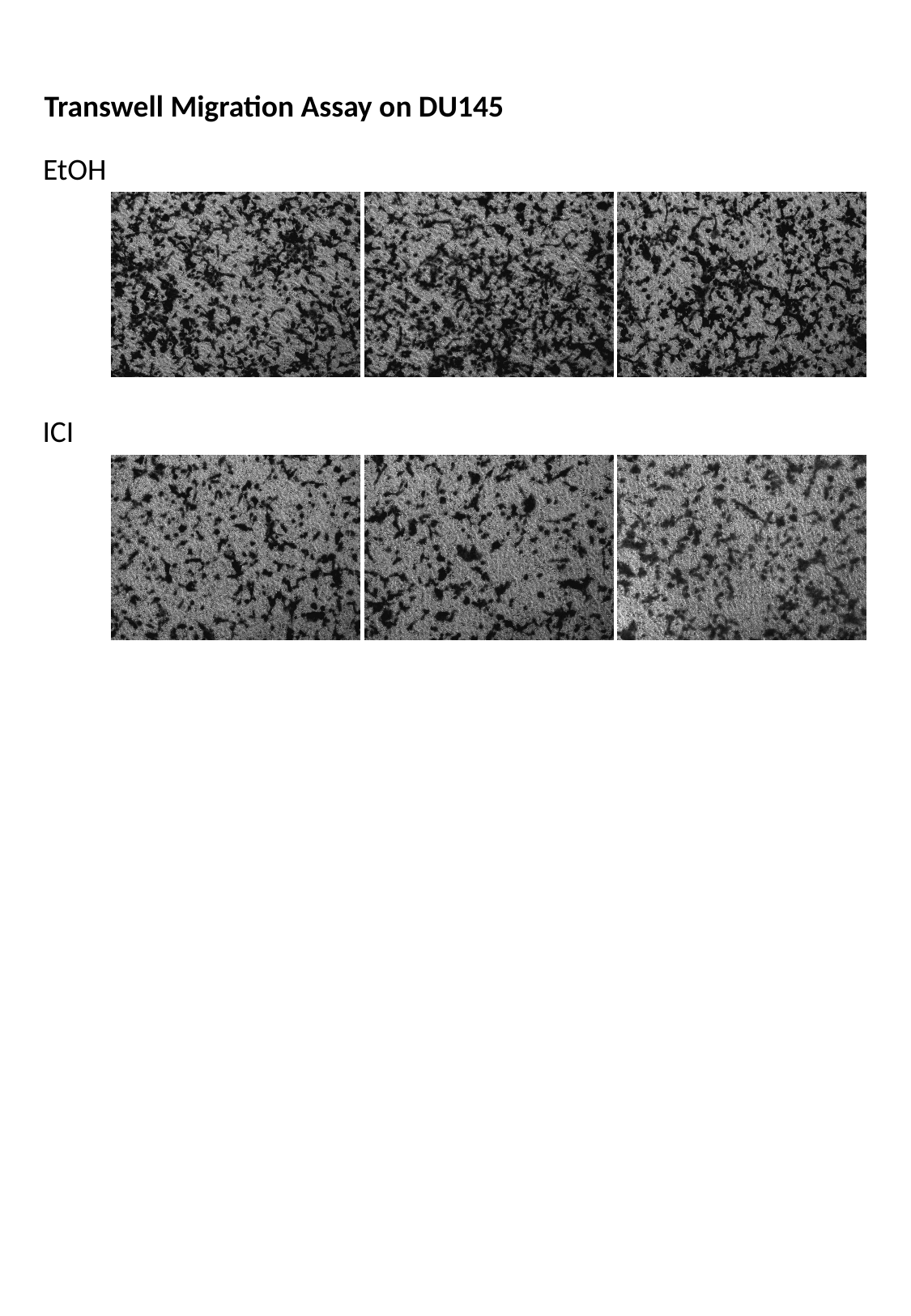

Transwell Migration Assay on DU145
EtOH
ICI

Supplement: S1 Dataset — (ZIP) [file pone.0214184.s001.zip › raw data/Figure 1E raw data/DU145_ICI_Transwell Migration Summary-Figure 1E.pptx]

|  | EtOH+Empty | ICI+Empty |  |  |
| --- | --- | --- | --- | --- |
| 63X | 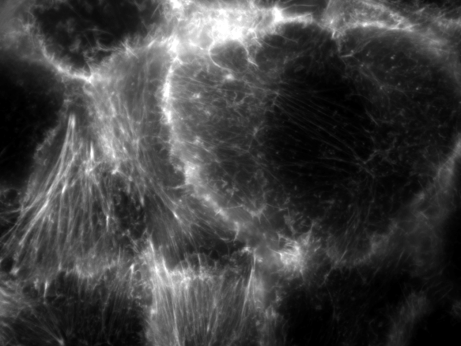 | 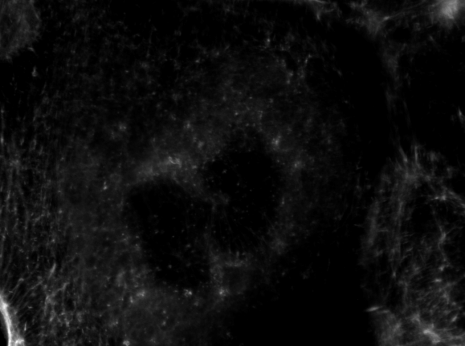 |  |  |
| 100X |  |  |  |  |
|  |  |  |  |  |

Supplement: S1 Dataset — (ZIP) [file pone.0214184.s001.zip › raw data/Figure 1F raw data/ICI_F-actin staining_20110118-Figure 1F.docx]

|  | siERβ+ICI | siERβ+EtOH |
| --- | --- | --- |
| 63X | 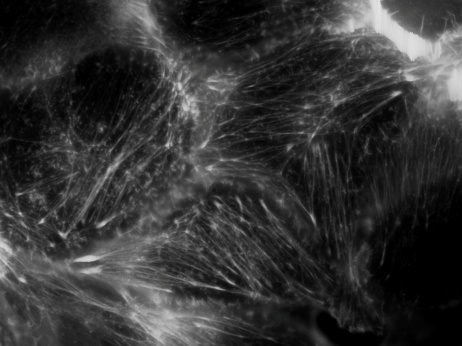 | 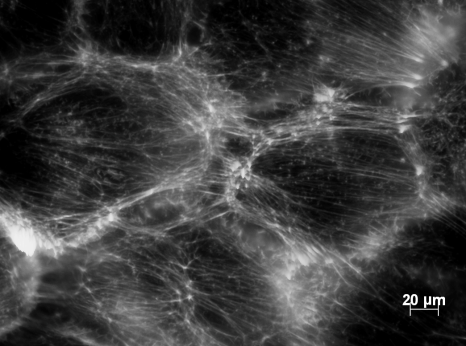 |
| 100X |  |  |
|  |  |  |

Supplement: S1 Dataset — (ZIP) [file pone.0214184.s001.zip › raw data/Figure 1F raw data/ICI_siERb_F-actin staining_20110122b-Figure 1F.docx]
